# Develop Reusable Carbon Sub‐Micrometer Composites with Record‐High Cd(II) Removal Capacity

**DOI:** 10.1002/advs.202408295

**Published:** 2024-11-22

**Authors:** Mengke Cui, Huiting Jiao, Shijie Yuan, Bin Dong, Zuxin Xu

**Affiliations:** ^1^ State Key Laboratory of Pollution Control and Resource Reuse College of Environmental Science and Engineering Tongji University Shanghai 200092 P. R. China; ^2^ Shanghai Institute of Pollution Control and Ecological Security Tongji University Shanghai 200092 P. R. China; ^3^ College of Environmental Science and Engineering Guilin University of Technology Guilin 541006 P. R. China

**Keywords:** enrichment–hydrolysis adsorption mechanism, high economic feasibility, high‐alkalinity adsorption microenvironment, mine drainage, record‐high adsorption capacities, ultrafast adsorption kinetics

## Abstract

Cd(II)‐induced pollution across diverse water bodies severely threatens ecosystems and human health. Nevertheless, achieving ultra‐efficient and cost‐effective treatment of trace amounts of heavy metals remains a major challenge. Herein, the novel carbon sub‐micrometer composites (CSMCs) supported Fe^0^@γ‐Fe_2_O_3_ core‐shell clusters nanostructures are designed and synthesized through a series of universally applicable methods. Research data on adsorption behavior clearly revealed that resorcinol/formaldehyde 1.25‐basic ferric acetate (RF‐1.25BFA) and RF‐1.25BFA‐540 have surprising adsorption capacities. Employing the adsorbent dosage of 0.025 g L^−1^, the adsorption capacities for 10 mg L^−1^ Cd(II) reached 400.00 mg g^−1^ with ultrafast adsorption kinetics, alongside theoretical maximum adsorption capacities for Cd(II) of 1108.87 and 1065.06 mg g^−1^ using 0.025 g L^−1^ adsorbent, respectively, setting a new record‐high level. Additionally, they demonstrated exceptional stability and reusability, maintaining Cd(II) removal efficiency above 95% even after 15 adsorption–desorption cycles. Importantly, this study is the first to unveil a new ultrafast successive two‐step enrichment–hydrolysis adsorption mechanism for Cd(II) removal, emphasizing the critical role played by iron clusters nanostructures in constructing a high‐alkalinity adsorption microenvironment on the surface of the materials. The findings reported pioneered a new avenue for the rational design of high‐performance environmental remediation materials, aiming to overcome the limitations of traditional mine drainage treatment.

## Graphical abstract

The novel carbon sub‐micrometer composites (CSMCs) supported Fe^0^@γ‐Fe_2_O_3_ core‐shell clusters nanostructures exhibited exceptional Cd(II) removal capacity, achieving up to 400.00 mg g^−1^ for 10 mg L^−1^ Cd(II) with ultrafast kinetics and a record theoretical maximum capacity of 1108.87 mg g^−1^. These materials demonstrated remarkable stability and reusability over 15 adsorption–desorption cycles, thereby pioneering advanced environmental remediation materials designed to enhance mine drainage treatment.

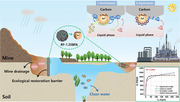

## 1 Introduction

Water quality degradation, particularly in waters polluted with heavy metals, constituted a global risk to both ecological environments and human health, which in turn threatened the sustainable advancement of human societies.^[^ ^1^ ^]^ With the ongoing mining activities, the accumulation of mine waste in natural environments continued to generate drainage, which severely jeopardized the quality of groundwater and surface water resources.^[^ ^2^ ^]^ Mine drainage, even after dilution from rainwater, continued to face challenges associated with heavy metals pollution, notably in the form of trace amounts of heavy metals. The toxicity of heavy metals and their detrimental effects on living organisms, including humans, led to heightened concern over their discharge into aquatic environments, prompting stricter regulatory standards.^[^ ^3^ ^]^ Despite the implementation of numerous measures, heavy metals pollution remained inadequately controlled over the past few decades. Alarmingly, nearly all heavy metals are highly toxic, resistant to biodegradation and thermal degradation, and susceptible to accumulating to dangerous levels.^[^ ^4^ ^]^ Cd(II) ranks among the most toxic metals, causing a range of health issues such as cancer and irreversible kidney dysfunction. Cd(II) pollution in water significantly threatens food safety and human health due to its bioaccumulation within the food chain and prolonged persistence.^[^ ^5^ ^]^ The World Health Organization (WHO) advises that the maximum allowable Cd(II) concentration in drinking water is 0.003 mg L^−1^.^[^ ^6^ ^]^ Even at low levels, Cd(II) can induce free radical formation in the human body, causing damage to proteins, lipids, and DNA, which leads to a variety of health issues.^[^ ^7^ ^]^ Consequently, it is imperative to remove Cd(II) from contaminated water. Given the pervasive nature of Cd(II) pollution and stringent drinking water standards, there is considerable interest in developing effective technologies for Cd(II) removal from contaminated water. The treatment methods presently employed encompass chemical precipitation, adsorption, ion exchange, evaporation, electrodialysis, and reverse osmosis.^[^ ^8^ ^]^ Within the discussed methods, the adsorption technique was deemed an environmentally friendly and competitive approach, distinguished by its flexibility, swift absorption rate, operational simplicity, reusability, and ease of synthesis.^[^ ^9^ ^]^

For an extended period, environmental nanotechnology sparked various expectations and quickly became the focal point for diverse functional developments.^[^ ^10^ ^]^ As a result, there was a pressing requirement for the advancement of efficient and cost‐effective materials for heavy metal removal, with substantial strides having been achieved. Various materials, including biochar,^[^ ^11^ ^]^ minerals,^[^ ^12^ ^]^ graphene oxide,^[^ ^13^ ^]^ nano‐composite materials,^[^ ^14^ ^]^ carbon nanotubes,^[^ ^15^ ^]^ metal oxides and their modified materials,^[^ ^16^ ^]^ have been developed and exhibit the ability to adsorb heavy metals from liquid phases. The functional characteristics of these materials determine the effectiveness of heavy metal removal. Although there have been advancements, the widespread adoption of most materials currently used for treating mine wastewater remains challenging due to persistent drawbacks such as low adsorption capacity, poor transient treatability, inadequate cycle performance, high costs, and restricted general applicability. Their applications were widely constrained by several factors, including preparation efficiency, performance outcomes, and economic viability.^[^ ^17^ ^]^ To mitigate the aforementioned deficiencies, semi‐embedded loaded materials appeared to be a promising strategy. Consequently, many scholars were dedicated to exploring new effective adsorbents with the hope of achieving greater potential in practical engineering application.^[^ ^18^ ^]^ Recent research had shown that structural variations in carbon‐based nanomaterials could result in notable and unexpected changes in their physical and chemical properties.^[^ ^19^ ^]^ Examples included alterations in the coercivity of magnetic materials,^[^ ^20^ ^]^ improvements in surface reactivity and catalytic activity,^[^ ^21^ ^]^ enhancements in regenerative ability,^[^ ^22^ ^]^ changes in electrochemical performance,^[^ ^23^ ^]^ and improvements in mechanical strength.^[^ ^24^ ^]^ Regarding structural issues, the surface effects of carbon‐based nanomaterials had proven to be of utmost importance.^[^ ^25^ ^]^ Briefly, the surface chemistry of carbon‐based nanomaterials, when reduced to a more uniform nanoscale, differed from that at the microscale, revealing unique reaction chemistry.^[^ ^26^ ^]^ This facilitated the manipulation of material properties through surface effects and chemical reactions approached near stoichiometry.^[^ ^27^ ^]^ Hence, it was necessary to scientifically describe the structure‐function relationship of nanomaterials based on their fundamental chemical properties. Typically, failure frequently occurred on the surface of carbon‐based nanomaterials. Thus, optimizing the surface structure could effectively enhance the overall performance of carbon‐based nanomaterials.^[^ ^28^ ^]^ We sought to explore in greater depth the structure‐function relationship of carbon‐based nanomaterials in order to uncover the subtle interactions between trace amounts of heavy metals and novel adsorbents.

This study proposed a new ultrafast successive two‐step enrichment–hydrolysis adsorption mechanism, distinct from conventional heavy metal adsorption mechanisms, to attain highly efficient removal of trace amounts of Cd(II). A variety of universally applicable methods were used to design and synthesize a novel CSMCs supported Fe^0^@γ‐Fe_2_O_3_ core‐shell clusters nanostructures. A thorough characterization of CSMCs was conducted, covering their adsorption capacity, kinetics, isotherms, techno‐economic assessment, along with validation of their reusability. Due to its superior comprehensive performance, CSMCs was anticipated to become a promising environmental remediation material for the ultra‐efficient and economical treatment of trace amounts of heavy metals, with potential applications in areas such as industrial wastewater, acid mine drainage, and groundwater quality improvement.

## 2 Results and Discussion

### 2.1 Physical and Chemical Properties of the CSMCs

**Figure 1:**
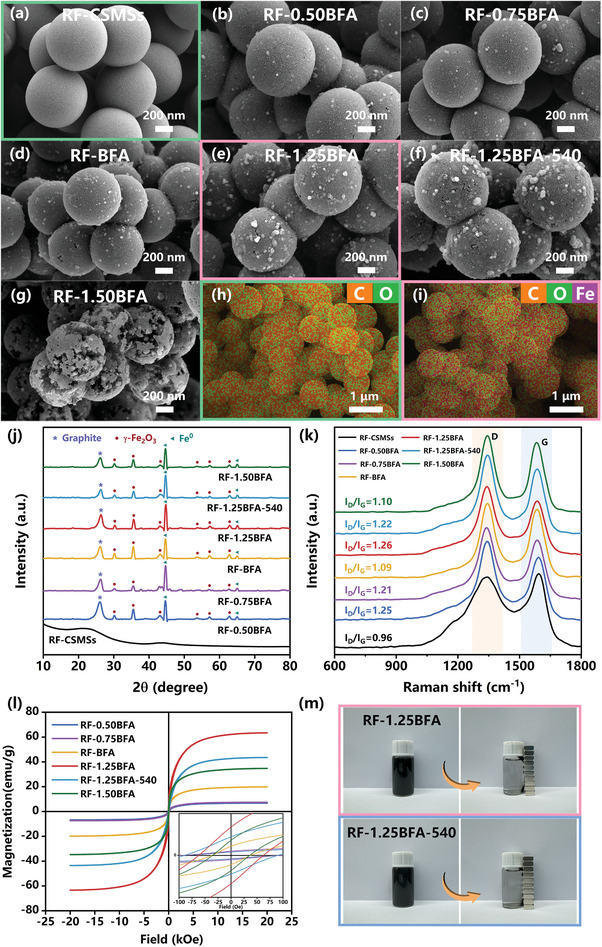
Field emission scanning electron microscopy (FE‐SEM) images of a) resorcinol/formaldehyde carbon sub‐micrometer spheres (RF‐CSMSs), b) RF‐0.50BFA, c) RF‐0.75BFA, d) RF‐BFA, e) RF‐1.25BFA, f) RF‐1.25BFA‐540, and g) RF‐1.50BFA. Energy dispersive spectrometry (EDS) mapping images of the h) RF‐CSMSs and i) RF‐1.25BFA. j) X‐ray diffraction (XRD) patterns, k) Raman spectra, and l) Magnetic hysteresis loops of the CSMCs. The inset presents an enlarged view of hyteresis loops near zero field, covering a range of −100–100 Oe. m) Optical photographs of the magnetic separation of RF‐1.25BFA and RF‐1.25BFA‐540.

All relevant preparations and experimental sections of CSMCs were detailed in Text S1–S3 (Supporting Information). The surface morphologies and microstructures of the CSMCs were analyzed by FE‐SEM. The SEM image (**Figure** **1**a) clearly shows that the RF‐CSMSs retain a uniform and smooth spherical morphology with diameters of 750–900 nm (Figure S1a, Supporting Information). The EDS mapping images (Figure 1h; Figure S2a_1_–a_2_, Supporting Information) results clearly confirmed that C and O elements were uniformly distributed across RF‐CSMSs. The surface microstructure of RF‐xBFA consisted of numerous nanostructured clusters, which varied with the amount of BFA added. As observed in Figure 1b–g, the RF‐xBFA and RF‐1.25BFA‐540 maintained the original spherical morphology of RF‐CSMSs. With lower BFA (mass ratios of 0.5:1, 0.75:1 and 1:1), the clusters nanostructures semi‐embedded on the surface of CSMCs were few and small (Figure 1b–d). With increasing BFA content (the mass ratio = 1.25:1), RF‐1.25BFA continued to maintain its spherical morphology, and the clusters nanostructures continued to grow on the carbon surface. The number of clusters nanostructures on the surface increased, and their size also enlarged (Figure 1e). The EDS mapping images of RF‐1.25BFA (Figure 1i; Figure S2b_1_–b_3_, Supporting Information) showed that C, O and Fe were uniformly distributed. As depicted in Figure S1b (Supporting Information), the diameters of RF‐1.25BFA were between 750 and 950 nm. The surface morphology and microstructure of RF‐1.25BFA demonstrated excellent morphological stability, remaining largely undamaged even after being stored in air for 540 days (Figure 1f). With a further increase in the amount of BFA (the mass ratio = 1.5:1), the surface microstructure of RF‐1.50BFA exhibited significant changes compared to that of RF‐1.25BFA. The spheres appeared cracked and disintegrated, as though they had burst open, giving them a looser and less stable appearance (Figure 1g). This was likely because RF‐1.50BFA produced larger and more numerous iron clusters, which increased the embedded volume and depth on the carbon spheres, ultimately causing them to rupture. As seen in Figure S3 (Supporting Information), the EDS mapping images of RF‐0.50BFA, RF‐0.75BFA, RF‐BFA, RF‐1.25BFA‐540 and RF‐1.50BFA also displayed uniformly distributed C, O and Fe. It is worth noting that this high loading of clusters nanostructures revealed that RF‐1.25BFA and RF‐1.25BFA‐540 were expected to have high adsorption capacity. This property is to be expected and will be further discussed below.

Interestingly, throughout the entire synthesis process of BFA, the composition and structure of CSMSs surfaces underwent changes, rendering further analysis to elucidate the mechanism of loading formation quite intriguing. XRD analysis was conducted on CSMCs to examine their crystal structure (Figure 1j). The XRD pattern of RF‐CSMSs showed weak yet broad X‐ray reflections near 21° and 43° 2*θ*. These reflections were associated with the (002) and (101) planes of amorphous carbon and crystalline/graphitic carbon, respectively (Figure S4, Supporting Information). After the addition of BFA, the intensity of the diffraction peak increased markedly, along with a shift in peak position from 2*θ* = 21° to 2*θ* = 26°. This shift was likely due to the successful loading of the clusters nanostructures. The XRD patterns of RF‐xBFA confirmed the existence of graphitic carbon, γ‐Fe_2_O_3_ and Fe^0^, with the XRD peaks exactly matching the Joint Committee on Powder Diffraction Standards (JCPDS) files (no. 41‐1487, no. 06‐0696 and no. 39‐1346), respectively.^[^ ^20^, ^29^ ^]^ Here, the surface of RF‐xBFA, which possesses semi‐embedded iron clusters nanostructures, consists of γ‐Fe_2_O_3_ and Fe^0^. Minimal changes were observed in the XRD spectra as the BFA/RF resin spheres mass ratio was adjusted, revealing that the composition of RF‐xBFA remained similar, with changes only in crystallinity. The crystallinity of RF‐xBFA increased with the BFA content until reaching a stable crystalline state. Notably, the crystallinity of iron clusters nanostructures of RF‐1.25BFA surface remained stable even after 540 days of air exposure. Thereby, it was confirmed that γ‐Fe_2_O_3_ and Fe^0^ were successfully loaded on the surface of RF‐xBFA during the synthesis process. Based on the known results, we hypothesize the processes that lead to the formation of metal and metal oxide nanoparticles within clusters. The addition of BFA initially leads to the formation and aggregation of tiny γ‐Fe_2_O_3_ nanoparticles. During the subsequent calcination process, the spheres were further carbonized. Concurrently, the carbon shell served as a reducing agent, transforming γ‐Fe_2_O_3_ to Fe^0^ under an inert gas environment, with Fe° forming the core within the γ‐Fe_2_O_3_ nanoparticles. This core‐shell structure formation mechanism was suggested to involve the inward diffusion of the Kirkendall effect.^[^ ^30^ ^]^ The carbon shell has consumed the oxygen element from the γ‐Fe_2_O_3_ nanoparticles at the contact points, leading to outward oxygen transfer, thus forming a reduced Fe° core in the middle or creating a void. As the temperature increases, the reduction processes continue, resulting in iron clusters nanostructures with a core‐shell structure. Transmission electron microscopy (TEM) images and selected‐area electron diffraction (SAED) patterns (Figure S5, Supporting Information) further confirmed the semi‐embedded core‐shell clusters nanostructures in RF‐xBFA. Since the two materials mainly differ in storage time, the diffraction characteristics of RF‐1.25BFA were primarily analyzed. A thorough observation of Figure S5b (Supporting Information) revealed that the crystalline texture of the iron clusters nanostructures exhibited core‐shell structural characteristics. Voids were observed between the Fe° core and the γ‐Fe_2_O_3_ shell of a certain thickness. The core‐shell clusters nanostructures were also observed in RF‐1.25BFA‐540 (Figure S5e, Supporting Information), demonstrating the structural stability of these clusters nanostructures. In Figure S5b (Supporting Information), the lattice distances of 0.3223 and 0.3251 nm between the characteristic planes of shell of clusters nanostructures were confirmed to belong to the (211) crystal plane of γ‐Fe_2_O_3_. Although the (211) crystal plane of γ‐Fe_2_O_3_ was not marked in Figure 1j due to the influence of graphitic carbon peak, but the aforementioned results also confirmed its existence. SAED patterns showed distinct circular diffraction ring and diffraction spots, suggesting the polycrystalline nature of RF‐1.25BFA and RF‐1.25BFA‐540, consistent with the XRD analysis, as seen in Figure S5c,f (Supporting Information). Thereby, this corroborated that iron clusters nanostructures were indeed the Fe^0^@γ‐Fe_2_O_3_ core‐shell clusters nanostructures.

To estimate the surface area and pore characteristics of adsorbents, the N_2_ adsorption–desorption isotherms of the CSMCs were investigated at 77 K (Figure S6, Supporting Information).^[^ ^31^ ^]^ Table S1 (Supporting Information) lists the detailed structural parameters of the CSMCs. The isotherm of RF‐CSMSs displays type‐I curves without a hysteresis loop, reflecting its solid characteristic and microporous structure. The micropores primarily originated from the evolution of gases from the organic polymers during carbonization.^[^ ^32^ ^]^ With increasing BFA content, the total specific surface area of RF‐xBFA decreased while the mesoporous surface area increased compared to those of RF‐CSMSs (Figure S6a, Supporting Information). RF‐xBFA exhibited a typical type‐IV isotherm with a sharp increase at low pressure (*P*/*P_0_* < 0.1), signifying the presence of micropores in the carbon spheres. There was also a noticeable rise at *P*/*P_0_* = 0.3–0.4. Meanwhile, a prominent H_4_ hysteresis loop appeared at *P*/*P_0_* = 0.45–0.9 in its isotherm, which is the typical characteristic of mesoporous derived from the out mesoporous carbon shell. The improved mesoporous characteristics were due to the generation of the core‐shell clusters nanostructures. The corresponding pore size distributions of the CSMCs, derived from the Barrett–Joyner–Halenda (BJH) model, clearly confirmed that (Figure S6b, Supporting Information) the pore sizes of RF‐xBFA were centered ≈3.9 nm. In contrast, the adsorption pore size distribution curve of RF‐CSMSs had no obvious peaks. Noticeably, the specific surface area and mesoporous surface area of RF‐1.25BFA‐540 decreased slightly with increased storage time, likely due to the minor collapse of the surface structure. Furthermore, Raman measurements were conducted to characterize the carbon structure of the CSMCs. As is evident from Figure 1k, Raman spectra presented the broad peaks at 1342 and 1587 cm^−1^, corresponding to the disordered structure of sp^2^‐hybridized carbon atoms (D band) and the E2 g vibration of sp^2^‐hybridized carbon atoms (G band), respectively.^[^ ^33^ ^]^ The intensity ratio of D‐band to G‐band (I_D_/I_G_) reflects the graphitization of carbon materials.^[^ ^34^ ^]^ The I_D_/I_G_ value of RF‐CSMSs (0.96) is lower than that of any RF‐xBFA, indicating the higher graphitization degree in RF‐CSMSs compared to RF‐xBFA. Furthermore, with the increase in BFA content, the I_D_/I_G_ ratio initially increased and then decreased, demonstrating an alternating trend. This suggested that the presence of iron clusters nanostructures introduced different levels of structural defects, potentially leading to systematic variations in the overall structural disorder or graphitization. The magnetic properties of RF‐xBFA were analyzed through vibrating‐sample magnetometer (VSM). Typical magnetization curves as a function of the applied field at room temperature (300 K) are shown in Figure 1l. The saturation magnetization of RF‐0.50BFA, RF‐0.75BFA, RF‐BFA, RF‐1.25BFA, RF‐1.25BFA‐540 and RF‐1.50BFA were measured to be 6.70, 7.39, 19.81, 63.29, 43.48 and 34.71 emu g^−1^, respectively, all exhibiting a coercivity below 100 Oe at room temperature. Notably, although the saturation magnetization of RF‐1.25BFA decreased slightly over time, it remained at a high level. As depicted in Figure 1m, RF‐1.25BFA and RF‐1.25BFA‐540 could be readily redispersed in the liquid phase, with the suspended materials easily recollected using a magnet within 30 s. This demonstrates their ability to be controlled by an external magnetic field.^[^ ^35^ ^]^ This characteristic is crucial for precisely placing adsorbents as needed in areas contaminated by heavy metals under the influence of an external magnetic field. Evidently, the saturation magnetization of RF‐xBFA was approximately proportional to the loading degree of iron clusters nanostructures until the spherical structure cracked and disintegrated, disrupting this trend. Besides, the thermal behavior of RF‐CSMSs, RF‐1.25BFA and RF‐1.25BFA‐540 was investigated using the thermogravimetric (TG) and differential thermogravimetry (DTG) under air and argon (Figure S7, Supporting Information). The weight‐loss peak of RF‐CSMSs, RF‐1.25BFA and RF‐1.25BFA‐540 occurred at ≈582, 494, and 478 °C due to the formation of aromatic hydrocarbons from the pyrolysis of heterocyclic compounds under air (Figure S7a,b, Supporting Information). The mass loss of RF‐CSMSs was ≈98% in the temperature range of 520–630 °C. Around 400 °C, the burning point of graphitized carbon was reached, leading to a rapid decrease in the mass of RF‐1.25BFA and RF‐1.25BFA‐540. Above 530 °C, the remaining weights of RF‐1.25BFA and RF‐1.25BFA‐540 were ≈37% and 33%, respectively, corresponding to the formation of a stable iron oxide phase. There was a positive correlation between total mass loss at 500–650 °C and γ‐Fe_2_O_3_ proportion (Figure S7c,d, Supporting Information), which may indicate the transformation of γ‐Fe_2_O_3_ and reduction to Fe^0^ in RF‐1.25BFA and RF‐1.25BFA‐540.^[^ ^36^ ^]^

### 2.2 Adsorption Performance

**Figure 2:**
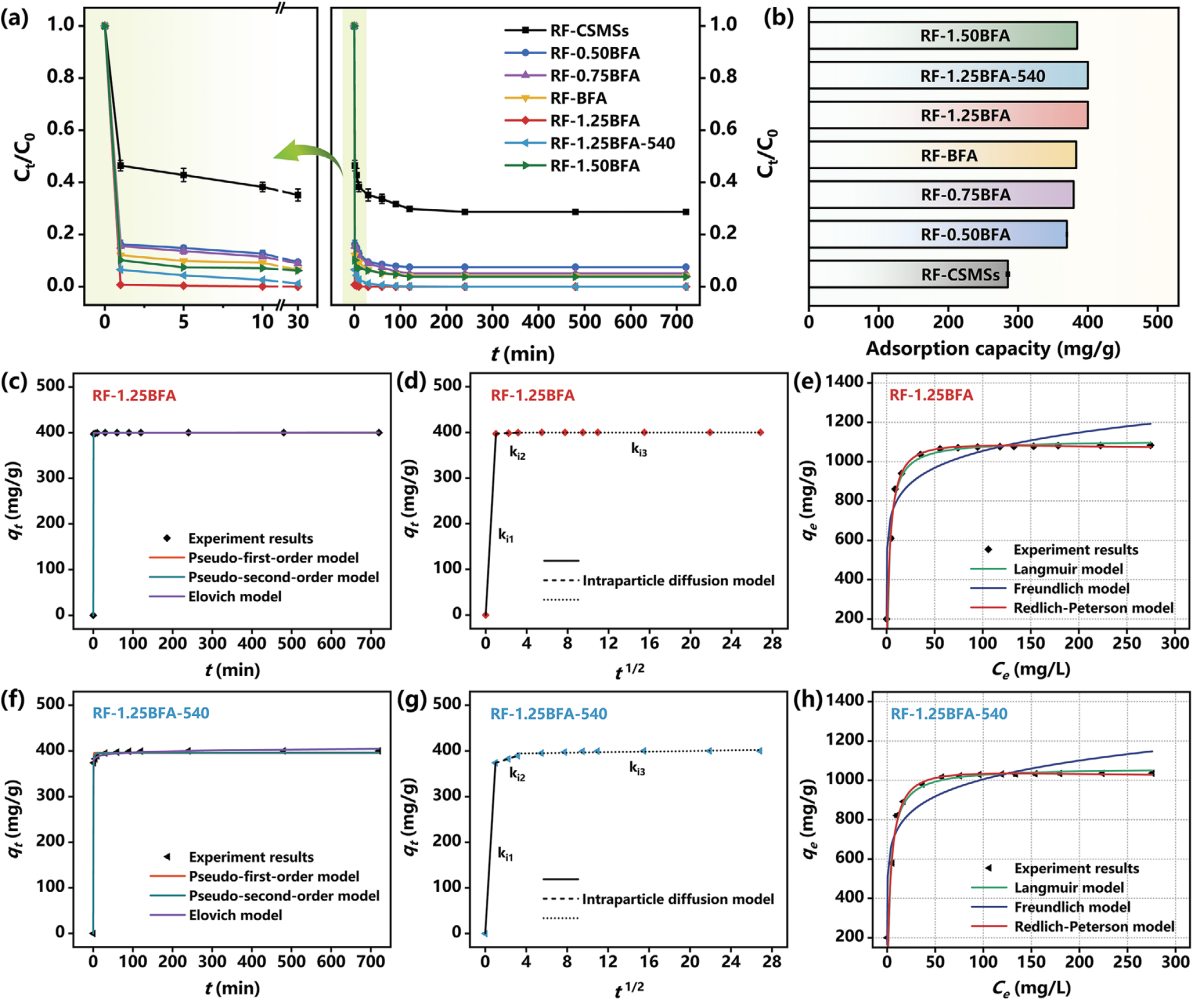
a) Adsorption performance of the CSMCs for Cd(II). b) Adsorption capacity of the CSMCs. Adsorption kinetics for Cd(II) adsorption on c,d) RF‐1.25BFA and f,g) RF‐1.25BFA‐540: the pseudo‐first‐order, pseudo‐second‐order, Elovich equation and intraparticle diffusion model fitting curves. Conditions: [Initial Cd(II)] = 10 mg L^−1^, adsorbent dosage = 0.025 g L^−1^, T = 25 °C, pH = 6.50 and equilibrium time = 720 min. Adsorption isotherms for Cd(II) adsorption on e) RF‐1.25BFA and h) RF‐1.25BFA‐540: Langmuir, Freundlich, and Redlich–Peterson model fitting curves. Conditions: [Initial Cd(II)] = 5–300 mg L^−1^, adsorbent dosage = 0.025 g L^−1^, T = 25 °C, pH = 6.50 and equilibrium time = 720 min.

Given the importance of the ultrafast removal of ultra‐low concentration of Cd(II) from mine drainage, a concentration of 10 mg L^−1^ was used as the primary study concentration. Further, the adsorbent dosage is essential for determining its effectiveness and directly determines the economic impact of the adsorption process. Considering the comparatively high adsorption capacity of RF‐1.25BFA at a dosage of 0.025 g L^−1^, this optimized adsorbent dosage was employed for all further experiments to avoid wasting adsorption sites and space (Figure S8, Supporting Information). Excessive amounts of adsorbent reduce the adsorption capacity because of a higher proportion of unoccupied binding sites. Another reason for decreased adsorption capacity is the aggregation of the adsorbents at high concentrations, which can reduce the surface areas of the adsorbents. This tendency implies that the degree of adsorption depends on the relative availability of binding sites.^[^ ^37^ ^]^ To determine the adsorption equilibrium time, the effect of contact time on Cd(II) adsorption was studied, as illustrated in **Figure** **2**a. It was noteworthy that the instantaneous adsorption rate of Cd(II) on RF‐1.25BFA and RF‐1.25BFA‐540 was very rapid within the first 1 min. The adsorption efficiency of RF‐1.25BFA could exceeding 99.8% within 10 min, and equilibrium was nearly achieved in just 30 min, which is an astonishing result. With the further increases in contact time, the Cd(II) adsorption on CSMCs reached complete equilibrium at 720 min. Therefore, a contact time of 720 min was considered appropriate for equilibrium adsorption of Cd(II) in all subsequent experiments. At equilibrium, the Cd(II) adsorption capacity of RF‐1.25BFA and RF‐1.25BFA‐540 reached 400.00 mg g^−1^, bringing the Cd(II) concentration below the detection limit of the instrument, effectively to zero. Consequently, the adsorption capacity order for Cd(II) was RF‐1.25BFA (400.00 mg g^−1^) = RF‐1.25BFA‐540 (400.00 mg g^−1^) > RF‐1.50BFA (384.72 mg g^−1^) > RF‐BFA (383.24 mg g^−1^) > RF‐0.75BFA (379.60 mg g^−1^) > RF‐0.50BFA (369.88 mg g^−1^) > RF‐CSMSs (285.20 mg g^−1^) (Figure 2b). It is worthwhile mentioning that the adsorption capacity of RF‐1.25BFA and RF‐1.25BFA‐540, despite having relatively small S_BET_, was larger than that of RF‐CSMSs, indicating that the specific surface area was not the pivotal factor in Cd(II) adsorption.^[^ ^38^ ^]^ In view of the importance of the adsorption efficiency and adsorption capacity, RF‐1.25BFA and RF‐1.25BFA‐540, with their remarkable adsorption performance, were used as the main analysis materials in the following study. The measured data can be utilized for evaluating the adsorption kinetics of Cd(II).

#### 2.2.1 Adsorption Kinetics

The adsorption rate is pivotal in the practical application and design of adsorption materials. Understanding adsorption kinetic parameters is vital for optimizing both the design and operation of adsorption units. The adsorption kinetic data were interpreted through the pseudo‐first‐order model,^[^ ^39^ ^]^ pseudo‐second‐order model,^[^ ^40^ ^]^ Elovich model^[^ ^41^ ^]^ and intraparticle diffusion model.^[^ ^42^ ^]^ All relevant equations and parameters are detailed in Text S4 and S5 and Table S2 (Supporting Information). The kinetic curves for Cd(II) adsorption on CSMCs are shown in Figure 2c,f and Figure S9 (Supporting Information). Preliminary investigations indicated that RF‐xBFA absorbed Cd(II) faster than RF‐CSMSs, with RF‐1.25BFA and RF‐1.25BFA‐540 showing the more prominent adsorption performances. The adsorption reaction for Cd(II) on RF‐1.25BFA and RF‐1.25BFA‐540 occurred rapidly within the first 1 min, likely due to abundant active load sites and a favorable high‐alkalinity adsorption microenvironment constructed by the iron clusters nanostructures of RF‐1.25BFA and RF‐1.25BFA‐540. Equilibrium for Cd(II) adsorption on RF‐1.25BFA and RF‐1.25BFA‐540 was achieved in roughly 30 min. Based on the estimated correlation coefficient (*R* ^2^) in Table S2 (Supporting Information), the pseudo‐second‐order kinetic model provided a slightly better fit to the data than the pseudo‐first‐order kinetic model. This finding manifested that the rate‐controlling step was not due to boundary layer resistance.^[^ ^43^ ^]^ The pseudo‐second‐order kinetic model was chosen primary because it posits that the rate‐limiting step could involve different chemisorption mechanisms for RF‐CSMSs and RF‐xBFA, which include electron sharing or exchange between the absorbent and absorbate.^[^ ^44^ ^]^ Remarkably, the Elovich equation, suitable for heterogeneous adsorption processes primarily driven by chemical adsorption, also matched the adsorption performances of CSMCs.^[^ ^45^ ^]^ This model successfully described the adsorption kinetics of Cd(II) on CSMCs, demonstrating that the adsorption of Cd(II) by RF‐CSMSs and RF‐xBFA was primarily governed by surface chemistry, with chemical adsorption as the potential rate‐controlling step.^[^ ^46^ ^]^

An intraparticle diffusion model was used to further assess the adsorption process, exploring the control steps, inferring reaction types and predicting rate‐determining steps.^[^ ^47^ ^]^ The equation provided by Weber and Morris^[^ ^48^ ^]^ is expressed in Text S5 and Equation S9 (Supporting Information). As per this model, when intraparticle diffusion is the rate‐limiting step in the adsorption process, the *q_t_* versus *t* ^1/2^ plot will produce a straight line intersecting the origin. Figure 2d,g and Figure S10 (Supporting Information) present that the plots of *q_t_* to *t* ^1/2^ exhibit a piecewise‐linear pattern with three slopes (*k*_*i*1_, *k*_*i*2_ and *k*_*i*3_). The values of *k_i_* and *C*, derived from the slope and intercept of *q_t_* versus *t* ^1/2^, are listed in Text S5 and Table S2 (Supporting Information). The results manifested that the non‐zero linear graph of three stages adsorption process which designated both the rationality and the participation of intraparticle diffusion processes.^[^ ^49^ ^]^ As demonstrated in Figure 2d,g, the initial stage with a steep slope corresponded to the instantaneous diffusion stage or external surface adsorption (*k*_*i*1_, from 0 to 1 min), where ≈99.3% and 93.5% of Cd(II) were quickly adsorbed onto the exterior surface of RF‐1.25BFA and RF‐1.25BFA‐540, respectively.^[^ ^50^ ^]^ This interesting phenomenon confirmed that almost all the adsorption for Cd(II) was completed in the first stage. As the adsorption time continues, the available active sites in the surface adsorption microenvironment of RF‐1.25BFA and RF‐1.25BFA‐540 became entirely occupied, prompting Cd(II) to migrate into the pores of the adsorbent, where it was adsorbed by the interior surfaces of the mesopores and micropores.^[^ ^51^ ^]^ As Cd(II) penetrated deeper, the diffusion resistance rose, resulting in a reduced diffusion rate (*k*_*i*2_).^[^ ^52^ ^]^ It can be interpreted by the fact that intraparticle diffusion into the mesopores and micropores was the rate‐limiting step. It must also be mentioned that the second stage rate of RF‐1.25BFA‐540 was larger than that of RF‐1.25BFA. A plausible explanation is that the mean pore and mesoporous sizes of RF‐1.25BFA‐540 increased due to micro‐cracks on the surface after 540 days of storage, making the surface microenvironment RF‐1.25BFA‐540 slightly inferior to that of RF‐1.25BFA. The third stage was the adsorption saturation stage, during which the intraparticle diffusion rate progressively slowed and reached equilibrium, attributed to the low residual concentration of Cd(II) in the solution (*k*_*i*3_).^[^ ^53^ ^]^ Since the curve of the second stage did not intersect the origin, it suggests that intraparticle diffusion was not the sole rate‐limiting step; other factors, including prompt chemical reactions might have also contributed to the removal of Cd(II). Figure S10 (Supporting Information) also shows the intraparticle diffusion model fitting curve for Cd(II) adsorption on other materials.

#### 2.2.2 Adsorption Thermodynamic

The study of Cd(II) adsorption mechanisms on RF‐1.25BFA and RF‐1.25BFA‐540 focused on adsorption isotherms and thermodynamic properties. The adsorption isotherms were utilized to illustrate the interactions between equilibrium adsorption amount and concentration at a particular temperature, analyzing the adsorption interfacial phenomenon.^[^ ^54^ ^]^ Four isotherm equations were tested in this study: Langmuir model,^[^ ^55^ ^]^ Freundlich model,^[^ ^56^ ^]^ Redlich–Peterson model^[^ ^57^ ^]^ and Dubinin–Radushkevich (D–R) model (Text S6).^[^ ^58^ ^]^ The parameters of adsorption isotherm models are summarized in Table S3 (Supporting Information). The Langmuir model is appropriate for monolayer sorption system,^[^ ^59^ ^]^ while the Freundlich model, an empirical equation, describes heterogeneous systems.^[^ ^60^ ^]^ The Redlich–Peterson isotherm model incorporates elements from both the Langmuir and Freundlich equations, suggesting a hybrid adsorption mechanism that diverges from ideal monolayer adsorption.^[^ ^61^ ^]^ As illustrated in Figure 2e,h, the Cd(II) adsorption processes on RF‐1.25BFA and RF‐1.25BFA‐540 were fitted using three nonlinear equations. As the concentration increased, the adsorption capacity also increased and eventually became saturated. These results demonstrated that the Redlich–Peterson model provided a more accurate simulation of the adsorption experiments than the Langmuir and Freundlich model (Table S3, Supporting Information). From the above, it is reasonable to conclude that the adsorption process was a hybrid one. The theoretical maximum adsorption capacities of RF‐1.25BFA and RF‐1.25BFA‐540 were 1108.87 and 1065.06 mg g^−1^ for Cd(II), respectively. The adsorption capacity of RF‐1.25BFA reached a record level and remained extremely high even after 540 days of storage. As such, the superb adsorption performances of RF‐1.25BFA could be attributed to the favorable high‐alkalinity adsorption microenvironment constructed by iron clusters nanostructures, which facilitated the ultrafast two‐step enrichment–hydrolysis adsorption process of Cd(II). The theoretical maximum adsorption capacity for Cd(II) on RF‐1.25BFA was much greater than many literature reports, including biochar, graphene, carbon nanotubes, metal oxide and their modified materials that have been extensively studied over the past few decades (Table S4, Supporting Information). The D–R isotherm model helps identify whether the nature of the adsorption process is physical or chemical. The linear equation of the D–R isotherm^[^ ^62^ ^]^ is provided in Text S6 and Equation S13 (Supporting Information). The D–R isotherm model fit the equilibrium data well, with *R* ^2^ values of 0.9989 and 0.9954 for Cd(II) adsorption on RF‐1.25BFA and RF‐1.25BFA‐540, respectively (Figure S11 and Table S3, Supporting Information). The *E* (kJ mol^−1^) value can reveal whether the adsorption mechanism is physical or chemical in nature.^[^ ^63^ ^]^ The mean free energy of adsorption for Cd(II) adsorption on RF‐1.25BFA and RF‐1.25BFA‐540 were 14.43 and 13.98 kJ mol^−1^, respectively, which fall within the typical energy range for chemisorption bonding energy, i.e., 8–16 kJ mol^−1^.^[^ ^64^ ^]^

The thermodynamic quantities were selected to study the adsorption process and type. Cd(II) adsorption performance and kinetic curves of RF‐1.25BFA and RF‐1.25BFA‐540 at different temperature were depicted in Figures S12, S13 and Table S5 (Supporting Information). When the adsorption temperature was raised from 5 to 35 °C, the adsorption capacities of RF‐1.25BFA were 394.48, 397.64, 400.00, and 400.00 mg g^−1^, respectively; the adsorption capacities of RF‐1.25BFA‐540 were 392.32, 396.28, 400.00, and 400.00 mg g^−1^, respectively. With respect to practical applications, RF‐1.25BFA and RF‐1.25BFA‐540 still exhibited very high adsorption capacity under extreme environmental conditions (e.g., T = 5 °C), which is the premise of large‐scale application of adsorbents. Apparently, the increase in adsorption capacity with increasing temperature demonstrated that the adsorption process was endothermic. Thermodynamic calculations were conducted to understand the impact of temperature on Cd(II) adsorption, further explaining the endothermic nature of the process and determining its spontaneity. The thermodynamic parameters, such as the Gibbs free energy change (Δ*G* ^θ^), standard enthalpy change (Δ*H* ^θ^) and standard entropy change (Δ*S* ^θ^), are given by Text S6 and Equations S15 and S16 (Supporting Information). The Δ*G* ^θ^ was calculated to be −9.87, −12.28, −28.52, and −29.48 kJ mol^−1^ for 5, 15, 25, and 35 °C, respectively, indicating that Cd(II) adsorption on RF‐1.25BFA was thermodynamically favored. The Δ*G* ^θ^ was calculated to be −9.09, −11.18, −28.52, and −29.48 kJ mol^−1^ for 5, 15, 25, and 35 °C, respectively, elucidating that Cd(II) adsorption on RF‐1.25BFA‐540 was also thermodynamically favored. The estimated thermodynamic parameters (Table S6, Supporting Information) showed negative Δ*G* ^θ^, positive Δ*H* ^θ^, and positive Δ*S* ^θ^, suggesting a spontaneous and endothermic sorption reaction between 5 and 35 °C, with an increased degree of randomness at the solid–liquid interface during Cd(II) adsorption.^[^ ^65^ ^]^ Increasing the temperature promoted the adsorption of Cd(II). Specifically, the positive Δ*H* ^θ^ indicated that the adsorption process was endothermic,^[^ ^66^ ^]^ and the positive Δ*S* ^θ^ reflected an affinity of RF‐1.25BFA and RF‐1.25BFA‐540 toward Cd(II) in aqueous solutions, possibly implying some structural changes in the adsorbents.^[^ ^67^ ^]^ Typically, as the temperature increased, Δ*G* ^θ^ became more negative (with its absolute value increasing) at the same adsorbate loading, suggesting that the adsorption driving force increased with temperature. This observation aligns with the increase in adsorption capacity with rising temperature.^[^ ^68^ ^]^ These results agree well with those obtained from the D–R isotherm model.

#### 2.2.3 Effect of Key Factors on the Removal of Cd(II)

**Figure 3:**
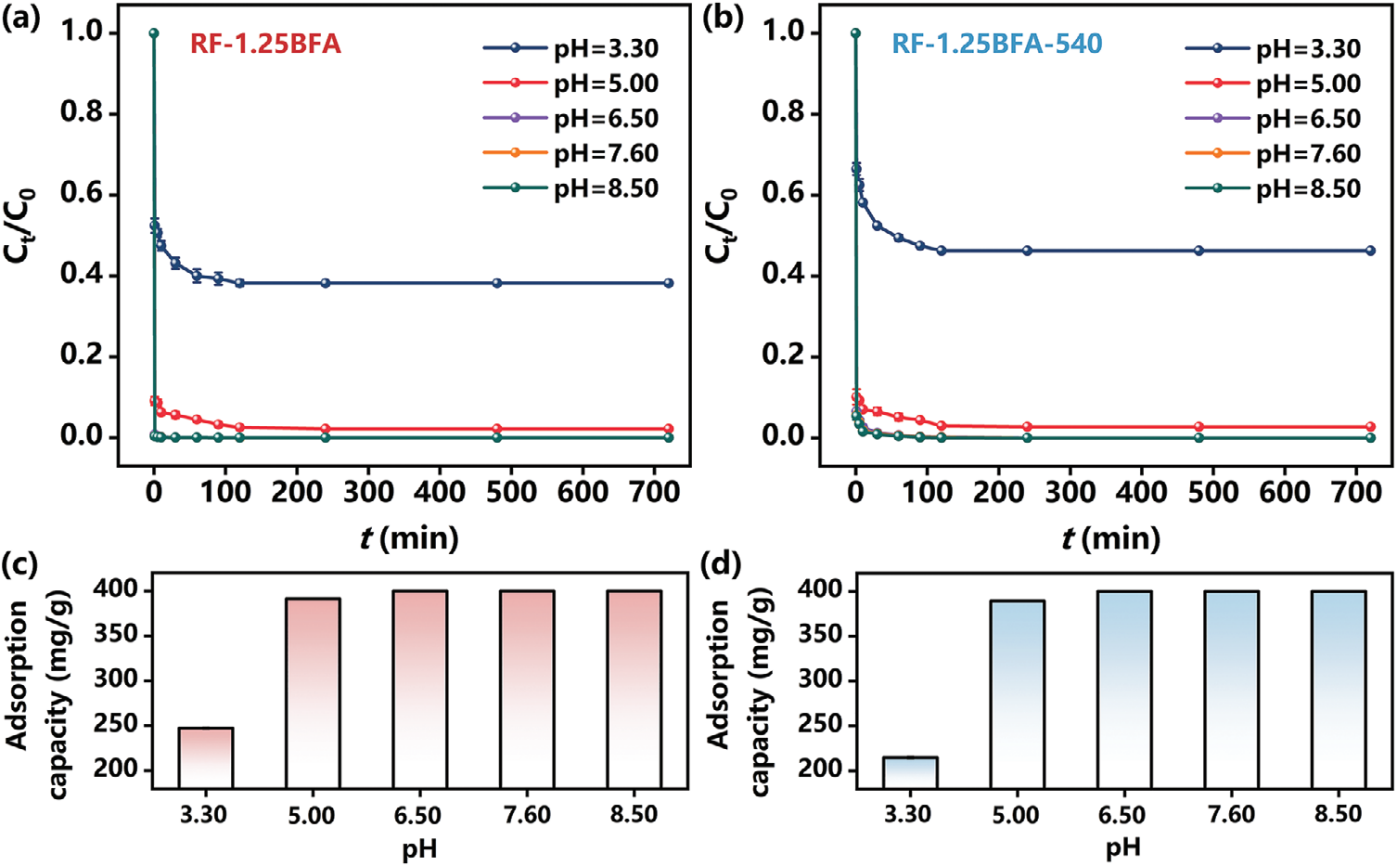
Influence of pH toward adsorption performance of a) RF‐1.25BFA and b) RF‐1.25BFA‐540 for Cd(II). Adsorption capacity of c) RF‐1.25BFA and d) RF‐1.25BFA‐540 at different pH conditions. Conditions: [Initial Cd(II)] = 10 mg L^−1^, adsorbent dosage = 0.025 g L^−1^, T = 25 °C, pH = 3.30, 5.00, 6.50, 7.60, 8.50 and equilibrium time = 720 min.

Solution pH was recognized as the most important factor that significantly affects heavy metals adsorption onto adsorbents.^[^ ^69^ ^]^ The initial solution pH influences both the protonation of the functional groups of adsorbents and the ionization degree and speciation of adsorbates in the liquid phase, thereby affecting the interactions between adsorbents and adsorbates.^[^ ^70^ ^]^ Cd(II) readily forms precipitates of metal hydroxides in the liquid phase under elevated pH levels. Hence, the experimental pH for this study was deliberately controlled below a designated threshold, specifically maintaining it below 8.9. The influence of initial solution pH, ranging from 3.30 to 8.50, on the adsorption performance and adsorption capacity for Cd(II) adsorption by RF‐1.25BFA and RF‐1.25BFA‐540 was investigated, as shown in **Figure** **3**. As the pH was increased, the protonation reactions weakened, and Cd(II) became rapidly enriched in the high‐alkalinity adsorption microenvironment constructed by iron clusters nanostructures and hydrolyzed with a large number of hydroxyl groups of carbon spheres surface, resulting in a rapid decrease in its concentration (Figure 3a,b). The findings also revealed that the adsorption capacity for Cd(II) adsorption on RF‐1.25BFA and RF‐1.25BFA‐540 could still reach 247.28 and 214.96 mg g^−1^, respectively, at pH 3.30 (Figure 3c,d), highlighting the significance of the high‐alkalinity adsorption microenvironment in the Cd(II) adsorption process. More importantly, the adsorption capacity for Cd(II) gradually approached higher levels of 391.36 and 389.08 mg g^−1^ at a solution pH of 5.0. The kinetic curves for Cd(II) adsorption on RF‐1.25BFA and RF‐1.25BFA‐540 under different pH conditions are presented in Figure S14 (Supporting Information), with all corresponding kinetic parameters provided in Table S7 (Supporting Information). In the high pH region, specifically in weak alkaline environment, the adsorption of RF‐1.25BFA and RF‐1.25BFA‐540 for Cd(II) had a higher adsorption rate at the initial stage. Plus, the sorption affinity of RF‐1.25BFA and RF‐1.25BFA‐540 for Cd(II) was closely linked to their surface charge, which is affected by the pH of the aqueous solution. Zeta potential analysis quantifies the magnitude and effective surface charge density associated with the double layer around the particles. The zeta potentials of RF‐1.25BFA and RF‐1.25BFA‐540 at different pH values (3.30–8.50) are depicted in Figure S15 (Supporting Information). The results indicated that the zeta potentials of both materials decreased as pH increased, which can be attributed to the deprotonation of functional groups. The pH_PZC_ (point of zero charge) values of RF‐1.25BFA and RF‐1.25BFA‐540 were ≈4.68 and 4.80, respectively. Below their respective pH_PZC_ values, the materials exhibited a positively charged surface potential resulting from protonation by H^+^ ions. In the lower pH region, the overall charges on RF‐1.25BFA and RF‐1.25BFA‐540 surfaces were positive, and H^+^ ions competed strongly with Cd(II) for available active sites. However, as the pH exceeded their pH_PZC_ values, the surfaces of RF‐1.25BFA and RF‐1.25BFA‐540 became electronegative due to the presence of negatively charged hydroxyl groups on their surfaces. Compared with RF‐1.25BFA, RF‐1.25BFA‐540 had a reduced number of hydroxyl groups on its surface, and these oxygen‐containing functional groups exhibited negative charges. Hence, there were slight differences in the zeta potential on their surfaces. The possible mechanism suggests that in a strong acidic environment, H^+^ ions could partially disrupt the high‐alkalinity adsorption microenvironment constructed by iron clusters nanostructures. Consequently, only a portion of Cd(II) would undergo hydrolysis, while H^+^ ions would also preferentially occupy the available active sites. As the pH increased, the surface alkalinity characteristics of RF‐1.25BFA and RF‐1.25BFA‐540 facilitated the formation of the stable microenvironment that promoted Cd(II) adsorption. Simultaneously, the concentration of H^+^ ions decreased with increasing pH, significantly reducing their competition with Cd(II) for available active sites. Moreover, the high‐alkalinity adsorption microenvironment was less susceptible to disturbance by H^+^ ions in the liquid phase, and any interference from trace amounts of H^+^ ions was negligible. Therefore, the adsorption capacity of Cd(II) by RF‐1.25BFA and RF‐1.25BFA‐540s was considerable across a wide pH range, including lower pH environment. More explicitly, the remarkable adsorption performance of Cd(II) on the surface of RF‐1.25BFA and RF‐1.25BFA‐540 occurred primarily through non‐electrostatic interactions.^[^ ^71^ ^]^

The mine drainage usually contained a large number various cations and anions which could negatively or positively influence the adsorption of Cd(II). When two or more metal ions coexist in solution, they can either enhance (synergism), reduce (antagonism), or have no impact (non‐interaction) on the system's metal ion adsorption capacity. Some heavy metal ions like Zn^2+^, Cu^2+^, Al^3+^, Pb^2+^, Fe^3+^, Mn^2+^ and Co^2+^ often co‐exist with Cd(II) in mine drainage, stemming from industrial applications and mining operations. Also, alkali and alkaline‐earth metal cations such as Na^+^, K^+^, Ca^2+^ and Mg^2+^ were commonly present with metal contaminants at contaminated sites. Thus, it was imperative to examine the competitive influences of these metal ions with Cd(II) during adsorption onto RF‐1.25BFA and RF‐1.25BFA‐540. To be specific, the Cd(II) adsorption on RF‐1.25BFA and RF‐1.25BFA‐540 had almost no significant effect when the concentration of competitive cations was half that of Cd(II) (Figure S16a,b, Supporting Information). When the concentrations of Cd(II) and competitive cations were equal, the adsorption efficiency of Cd(II) was still greater than 70%. Simultaneously, RF‐1.25BFA and RF‐1.25BFA‐540 also showed excellent adsorption performance for certain competitive cations (Zn^2+^, Cu^2+^, Al^3+^, Pb^2+^, Fe^3+^, Mn^2+^) in the presence of Cd(II), as depicted in Figure S17 (Supporting Information). This can be explained by two factors: direct competition among cations for active load sites in the high‐alkalinity adsorption microenvironment and the surface charge shielding effect induced by competitive cations.^[^ ^72^ ^]^ Collectively, the characteristics of heavy metal ions, including electronegativity, hydrolysis ability, and their preference for specific active adsorption sites, influenced the strength of hydroxyl coordination process of metal ions in the high‐alkalinity adsorption microenvironment.^[^ ^73^ ^]^ These above‐mentioned results indicated that under the conditions of this experiment, competitive cations did not exert a significant adverse or inhibitory effect on adsorption. With respect to practical applications, RF‐1.25BFA was expected to be used as the more universal adsorbent for the removal of various heavy metal ions due to its lack of specificity toward certain heavy metal ions. The effects of common anions at several different concentrations (5 and 10 mg L^−1^) on the adsorption performance of RF‐1.25BFA and RF‐1.25BFA‐540 for Cd(II) are illustrated in Figure S16c,d (Supporting Information). The results indicated that the inhibitory effect of NO− 3, Cl^−^, SO2− 4, HSO− 4, PO3− 4, CO2− 3 and HCO− 3 on Cd(II) adsorption was almost negligible. This could be attributed to their electronegativity in solution, while RF‐1.25BFA and RF‐1.25BFA‐540 remained electronegative at pH > pH_PZC_. Due to electrostatic repulsion effects, competitive anions did not compete effectively for adsorption sites, thus not inhibiting the removal of Cd(II) by RF‐1.25BFA and RF‐1.25BFA‐540. The impact of ionic strength on Cd(II) adsorption by RF‐1.25BFA and RF‐1.25BFA‐540 was also assessed to estimate their efficiency in mine drainage. Specifically, the study investigated effects of six different initial concentrations (0, 0.001, 0.01, 0.02, 0.05, and 0.1 M) of background electrolyte (NaNO_3_ and NaCl) on Cd(II) adsorption of RF‐1.25BFA and RF‐1.25BFA‐540. As the concentrations of NaNO_3_ and NaCl increased from 0 to 0.1 M, NaCl exerted a more pronounced influence on the Cd(II) adsorption (Figure S18, Supporting Information). This was likely due to the presence of Cl^−^ ions in the adsorption system. Previous studies have demonstrated that the presence of Cl^−^ ions reduces Cd(II) adsorption by various adsorbents through the formation of Cd–Cl complexes, particularly CdCl^+^, which exhibited reduced sorption affinity relative to Cd(II).^[^ ^74^ ^]^

### 2.3 A Possible New Mechanism for Cd(II) Adsorption on RF‐xBFA

**Figure 4:**
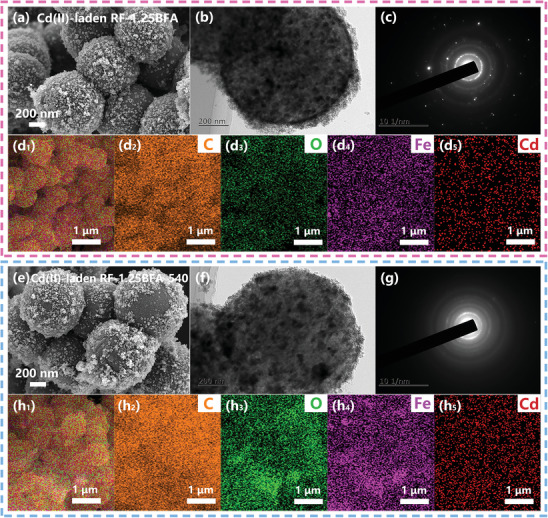
FE‐SEM images and EDS elemental mappings of a,d_1_–d_5_) RF‐1.25BFA and e,h_1_–h_5_) RF‐1.25BFA‐540 after Cd(II) adsorption. TEM images and SAED patterns of b,c) RF‐1.25BFA and f,g) RF‐1.25BFA‐540 after Cd(II) adsorption.

Based on the aforementioned results, the adsorption mechanism of RF‐xBFA for Cd(II) might not be limited to the generalized mechanism involving electrostatic interaction, Van der Waals interaction, surface complexation interaction, precipitation process, hydrophobic interaction, Lewis acid–base interaction, cation‐π interactions, hydrogen bonding, ion exchange, and reduction/oxidation, among others.^[^ ^75^ ^]^ These differences were closely linked to the physicochemical properties of RF‐xBFA. Depending on the standard potential E^0^ of heavy metals, the removal mechanisms could be either adsorption, reduction/precipitation, or both. For instance, with an E^0^ standard potential of −0.41 eV for Fe^0^, heavy metals with an equal or more negative potential, e.g., E_Cd(II)/Cd(0)_^0^ = −0.40 eV, primarily undergo adsorption rather than reduction in our study.^[^ ^76^ ^]^ Thus, the removal mechanism of Cd(II) was based on adsorption in our work. The micromorphology of RF‐1.25BFA and RF‐1.25BFA‐540 after Cd(II) adsorption was examined using SEM images, EDS elemental mappings and TEM images (**Figure** **4**). Significant microstructural changes were observed on the surface of RF‐1.25BFA and RF‐1.25BFA‐540, likely due to Cd(II) adsorption and the development of amorphous compound Cd(OH)_2_ around iron clusters nanostructures (Figure 4c,g). Additionally, the XRD patterns indicated that the new compounds (e.g., γ‐FeOOH and Cd(OH)_2_) on the surface exhibited amorphous structure or very weak crystalline structures (Figure S19, Supporting Information). The surface of RF‐1.25BFA (Figure 4a,b) and RF‐1.25BFA‐540 (Figure 4e,f) were covered with numerous densely packed fine particles with a frosting morphology. In Figure 4d_1_–d_5_,h_1_–h_5_, EDS elemental mappings of RF‐1.25BFA and RF‐1.25BFA‐540 showed uniformly distributed C, O, Fe and Cd elements. In contrast, SEM images, EDS elemental mappings and XRD patterns of RF‐CSMSs after Cd(II) adsorption can be seen in Figures S20 and S21 (Supporting Information). Although Cd element was uniformly distributed on the surface, there were no significant changes observed in morphology or XRD pattern. Moreover, as a control group, Cd(II) solution was replaced with an equal amount of deionized water. The characterization results of RF‐1.25BFA (Figure S22a, Supporting Information), RF‐1.25BFA‐540 (Figure S22b, Supporting Information) and RF‐CSMSs (Figure S22c, Supporting Information) after undergoing the same adsorption process in the liquid phase without Cd(II) confirmed that a significant amount of amorphous Cd(OH)_2_ compound had formed on the surface of RF‐1.25BFA and RF‐1.25BFA‐540 upon exposure to Cd(II) solution.

**Figure 5:**
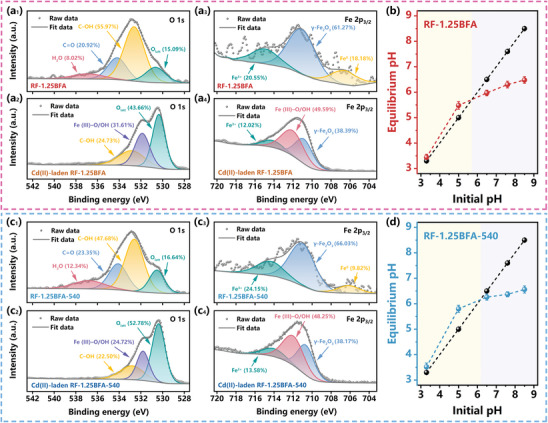
The high‐resolution XPS spectra of a_1_–a_2_,c_1_–c_2_) O 1 s and a_3_–a_4_,c_3_–c_4_) Fe 2 p_3/2_ in RF‐1.25BFA, Cd(II)‐laden RF‐1.25BFA, RF‐1.25BFA‐540 and Cd(II)‐laden RF‐1.25BFA‐540. The pH values change of the Cd(II) adsorption by b) RF‐1.25BFA and d) RF‐1.25BFA‐540 after equilibrium at different pH conditions (pH = 3.30, 5.00, 6.50, 7.60, 8.50).

In this study, the C 1 s, O 1 s, Fe 2 p_3/2_, Cd 3 d_5/2_ spectrum of CSMCs and Cd(II)‐laden CSMCs were characterized using XPS spectra to greater elucidate the adsorption mechanism of Cd(II) on RF‐xBFA, specifically focusing on RF‐1.25BFA and RF‐1.25BFA‐540. The O 1s spectra of RF‐1.25BFA (**Figure** **5**a_1_–a_2_), RF‐1.25BFA‐540 (Figure 5c_1_–c_2_) and RF‐CSMSs (Figure S23, Supporting Information) before and after Cd(II) adsorption aimed to clarify the role of hydroxyl groups in the hydrolysis process and the transformation of Fe^0^, γ‐Fe_2_O_3_ and γ‐FeOOH, and it was fitted with four components and three components corresponding to before and after adsorption. The binding energies of the components corresponded to O_latt_ (530.7 eV), C─OH (532.6 eV), C═O (534.2 eV) and adsorbed water (536.9 eV) for RF‐1.25BFA; O_latt_ (530.4 eV), Fe(III)─O/OH (531.9 eV) and C─OH (532.8 eV) for Cd(II)‐laden RF‐1.25BFA; O_latt_ (530.5 eV), C─OH (532.6 eV), C═O (534.1 eV) and adsorbed water (536.8 eV) for RF‐1.25BFA‐540; O_latt_ (530.4 eV), Fe(III)─O/OH (531.8 eV) and C─OH (532.8 eV) for Cd(II)‐laden RF‐1.25BFA‐540; O_latt_ (530.8 eV), C─OH (533.1 eV), C═O (534.6 eV) and adsorbed water (536.8 eV) for RF‐CSMSs; O_latt_ (531.1 eV), C─OH (532.5 eV) and C─O─Cd (533.5 eV) for Cd(II)‐laden RF‐CSMSs.^[^ ^77^ ^]^ Overall, the XPS spectra revealed extremely rich hydroxyl groups on the surface of RF‐CSMSs, RF‐1.25BFA and RF‐1.25BFA‐540, indicating their significant involvement in the adsorption processes, particularly pronounced in RF‐CSMSs (Figure S23a, Supporting Information). Generally speaking. the hydroxyl groups exhibit high hydrophilicity, but their hydrophobicity increases gradually with an increase in their number. This phenomenon occurs because the hydrogen bonds of the hydroxyl groups can interact with water molecules, enhancing their hydrophilic nature. However, when the number of hydroxyl groups reaches a certain threshold on the molecular surface, they can form interactions that create hydrophobic regions, thereby weakening overall hydrophilicity. Our experimental findings also support this observation (Figures S24 and S25, Supporting Information).^[^ ^78^ ^]^ After the adsorption of Cd(II) on RF‐1.25BFA and RF‐1.25BFA‐540, there was a significant decrease in the oxygen associated with C─OH groups, indicating that a majority of the hydroxyl groups on the carbon sphere surfaces were engaged in the hydrolysis process of Cd(II). In contrast, Cd(II) on RF‐CSMSs predominantly interacted via traditional complexation adsorption without external factor induction (Equations 1 and 2; Figure S23b, Supporting Information),^[^ ^79^ ^]^ with a small fraction possibly converting into weakly hydrolyzed Cd(OH)^+^ product (Equation 3; Figure S26a, Supporting Information). The iron clusters nanostructures present on the surface of RF‐1.25BFA and RF‐1.25BFA‐540 provided a relatively abundant source of lattice oxygen and Fe(III)–O/OH, the proportion of which increased with the adsorption of Cd(II). This observation further supported the conversion processes involving Fe^0^, γ‐Fe_2_O_3_ and γ‐FeOOH, as described in Equations (4), (5), (6), (7), (8), (9), (10).^[^ ^80^ ^]^ However, after Cd(II) adsorption, the O_latt_ peak generally shifted toward lower binding energies, which could be attributed to different oxidation states after adsorption or the formation of other compounds.^[^ ^77c^ ^]^ These corresponding changes were also evident from XRD characterization (Figure S19, Supporting Information). The presence of adsorbed water diminished upon Cd(II) adsorption, suggesting that the thickness of newly formed compounds exceeded the maximum detectable limit of the XPS technique, likely due to the formation of numerous frosting nanostructures. The Fe 2 p_3/2_ spectra before and after adsorption for Cd(II) on RF‐1.25BFA and RF‐1.25BFA‐540 were presented in Figure 5a_3_–a_4_,c_3_–c_4_, and it was also divided into three typical peaks: Fe^0^, γ‐Fe_2_O_3_ and Fe^3+^ before Cd(II) adsorption; γ‐Fe_2_O_3_, Fe(III)–O/OH and Fe^3+^ after Cd(II) adsorption.^[^ ^81^ ^]^ The binding energies of the components corresponded to Fe^0^ (706.6 eV), γ‐Fe_2_O_3_ (711.1 eV) and Fe^3+^ (714.9 eV) for RF‐1.25BFA; γ‐Fe_2_O_3_ (710.8 eV), Fe(III)─O/OH (712.0 eV) and Fe^3+^ (714.2 eV) for Cd(II)‐laden RF‐1.25BFA; Fe^0^ (706.2 eV), γ‐Fe_2_O_3_ (711.0 eV) and Fe^3+^ (714.4 eV) for RF‐1.25BFA‐540; γ‐Fe_2_O_3_ (710.6 eV), Fe(III)─O/OH (712.0 eV) and Fe^3+^ (714.0 eV) for Cd(II)‐laden RF‐1.25BFA‐540. As the adsorption proceeded, the concentration of γ‐Fe_2_O_3_ decreased, accompanied by a gradual rise in the concentration of γ‐FeOOH and a decrease in the concentration of Fe^3+^ (Equations (7), (8), (9), (10)). This was mainly due to the high‐alkalinity adsorption microenvironment, which favored the formation of γ‐FeOOH. Meanwhile, the reaction was more inclined to retrograde for RF‐1.25BFA (Equation 11). In contrast to RF‐1.25BFA, RF‐1.25BFA‐540, stored for 540 days, had only a very small amount of Fe^0^ oxidized to γ‐Fe_2_O_3_. However, the increased thickness might also have affected the XPS detection because the surface of iron clusters nanostructures was further covered by the newly generated γ‐Fe_2_O_3_. Furthermore, The Fe^0^ peaks of RF‐1.25BFA and RF‐1.25BFA‐540 were almost undetectable due to the formation of frosting nanostructures. The thickness of these frosting nanostructures likely increased in tandem with the increasing concentration of Cd(II).^[^ ^82^ ^]^ Figure S26 (Supporting Information) displayed the high‐resolution XPS spectra of Cd 3 d_5/2_. The Cd 3 d_5/2_ spectrum revealed that the Cd(II)‐laden RF‐CSMSs/RF‐1.25BFA/RF‐1.25BFA‐540 contained more than one type of Cd species. Among them, Cd(II)‐laden RF‐1.25BFA/RF‐1.25BFA‐540 exhibited different type of Cd species.^[^ ^83^ ^]^ The three peaks at 404.7, 405.6, and 406.0 eV were ascribed to Cd–O (71.66%), while the two peaks at 406.7 and 407.3 eV could be attributed to Cd(OH)^+^ (28.34%) for Cd(II)‐laden RF‐CSMSs. For Cd(II)‐laden RF‐1.25BFA, the peak at 404.5 eV may be ascribed to Cd–O (10.87%), and the three peaks at 405.5, 406.0 and 406.9 eV were consistent with Cd(OH)_2_ (89.13%). In the case of Cd(II)‐laden RF‐1.25BFA‐540, the peak at 404.0 eV was attributed to Cd–O (19.71%), while the three peaks at 405.3, 406.2 and 407.5 eV were consistent with Cd(OH)_2_ (80.29%). The presence of Cd(OH)_2_ peak indicated that Cd(II) had indeed undergone hydrolysis on the surface of RF‐1.25BFA and RF‐1.25BFA‐540. This phenomenon was explained by the rapid formation of Cd(OH)_2_ via the ultrafast two‐step enrichment–hydrolysis process on the surface in the favorable high‐alkalinity adsorption microenvironment constructed by iron clusters nanostructures (Equation 12), consistent with the record‐high adsorption capacity observed during the adsorption experiments. The C 1s spectrum was obtained to illustrate the details of functional groups as depicted in Figure S27 (Supporting Information). The C 1s peak of RF‐CSMSs, RF‐1.25BFA and RF‐1.25BFA‐540 could be fitted into four species at 284.8, 285.4–285.5, 288.8–288.9 and 291.2–291.9 eV, which can be assigned to C graphite/C─C/C─H, C─OH/C─O─C, C═O and π‐π* shake up, respectively.^[^ ^84^ ^]^ After Cd(II) adsorption, the C 1s peak of Cd(II)‐laden RF‐CSMSs, Cd(II)‐laden RF‐1.25BFA and Cd(II)‐laden RF‐1.25BFA‐540 was also divided into four species at 284.7–284.8, 285.5, 288.2–288.7 and 291.0–291.3 eV, which could be attributable to C graphite/C─C/C─H, C─OH/C─O─C, C═O and π‐π* shake up, respectively. The trend comparison of C 1s peak before and after Cd(II) adsorption was consistent with the analysis results of O 1s and Fe 2 p_3/2_ peaks. Additionally, the high‐resolution XPS spectra of C 1s, O 1 s and Fe 2 p_3/2_ in RF‐0.50BFA, RF‐0.75BFA, RF‐BFA and RF‐1.50BFA were also displayed for comparison (Figure S28, Supporting Information). Consequently, the adsorption of Cd(II) on RF‐1.25BFA and RF‐1.25BFA‐540 was an ultrafast two‐step enrichment–hydrolysis chemical adsorption process, which aligned well with the results derived from the D–R isotherm model analysis.

(1) $$C=C\pi -H_{3}O^{+}+Cd^{2+}\to \;C=C\pi -Cd^{2+}+H_{3}O^{+}$$

(2) $$C-\mathrm{OH}\;+Cd^{2+}+H_{2}O\;\to \;C-\mathrm{OC}d^{+}+H_{3}O^{+}$$

(3) $$Cd^{2+}+OH^{-}\;\to \;\mathrm{Cd}{\left(\mathrm{OH}\right)}^{+}$$

(4) $$Fe^{0}+2H_{2}O\;\to \;Fe^{2+}+H_{2}+2OH^{-}$$

(5) $$2Fe^{0}+O_{2}+2H_{2}O\;\to \;2Fe^{2+}+4OH^{-}$$

(6) $$4Fe^{2+}+O_{2\;}+2H_{2}O\;\to \;4Fe^{3+}+4OH^{-}$$

(7) $$4Fe^{0}+3O_{2}+2H_{2}O\;\to \;4\gamma -\mathrm{FeOOH}$$

(8) $$4Fe^{2+}+O_{2}+6H_{2}O\;\to \;4\gamma -\mathrm{FeOOH}+8H^{+}$$

(9) $$4Fe^{2+}+O_{2}+4H_{2}O\;\to \;2\gamma -Fe_{2}O_{3}+8H^{+}$$

(10) $$\gamma -Fe_{2}O_{3}+H_{2}O\;⇌\;2\gamma -\mathrm{FeOOH}$$

(11) $$\gamma -\mathrm{FeOOH}\left(s\right)+H_{2}O\;⇌\;Fe^{3+}\left(\mathrm{aq}\right)+3OH^{-}$$

(12) $$Fe^{0}@\gamma -Fe_{2}O_{3}+Cd^{2+}+2OH^{-}\to \;Fe^{0}@\gamma -Fe_{2}O_{3}-\mathrm{Cd}{\left(\mathrm{OH}\right)}_{2}$$

The changes of pH before and after Cd(II) adsorption by RF‐1.25BFA and RF‐1.25BFA‐540 at different pH conditions are presented in Figure 5b,d and Table S8 (Supporting Information). Interestingly, across the entire pH range studied, iron ions were not detected in the liquid phase after Cd(II) adsorption, or their concentration was below the detection line. This suggested that appropriate acidic conditions do not significantly damage the iron clusters nanostructures. The influence of lower pH on adsorption of Cd(II) can be reasonably explained by the competition between Cd(II) and H^+^ ions for active load sites on iron clusters nanostructures and hydroxyl groups on carbon spheres surface. During the entire process of Cd(II) adsorption in the lower pH region, the part of the H^+^ ions in the liquid phase were more readily consumed by high‐alkalinity adsorption microenvironment surrounding the carbon spheres. This not only weakened the interaction of Cd(II) and iron clusters nanostructures but also caused the high protonation of numerous hydroxyl groups on the carbon spheres surface, resulting in a slight pH rise upon reaching adsorption equilibrium. Besides the compelling advantages of high‐alkalinity adsorption microenvironment, the achievement of record‐high adsorption capacity is also inseparable from the repairmen of adsorption microenvironment by the hydroxyl anions from the liquid phase. In the higher pH region (pH > 5.7 for RF‐1.25BF and pH > 6.2 for RF‐1.25BFA‐540), the concentration of H^+^ ions was significantly lower than that of metal ions, resulting in most hydroxyl groups on the carbon spheres surface being utilized by Cd(II).^[^ ^85^ ^]^ As the hydroxyl groups in the high‐alkalinity adsorption microenvironment were consumed, the numerous hydroxyl anions in the liquid phase might enter the adsorption microenvironment under the induction of the iron clusters nanostructures, resulting in a trend of decreasing pH after reaching adsorption equilibrium. On the other hand, it also shows that RF‐1.25BFA and RF‐1.25BFA‐540 exhibited astonishing adsorption performance in weak acid environment, with RF‐1.25BFA being more advantageous than RF‐1.25BFA‐540. Based on our findings, it can be concluded that Cd(II) would undergo an ultrafast two‐step adsorption process of enrichment and hydrolysis (hydroxyl coordination process), with hydrolyzate (i.e., Cd(OH)_2_) being rapidly generated on the surface of iron clusters nanostructures of RF‐1.25BFA and RF‐1.25BFA‐540.^[^ ^86^ ^]^ Therefore, across the pH range studied, it was ascertained that the key to the new mechanism underlying its function likely lie in the iron clusters nanostructures. The possible mechanism can be explained as follows: these iron clusters nanostructures provide multiple active load sites and induce the creation of a high‐alkalinity adsorption microenvironment.^[^ ^87^ ^]^ Concurrently, the rich hydroxyl groups of carbon spheres surface contribute to the basic conditions necessary for a high‐alkalinity adsorption microenvironment.^[^ ^88^ ^]^ Detailed explanations were provided in the density functional theory (DFT) calculations section.

### 2.4 DFT Calculations for Cd(II) Adsorption Behavior on RF‐CSMSs and RF‐1.25BFA

**Figure 6:**
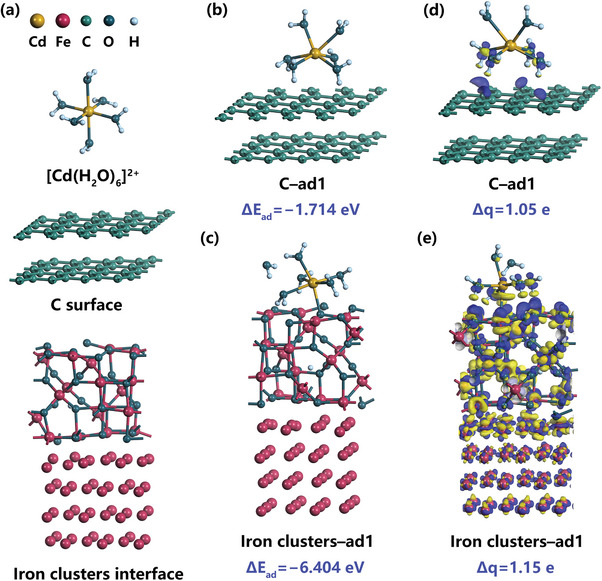
a) The optimized geometries of RF‐CSMSs (C surface) and RF‐1.25BFA (iron clusters interface). The optimized geometries of [Cd(H_2_O)_6_]^2+^ adsorption on b,d) C surface (C–ad1) and c,e) iron clusters interface (iron clusters–ad1) and the corresponding differential charge density. All views are front views. A positive value for Δq indicates electron accumulation and the isosurface levels are set to 0.02 and 0.04 e Å^−3^ for C surface and iron clusters interface, respectively. The blue region represented electron accumulation, while the yellow region denoted electron depletion.

**Figure 7:**
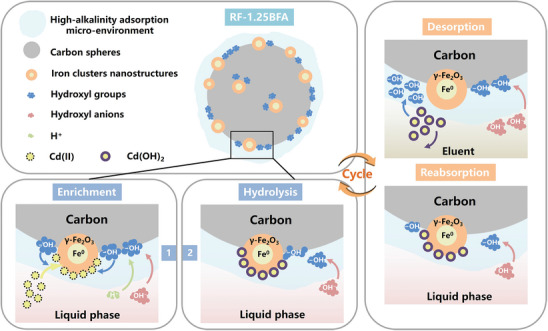
Schematic diagram of adsorption and adsorption–desorption cycle mechanisms for Cd(II) on RF‐1.25BFA.

Quantum chemical calculation, especially the DFT calculations, are increasingly effective for supplementing experimental results and gaining novel insight into chemistry‐related problems.^[^ ^89^ ^]^ To gain further insight into the adsorption behavior of Cd(II) on the iron clusters nanostructures of RF‐1.25BFA and verify the proposed Cd(II) adsorption mechanism, which consists of two successive ultrafast reaction steps: (1) enrichment and (2) hydrolysis, DFT calculations were carried out (Text S7). To accurately simulate the adsorption process, explicit water molecules in the first hydrated shell of cation were taken into account, and the coordination number (CN = 6) of Cd(II) was determined.^[^ ^90^ ^]^ Based on solution chemistry calculations, the predominant species of Cd(II), should be described as [Cd(H_2_O)_6_]^2+^ in the geometry optimization (**Figure** **6**a; Figure S29a, Supporting Information). The optimized geometries of RF‐CSMSs and iron clusters nanostructures of RF‐1.25BFA were referred to as the C surface and iron clusters interface, respectively (Figure 6a; Figure S29a, Supporting Information). Three initial adsorption models were adopted for both the C surface (Figure 6b; Figures S29b and S30a,b, Supporting Information) and iron clusters interface (Figure 6c; Figure S29c and S30c,d, Supporting Information). Assuming that [Cd(H_2_O)_6_]^2+^ can undergo a single adsorption process on RF‐CSMSs and iron clusters nanostructures of RF‐1.25BFA without direct interaction between [Cd(H_2_O)_6_]^2+^ and the substrate surface. Since the hydrated products of Cd(II) were more stable than the hydrolyzed products, and [Cd(H_2_O)_6_]^2+^ exhibited the highest stability, this implied that a single adsorption process may struggle to proceed to hydrolysis. In the C surface, the final state shown in C–ad1, with four H_2_O molecules interacting with the surface, achieved the strongest interaction (Figure 6b). Among all adsorption models, iron clusters–ad1 was highly representative. Figure 6c illustrated that during the interaction between [Cd(H_2_O)_6_]^2+^ and the iron clusters interface, Cd(II) consistently maintained a 6–coordination state. The chemical bonding between [Cd(H_2_O)_6_]^2+^ and the iron clusters interface facilitated the release of one H_2_O molecule, still retaining the 6–coordination state and forming a stable local bond with the iron clusters interface. This indicated that [Cd(H_2_O)_6_]^2+^ existed in a more active [Cd(H_2_O)_5_]^2+^ form via an adsorption–dissociation process in iron clusters–ad1 model, with reduced hydration and increased electron acceptance, creating more favorable conditions for further hydrolysis.^[^ ^91^ ^]^ The results suggested that iron clusters–ad1 might have been more conducive to a two‐step successive adsorption process involving enrichment and hydrolysis. Additionally, in iron clusters–ad3 model, which belongs to the single adsorption model, significant adsorption still occurred even without H_2_O dissociation, further corroborating the role of the iron clusters interface. On the other hand, the adsorption energy (ΔE_ad_, eV) in the iron clusters interface was significantly lower than in the C surface, energetically confirming the stronger interaction between the iron clusters interface and [Cd(H_2_O)_6_]^2+^ (Table S9, Supporting Information).^[^ ^92^ ^]^ Combined with the previous optimized geometries analysis, it was evident that the adsorption–dissociation mode in iron clusters–ad1 had the most negative adsorption energy, indicating a very strong interaction with the iron clusters interface, consistent with the hypothesized enrichment effect of the iron clusters nanostructures. The configuration also suggested that a new Cd–O bond might have formed on the iron clusters interface, laying the foundation for further hydrolysis on the iron clusters nanostructures. Moreover, due to the occurrence of adsorption–dissociation, this energy change was included in the adsorption energy calculation, resulting in a final adsorption energy significantly lower than that of the C surface. The comparison of optimized geometries adsorption energies indicated that the iron clusters nanostructures not only provided an excellent active load sites for Cd(II) adsorption but also facilitated hydrolysis, demonstrating the strong interaction between the adsorbate and the iron clusters interface. For visualizing the essence of charge transfer, the differential charge density between the substrate and [Cd(H_2_O)_6_]^2+^ were evaluated, as described in Figure 6d,e. Here, the blue region represented electron accumulation, and the yellow region denoted electron depletion. The iron clusters interface exhibited a significantly larger charge interaction area than the C surface, even at an isosurface level twice that of the C surface. In C–ad1, the electron‐deficient region of [Cd(H_2_O)_6_]^2+^ mainly involved the four H_2_O molecules in contact with the surface, with the surface region correspondingly gaining electrons (Figure 6d). In iron clusters–ad1, besides the four H_2_O molecules in contact with the surface losing electrons, Cd(II), which was clearly bonded with the surface, also showed a typical electron‐deficient state, with a large yellow region toward the surface, indicating a stronger tendency for electron transfer to the surface in the adsorption–dissociation mode of the iron clusters interface (Figure 6e). The phenomenon of charge accumulation and depletion was clearly observable for [Cd(H_2_O)_6_]^2+^ and iron clusters interface, which indicated the strong interaction between [Cd(H_2_O)_6_]^2+^ and iron clusters nanostructures. Cd(II)’s electron‐deficient state might have facilitated hydrolysis reaction with surface hydroxyl groups in the high‐alkalinity adsorption microenvironment. DFT calculations results further confirmed that Cd(II) adsorption on RF‐1.25BFA was an ultrafast successive two‐step enrichment–hydrolysis adsorption process. Combined with experimental data, the possible reaction mechanisms of RF‐CSMSs and RF‐1.25BFA were further clarified: the carbon spheres surface, due to its rich hydroxyl groups but without other inducing factors, underwent traditional complexation with Cd(II) for removal; RF‐xBFA, particularly RF‐1.25BFA, induced by the iron clusters nanostructures, underwent an ultrafast successive two‐step enrichment–hydrolysis adsorption process for Cd(II). In this process, enrichment occurred on the surface of iron clusters nanostructures, while hydrolysis was completed using the hydroxyl groups on the carbon spheres surface (Equation 13). As the hydrated product on the iron clusters nanostructures transitioned sequentially from [Cd(H_2_O)_6_]^2+^ to [Cd(H_2_O)_5_]^2+^ and [Cd(OH)_2_(H_2_O)_3_], the stability of the hydrated product decreased with the reduction of bound water. This could also be the reason why Cd(II) was more easily desorbed and underwent an extremely high number of adsorption cycles. The mechanisms of adsorption and adsorption–desorption cycle for Cd(II) on RF‐1.25BFA is shown in **Figure** **7**.

(13) $$\begin{array}{cc} & Fe^{0}@\gamma -Fe_{2}O_{3}-{\left[\mathrm{Cd}{\left(H_{2}O\right)}_{5}\right]}^{2+}+H_{2}O+2OH^{-}\\ & \;\to \;Fe^{0}@\gamma -Fe_{2}O_{3}-\left[\mathrm{Cd}{\left(\mathrm{OH}\right)}_{2}{\left(H_{2}O\right)}_{3}\right]+3H_{2}O \end{array}$$

### 2.5 Reusability and Adsorption Economic Analysis

**Figure 8:**
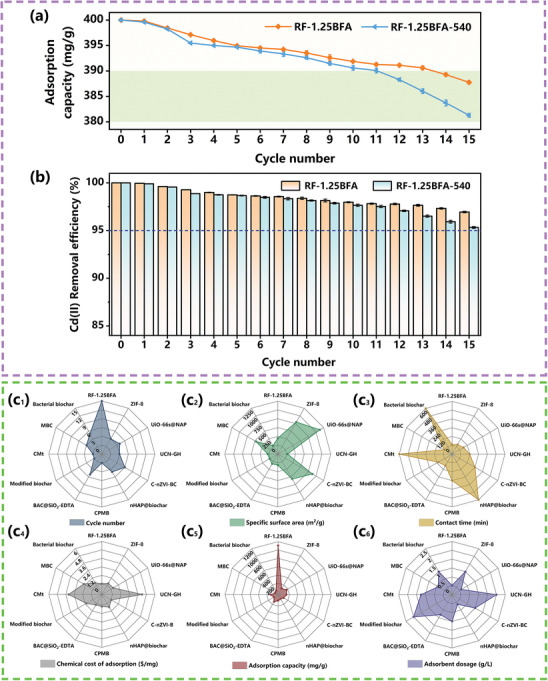
a) Adsorption capacity and b) removal efficiency of regenerated RF‐1.25BFA and RF‐1.25BFA‐540 for 15 adsorption–desorption cycles. Adsorption conditions: [Initial Cd(II)] = 10 mg L^−1^, adsorbent dosage = 0.025 g L^−1^, T = 25 °C, pH = 6.50 and equilibrium time = 720 min. Desorption conditions: desorbent: 0.1 M EDTA, adsorbent/EDTA solution = 70 mg/280 mL, T = 25 °C, contact time = 120 min. c_1_–c_6_) Comparison of the adsorption performances for heavy metals and the adsorption economic analysis between RF‐1.25BFA and other different adsorbents.

The reusability of adsorbents was a key factor in the treatment of heavy metal contaminated wastewater, especially mine drainage, and it had a significant impact on the operational costs of mine drainage treatment. For economic reasons, the regeneration of adsorbents was necessary. In the regeneration step, the primary goal was to restore the adsorptive capacity of the saturated adsorbent for further use in subsequent regeneration cycles.^[^ ^93^ ^]^ The more times the adsorbents could be regenerated while maintaining certain performance, the greater the practical application value. The recycling/regeneration parameters determine the capability and performance of adsorbent materials. Studies indicated that after 15 adsorption–desorption cycles, the adsorption capacities of regenerated RF‐1.25BFA and RF‐1.25BFA‐540 only slightly decreased, remaining as high as 387.76 and 381.28 mg g^−1^, respectively (**Figure** **8**a). Remarkably, the removal efficiencies of RF‐1.25BFA and RF‐1.25BFA‐540 for Cd(II) both stayed above 95% after 15 adsorption cycles, with the removal efficiency of RF‐1.25BFA even exceeding 96% (Figure 8b), indicating an exceptional level of stability and reusability for both RF‐1.25BFA and RF‐1.25BFA‐540. After completing one adsorption–desorption cycle, both RF‐1.25BFA and RF‐1.25BFA‐540 regenerated simultaneously to their initial forms, showing no discernible degradation in their original structures (Figure S31, Supporting Information). The thickness of the iron clusters nanostructures might have increased due to a small amount of residual γ‐FeOOH, leading to a significant discrepancy between the actual and detected results for Fe^0^ (Figure S32, Supporting Information).^[^ ^94^ ^]^ Moreover, hydroxyl groups on the carbon spheres surface that were originally involved in hydrolysis reactions in the high‐alkalinity adsorption microenvironment may have been partially “restored” due to the induction of the iron clusters nanostructures after desorption. This implies that hydroxyl groups on the carbon spheres surface may undergo multiple adsorption–desorption cycles in a peculiar “borrowing and returning” manner. Additionally, the regenerated RF‐1.25BFA and RF‐1.25BFA‐540 could induce hydroxyl anions from the liquid phase to enter the high‐alkalinity adsorption microenvironment after each cycle, continuing to repair the loss of hydroxyl groups. The economic feasibility of material application was a primary consideration for any industry choosing high‐performance materials.^[^ ^95^ ^]^ We conducted the adsorption economic analysis of comparing RF‐1.25BFA prepared in this study with other different adsorbent materials in terms of cycle number, specific surface area, contact time, chemical cost of adsorption, adsorption capacity and adsorbent dosage (Figure 8c_1_–c_6_; Table S10 and Text S1.4, Supporting Information). The comparative results confirmed that RF‐1.25BFA may have shown superior performance in treating various heavy metal contaminated wastewater in practical applications compared to traditional adsorbent materials, thereby potentially paving a new path for ultra‐efficient and cost‐effective treatment of mine drainage.

## 3 Conclusion

To summarize, we have designed and synthesized the novel CSMCs supported Fe^0^@γ‐Fe_2_O_3_ core‐shell clusters nanostructures, which can be flexibly applied for efficient and cost‐effective removal of trace amounts of Cd(II) from various types of water bodies. Kinetic and equilibrium adsorption studies demonstrated that RF‐1.25BFA and RF‐1.25BFA‐540 exhibited ultrafast adsorption kinetics and superior adsorption capabilities. The adsorption capacities for 10 mg L^−1^ Cd(II) were 400.00 mg g^−1^, achieving theoretical maximum adsorption capacities for Cd(II) of 1108.87 and 1065.06 mg g^−1^ using 0.025 g L^−1^ adsorbent, respectively, marking the record‐high level. The adsorption behavior adhered to the pseudo‐second‐order kinetic models, the Elovich model, Redlich–Peterson isotherm model, and D–R isotherm model, indicating that the adsorption process was predominantly a chemisorption‐dominated heterogeneous hybrid adsorption process. In practical applications, RF‐1.25BFA and RF‐1.25BFA‐540 maintained high adsorption capacities under extreme and more intricate environmental conditions, highlighting their viability for large‐scale practical implementation. Crucially, this study disclosed the ultrafast successive two‐step enrichment–hydrolysis adsorption mechanism for Cd(II) removal, pinpointing the pivotal role of iron clusters nanostructures in constructing a high‐alkalinity adsorption microenvironment on the materials surface. DFT calculations provided further validation of this mechanism. Of particular note, after 15 cycles, the regenerated RF‐1.25BFA and RF‐1.25BFA‐540 still maintained a remarkably high Cd(II) adsorption capacities, underscoring their outstanding stability and reusability. Particularly striking was the adsorption economic analysis of RF‐1.25BFA prepared in this study, which compared its performance with various other adsorbent materials, demonstrating RF‐1.25BFA's superior potential and broader prospects for practical engineering applications. These remarkable performances offered new insights and perspectives into the efficient removal mechanisms of trace amounts of heavy metals from wastewater using novel materials. They greatly improved the justification and feasibility of utilizing these materials in engineering treatments for mine drainage, setting the stage for the development of ultra‐efficient and cost‐effective treatments for industrial wastewater, acid mine drainage, and groundwater purification. The study also aims to emphasize the crucial importance of advancing the mechanisms by which novel materials remove trace amounts of heavy metals for future applications.

## Conflict of Interest

The authors declare no conflict of interest.

## Supporting information



Supporting Information

## Data Availability

The data that support the findings of this study are available from the corresponding author upon reasonable request.
