# Supplementary material for: Develop Reusable Carbon Sub‐Micrometer Composites with Record‐High Cd(II) Removal Capacity
Source: Adv Sci (Weinh). 2024 Nov 22;12(3):2408295. doi: 10.1002/advs.202408295 (PMC11744635; doi:10.1002/advs.202408295)
Supplement: Supplementary file 1 — Supporting Information [file ADVS-12-2408295-s001.pdf]

ADVANCED SCIENCE
Open Access

Supporting Information

for *Adv. Sci.*, DOI 10.1002/advs.202408295

Develop Reusable Carbon Sub-Micrometer Composites with Record-High Cd(II) Removal Capacity

Mengke Cui, Huiting Jiao, Shijie Yuan, Bin Dong and Zuxin Xu*

Supporting Information

Develop Reusable Carbon Sub-Micrometer Composites with Record-High Cd(II) Removal Capacity

Mengke Cui[a], Huiting Jiao[a], Shijie Yuan[a,b], Bin Dong[a,b,c,*], Zuxin Xu[a,b]

a State Key Laboratory of Pollution Control and Resource Reuse, College of Environmental Science and Engineering, Tongji University, Shanghai 200092, PR China.
b Shanghai Institute of Pollution Control and Ecological Security, Tongji University, Shanghai 200092, PR China.
c College of Environmental Science and Engineering, Guilin University of Technology, Guilin 541006, PR China.

Corresponding author email: dongbin@tongji.edu.cn (B. Dong).

* Corresponding author.
E-mail address: dongbin@tongji.edu.cn (B. Dong).

Content

Text S1. Experimental section

Text S2. Reagents and chemicals.

Text S3. Characterizations and analytical methods.

Text S4. Adsorption capacity and removal efficiency for Cd(II) on CSMCs.

Text S5. Adsorption kinetics for Cd(II) on CSMCs.

Text S6. Adsorption thermodynamics for Cd(II) on CSMCs.

Text S7. Theoretical calculation.

Table S1. Textural properties of CSMCs.

Table S2. Kinetics fitting parameters for Cd(II) adsorption on CSMCs at pH 6.50 and 25 °C.

Table S3. Parameters of adsorption isotherm models for Cd(II) adsorption on RF-1.25BFA and RF-1.25BFA-540 at pH 6.50 and 25 °C.

Table S4. The theoretical maximum adsorption capacities comparison between the RF-1.25BFA and various adsorbents for Cd(II) adsorption from the liquid phase.

Table S5. Kinetics fitting parameters for Cd(II) adsorption on RF-1.25BFA and RF-1.25BFA-540 at different temperatures (T = 5 °C, 15 °C, 25 °C, 35 °C) and pH 6.50.

Table S6. Parameters of adsorption thermodynamic for Cd(II) adsorption on RF-1.25BFA and RF-1.25BFA-540.

Table S7. Kinetics fitting parameters for Cd(II) adsorption on RF-1.25BFA and RF-1.25BFA-540 at different pH values (pH = 3.30, 5.00, 6.50, 7.60, 8.50) and 25 °C.

Table S8. The pH values change of the Cd(II) adsorption by RF-1.25BFA and RF-1.25BFA-540 under different pH conditions after equilibrium.

Table S9. Adsorption energy of Cd(II) by different optimized geometries in DFT calculations.

Table S10. Comparison of the performances and economic analysis of different adsorbents.

Figure S1. FE-SEM images with size scale of (a) RF-CSMSs and (b) RF-1.25BFA.

Figure S2. EDS elemental mappings of (a_1) C element, (a_2) O element of the RF-CSMSs; (b_1) C element, (b_2) O element and (b_3) Fe element of the RF-1.25BFA.

Figure S3. EDS elemental mappings of C, O and Fe elements of (a_1–a_4) RF-0.50BFA, (b_1–b_4) RF-0.75BFA, (c_1–c_4) RF-BFA, (d_1–d_4) RF-1.25BFA-540 and (e_1–e_4) RF-1.50BFA.

Figure S4. XRD pattern of RF-CSMSs.

Figure S5. TEM images (a, b) RF-1.25BFA and (d, e) RF-1.25BFA-540. Selected-area electron diffraction (SAED) patterns (c) RF-1.25BFA and (f) RF-1.25BFA-540.

Figure S6. (a) N_2 absorption–desorption isotherms and (b) pore size distribution curves of the as-prepared CSMCs.

Figure S7. The thermogravimetric characterization of RF-CSMSs, RF-1.25BFA and RF-1.25BFA-540 under air (a, b) and argon (c, d).

Figure S8. Effect of adsorbent dosage on the adsorption capacity of RF-1.25BFA. Conditions: [Initial Cd(II)] =100 mg/L, adsorbent dosage = 0.0125 g/L, 0.02 g/L, 0.025 g/L, 0.03 g/L, 0.04 g/L, 0.05 g/L, 0.06 g/L, 0.1 g/L, 0.2 g/L and 0.6 g/L, T = 25 °C, pH = 6.50 and equilibrium time = 720 min.

Figure S9. Adsorption kinetics for Cd(II) adsorption on (a) RF-CSMSs, (b) RF-0.50BFA, (c) RF-0.75BFA, (d) RF-BFA and (e) RF-1.50BFA: the pseudo-first-order, pseudo-second-order and Elovich equation model fitting curves. Conditions: [Initial Cd(II)] =10 mg/L, adsorbent dosage = 0.025 g/L, T = 25 °C, pH = 6.50 and equilibrium time = 720 min.

Figure S10. Intraparticle diffusion model fitting curve for Cd(II) adsorption on (a) RF-CSMSs, (b) RF-0.50BFA, (c) RF-0.75BFA, (d) RF-BFA and (e) RF-1.50BFA. Conditions: [Initial Cd(II)] =10 mg/L, adsorbent dosage = 0.025 g/L, T = 25 °C, pH = 6.50 and equilibrium time = 720 min.

Figure S11. Dubinin–Radushkevich (D-R) isotherm plots obtained for Cd(II) adsorption on (a) RF-1.25BFA and (b) RF-1.25BFA-540. Conditions: [Initial Cd(II)] = 5–300 mg/L, adsorbent dosage = 0.025 g/L, T = 25 °C, pH = 6.50 and equilibrium time = 720 min.

Figure S12. Effect of temperature towards adsorption performance of (a) RF-1.25BFA and (b) RF-1.25BFA-540 for Cd(II). Adsorption capacity of (c) RF-1.25BFA and (d) RF-1.25BFA-540 at different temperature. Conditions: [Initial Cd(II)] =10 mg/L, adsorbent dosage = 0.025 g/L, T = 5 °C, 15 °C, 25 °C, 35 °C, pH = 6.50 and equilibrium time = 720 min.

Figure S13. Effect of temperature towards adsorption kinetics for Cd(II) adsorption on (a–d) RF-1.25BFA and (e–h) RF-1.25BFA-540: the pseudo-first-order, pseudo-second-order and Elovich equation model fitting curves. Conditions: [Initial Cd(II)] =10 mg/L, adsorbent dosage = 0.025 g/L, T = 5 °C, 15 °C, 25 °C, 35 °C, pH = 6.50 and equilibrium time = 720 min.

Figure S14. Effect of pH towards adsorption kinetics for Cd(II) adsorption on (a–e) RF-1.25BFA and (f–j) RF-1.25BFA-540: the pseudo-first-order, pseudo-second-order and Elovich equation model fitting curves. Conditions: [Initial Cd(II)] =10 mg/L, adsorbent dosage = 0.025 g/L, T = 25 °C, pH = 3.30, 5.00, 6.50, 7.60, 8.50 and equilibrium time = 720 min.

Figure S15. Zeta potential curves of RF-1.25BFA and RF-1.25BFA-540.

Figure S16. Effect of different concentrations (5 and 10 mg/L) of competitive (a, b) cations and (c, d) anions for Cd(II) adsorption on RF-1.25BFA and RF-1.25BFA-540. Conditions: [Initial Cd(II)] =10 mg/L, adsorbent dosage = 0.025 g/L, T = 25 °C, pH = 6.50 and equilibrium time = 720 min.

Figure S17. Removal efficiency of different concentrations (a) 5 mg/L and (b) 10 mg/L of competitive cations in competitive systems. Conditions: [Initial Cd(II)] =10 mg/L, adsorbent dosage = 0.025 g/L, T = 25 °C, pH = 6.50 and equilibrium time = 720 min.

Figure S18. Effect of solution ionic strength and electrolyte type (a) NaCl and (b) $NaNO_3$ for Cd(II) adsorption on RF-1.25BFA and RF-1.25BFA-540. Conditions: [Initial Cd(II)] =10 mg/L, adsorbent dosage = 0.025 g/L, T = 25 °C, pH = 6.50 and equilibrium time = 720 min.

Figure S19. XRD patterns of RF-1.25BFA and RF-1.25BFA-540 after Cd(II) adsorption.

Figure S20. FE-SEM image and EDS elemental mappings of RF-CSMSs after Cd(II) adsorption.

Figure S21. XRD pattern of RF-CSMSs after Cd(II) adsorption.

Figure S22. FE-SEM images of (a) RF-1.25BFA, (b) RF-1.25BFA-540 and (c) RF-CSMSs after the same adsorption process in the liquid phase without Cd(II).

Figure S23. The high-resolution XPS spectra of O 1 s in (a) RF-CSMSs and (b) Cd(II)-laden RF-CSMSs.

Figure S24. Wetting behavior of (a) RF-CSMSs, (b) RF-0.50BFA, (c) RF-0.75BFA, (d) RF-BFA, (e) RF-1.25BFA, (f) RF-1.25BFA-540 and (g) RF-1.50BFA toward water in air.

Figure S25. Wetting behavior of (a) RF-1.25BFA and (b) RF-1.25BFA-540 after Cd(II) adsorption toward water in air.

Figure S26. The high-resolution XPS spectra of Cd 3 $d_{5/2}$ in (a) Cd(II)-laden RF-CSMSs, (b) Cd(II)-laden RF-1.25BFA and (c) Cd(II)-laden RF-1.25BFA-540.

Figure S27. The high-resolution XPS spectra of C 1 s in (a) RF-CSMSs, (b) Cd(II)-laden RF-CSMSs, (c) RF-1.25BFA, (d) Cd(II)-laden RF-1.25BFA, (e) RF-1.25BFA-540 and (f) Cd(II)-laden RF-1.25BFA-540.

Figure S28. The high-resolution XPS spectra of (a–d) C 1 s, (e–h) O 1 s and (i–l) Fe 2 $p_{3/2}$ in RF-0.50BFA, RF-0.75BFA, RF-BFA and RF-1.50BFA.

Figure S29. (a) The optimized geometries of RF-CSMSs (C surface) and RF-1.25BFA (iron clusters interface). The optimized geometries of $[Cd(H_2O)_6]^{2+}$ adsorption on (b) C surface (C–ad1) and (c) iron clusters interface (iron clusters–ad1). All views are top views.

Figure S30. The optimized geometries of $[Cd(H_2O)_6]^{2+}$ adsorption on (a) C–ad2, (b) C–ad3, (c) iron clusters–ad2 and (d) iron clusters–ad3. The views include both front and top views.

Figure S31. FE-SEM images of (a) RF-1.25BFA and (b) RF-1.25BFA-540 after one adsorption-desorption cycle for Cd(II).

Figure S32. The high-resolution XPS spectra of (a–b) C 1 s, (c–d) O 1 s and (e–f) Fe 2 $p_{3/2}$ in RF-1.25BFA and RF-1.25BFA-540 after one adsorption–desorption cycle for Cd(II).

Supplementary text

Text S1. Experimental section

1.1. Synthesis of RF-CSMSs

In a typical experimental procedure, resorcinol and formaldehyde solutions were used as precursors to synthesize resorcinol/formaldehyde (RF) resin spheres.[1] Generally, 0.6 mL of ammonia was mixed with 120 mL of deionized water, and 48 mL

of ethanol, followed by vigorous stirring for 30 min. Subsequently, 1.2 g of resorcinol was introduced into the solution and stirred continuously for another 30 min. Next, 1.68 mL of formaldehyde solution was added gradually to the reaction mixture, followed by stirring for 24 h in a water bath at 30 ± 0.2 °C. The resulting solution was moved to a 100 mL sealed stainless steel autoclave with a Teflon liner and maintained in an oven at 100 °C for 24 h. Afterward, the autoclave was cooled to room temperature. The precipitate was gathered through centrifugation and subjected to thorough washing with deionized water and ethanol several times to remove any impurities. The RF resins spheres were then obtained through oven-drying at 60 °C for more than 12 h. For carbonization, the RF resins spheres were calcined in N_2 flow at 800 °C for 1 h, using a heating rate of 5 °C/min, yielding RF carbon sub-micrometer spheres (RF-CSMSs). Upon natural cooling to room temperature (25 °C), the resulting samples were labeled as RF-CSMSs.

1.2. Synthesis of RF-xBFA

The RF resins spheres (0.6 g), prepared beforehand, were ultrasonically dispersed in 180 mL of deionized water for 30 min. Subsequently, basic ferric acetate (BFA) was added into the above suspension, followed by a reaction at 30 ± 0.2 °C for 12 h in water bath with continuous stirring. The loading of iron clusters was controlled by adjusting the dosage of BFA (0.30, 0.45, 0.60, 0.75 and 0.90 g). After the reaction, the dark brown products were separated by sedimentation, thoroughly washed with deionized water, and then dried at 60 °C for 12 h. Carbonization was then performed in a tubular furnace under N_2 atmosphere, with the temperature held at 800 °C for 1 hour and a heating rate of 5 °C/min. All products were stored under normal laboratory conditions without any special requirements. The corresponding products were named as RF-xBFA, where x represents the initial mass ratio of BFA to RF resins spheres. The final products were

denoted as RF-0.50BFA, RF-0.75BFA, RF-BFA, RF-1.25BFA, RF-1.50BFA respectively. For comparison, RF-1.25BFA-540 was obtained by storing RF-1.25BFA in a normal environment for 540 days. The storage environment was an aerobic room temperature setting without additional humidity control and did not require any special protective gases. Detailed information on chemical reagents and related characterization can be found in the Text S2 and S3.

1.3. Batch adsorption experiments and analysis

Batch adsorption experiments were carried out to examine the effects of heavy metal adsorption on the different adsorbents, employing PET plastic bottles of either 200 mL or 300 mL in volume. A stock solution of Cd(II) (1000 mg/L) was made by dissolving $Cd(NO_3)_2 \cdot 4H_2O$ in deionized water and then diluting it to the required concentrations before use. All Cd(II) solutions contained 0.01 M $NaNO_3$ as the background electrolyte unless otherwise specified. For the adsorption experiments, 4 mg of the composites were introduced into 160 mL Cd(II) solution (10 mg/L) in the bottles. The suspension was initially adjusted to a pH of 6.50 ± 0.02 by adding either 0.1 M HNO_3 or NaOH solution with negligible volume change. All bottles were sealed and agitated in a temperature-controlled shaker at 200 rpm, maintaining a stable temperature of 25 ± 1 °C for a duration of 12 h. In the kinetic experiment, 10 mg/L Cd(II) solutions were shaken for different durations (1 min, 5 min, 10 min, 30 min, 1 h, 1.5 h, 2h, 4 h, 8 h and 12 h). To investigate the adsorption isotherms, experiments were conducted at 25 °C with an equilibrium duration of 12 h. The initial concentration of Cd(II) in the solution varied from 2 to 300 mg/L.

The study investigated the influences of pH, temperature, ionic strength, adsorbent dosage and competitive cations and anions on the adsorption of Cd(II) by the composites. To determine the effect of each parameter, the other conditions were

maintained consistently. For pH dependence, the initial pH of the reaction solution was varied from 3.30 to 8.50. Except for the pH dependence experiment, all other tests were conducted with an initial pH of 6.50. To assess the impact of pH on Cd(II) adsorption by the adsorbent, the initial pH was modified to 3.30, 5.00, 6.50, 7.60, and 8.50. In addition, the pH value of the solution was also measured after equilibrium at different pH conditions. The influence of temperature on Cd(II) adsorption and adsorption thermodynamics were studied at different temperatures (5, 15, 25 and 35 °C). Additionally, different concentrations $NaNO_3$ and NaCl (0.001, 0.01, 0.02, 0.05 and 0.1 M) were separately added to the mixture of Cd(II) and adsorbent suspension to examine the impact of ionic strength on the adsorption capacity. Moreover, the effect of adsorbent dosage was investigated by varying the dosage (0.0125, 0.020, 0.025, 0.03, 0.04, 0.05, 0.06, 0.1, 0.2 and 0.6 g/L) at a fixed pH (6.50 ± 0.02) and a temperature of 25 °C. The influences of competitive cations (Na^+, K^+, Ca^{2+}, Zn^{2+}, Mg^{2+}, Cu^{2+}, Al^{3+}, Pb^{2+}, Fe^{3+}, Mn^{2+} and Co^{2+}) and anions (NO_3^-, Cl^-, SO_4^{2-}, HSO_4^-, PO_4^{3-}, CO_3^{2-} and HCO_3^-) at concentrations of 5 and 10 mg/L on Cd(II) adsorption were examined under identical experimental conditions. Once the adsorption experiments were completed, the mixtures were collected and promptly filtered through an aqueous filter membrane with a pore size of approximately 0.22 μm. The filtrates were promptly analyzed by ICP-OES. The adsorbed Cd(II) amounts were calculated by subtracting the equilibrium solution concentration from the initial ion contents. All batch experiments were performed three times in parallel, and the average data were used in the analysis. It should be emphasized that the initial concentration of all the solutions was measured before adsorption. Control experiments confirmed that the loss of Cd(II) due to adsorption on the inner walls of the PET plastic bottles could be ignored under conditions identical to the adsorption process but without adsorbents. Detailed

information of the adsorption capacity at equilibrium q_e (mg/g) and the removal efficiency R (%) on adsorbent for Cd(II) is presented in Text S4. The adsorption kinetics models, adsorption isotherm models and adsorption thermodynamic are described in Text S5 and S6, respectively. The detailed calculations for DFT can be found in Text S7.

1.4. Desorption and reusability

The feasibility of desorption and regeneration of the adsorbent was investigated using 0.1 M EDTA as the eluent. RF-1.25BFA and RF-1.25BFA-540 adsorbents were used for this study, and each adsorption–desorption experiment was carried out simultaneously in two parallel experiments. In the adsorption–desorption process, 7 mg of adsorbent was agitated with 280 mL of Cd(II) solution (initial concentration 10 mg/L) under optimum conditions. After adsorption, the Cd(II)-laden adsorbent was washed, dried, and added to a 0.1 M EDTA solution (adsorbent : EDTA solution = 70 mg : 280 mL) for desorption at 200 rpm and 25 °C for 2 h. After extraction and filtration, the adsorbent was thoroughly washed with ethanol and deionized water to remove residual EDTA and Cd(II), then dried for successive adsorption-desorption cycles. Three sample solutions were randomly selected in each group for each cycle, and the corresponding measurement data are presented as means ± standard deviations. This process was repeated for 15 adsorption-desorption cycles. The recycle adsorption experimental procedures and detection methods followed those of the initial adsorption experiment. The regeneration performances of RF-1.25BFA and RF-1.25BFA-540 was evaluated based on their adsorption capacity and Cd(II) removal efficiency.

Based on the adsorption and desorption procedures, the chemical cost for Cd(II) adsorption is calculated as follows:[2]

$$Chemical\ cost\ of\ adsorption\ (\frac{\$}{mg}) = \frac{[A + (\sum_{i=1}^{n} B_i)]}{(n+1)} \quad \mathbf{(1)}$$

Where, A represents the cost for 1st adsorption cycle ($/mg), B represents the cost per regeneration cycle ($/mg/cycle), and n is the number of regeneration cycles. The denominator in the formula is $n + 1$ because regeneration starts after the 1st cycle. A depends on the adsorbent cost and adsorption capacity, and can be expressed as:

$$A = a_1/a_2 \tag{2}$$

Where, a_1 denotes the cost of the adsorbent ($/mg adsorbent) and a_2 denotes the adsorption capacity of the adsorbent (mg/g). The cost per regeneration cycle can be further broken down as:

$$B = (C + D + E)/a_2 \tag{3}$$

Where, C is the cost per desorption cycle ($/mg/cycle), D is the cost per acid/alkali wash cycle ($/mg/cycle), and E is the cost per ethanol/deionized water wash cycle ($/mg/cycle).

Text S2. Reagents and chemicals.

Cadmium nitrate tetrahydrate ($Cd(NO_3)_2 \cdot 4H_2O$), ethanol (≥99.8%), ammonia aqueous solution ($NH_3 \cdot H_2O$; 25–28 wt%), formaldehyde solution (HCHO; 37–40 wt%), resorcinol and ethylenediaminetetraacetic acid (EDTA) were provided by Shanghai Titan Scientific Co., Ltd. BFA was purchased from Shanghai Macklin Biochemical Technology Co., Ltd. Other inorganic metal salt reagents were purchased from Sinopharm Chemical Reagent Co., Ltd. All chemicals and regents were all of analytical reagent grade and used without further purification. Deionized water was used throughout the experiments.

Text S3. Characterizations and analytical methods.

The morphologies and sizes of the carbon sub-micrometer composites (CSMCs) including RF-CSMSs and RF-xBFA were characterized by field emission scanning electron microscopy (FE-SEM, GeminiSEM 300, ZEISS, Germany) equipped with an

energy-dispersive X-ray analyzer to fulfill element microanalysis and transmission electron microscopy (TEM, JEM 2100F, JEOL, Japan). The X-ray diffraction (XRD) patterns were collected to identify any crystallographic structures for the CSMCs by using a powder X-ray diffractometer with Cu Kα radiation (XRD, SmartLab SE, Rigaku, Japan). N_2 adsorption–desorption isotherms were obtained at 77 K on a ASAP2460 instrument. A vibrating-sample magnetometer (VSM, Lake Shore-7404) was used to determine the magnetic performance of CSMCs. Raman spectra were measured on a Raman spectrometer (Edinburgh RM5/XploRA) with a 514 nm excitation source. TGA measurements were carried out on a TA Q600 analyzer from 50 °C to 1000 °C under air or argon with a heating rate of 5 °C/min. X-ray photoelectron spectroscopy (XPS) measurements were performed using a spectrometer (Thermo Scientific K-Alpha, USA) equipped with an Al Kα X-ray as the excitation source. The zeta potentials at different pH values (3.30, 5.00, 6.50, 7.60 and 8.50) were measured by using a Zeta sizer (Nano ZS90, Malvern, UK). The contact angles (CAs) of CSMCs were based on the means of at least five independent measurements with the apparatus (JC 2000A, Powereach Co., Shanghai, China) at ambient temperature. The concentrations of Cd(II) and other cations were measured by inductively coupled plasma optical emission spectrometer (ICP-OES, 220 Max, Avio, Japan), with a detection limit of 0.0001 mg/L.

Text S4. Adsorption capacity and removal efficiency for Cd(II) on CSMCs.

The equilibrium adsorption capacity q_e (mg/g) and the removal efficiency R (%) for Cd(II) could be calculated according to the following equations: [3]

$$q_e = \frac{V(C_0 - C_e)}{m} \tag{4}$$

$$R = \frac{C_0 - C_e}{C_0} \times 100\% \tag{5}$$

where q_e (mg/g) is the amount of heavy metal adsorbed at equilibrium; C_0 and C_e (mg/L) are initial and equilibrium concentrations of Cd(II) in the solution, respectively; V (L) is the volume of solution, and m (g) is the added amount of adsorbent.

Text S5. Adsorption kinetics for Cd(II) on CSMCs.

The fitting of kinetic experimental data is conducted using pseudo-first-order model (eq 6),[4] pseudo-second-order model (eq 7),[5] Elovich model (eq 8)[6] and intraparticle diffusion model (eq 9).[7]

$$q_t = q_e(1 - e^{-K_1 t}) \quad (6)$$

$$q_t = \frac{K_2 q_e^2 t}{1 + K_2 q_e t} \quad (7)$$

$$q_t = A + B \ln t \quad (8)$$

$$q_t = k_i t^{1/2} + C \quad (9)$$

where q_e and q_t are the amount of Cd(II) adsorbed at the equilibrium and at time t (min), respectively (mg/g); and K_1 (min^{-1}) and K_2 (g·mg^{-1}·min^{-1}) are the rate constants of the pseudo-first-order and pseudo-second-order adsorption, respectively; A and B are the Elovich constants; C is the constant proportional to the extent of boundary layer thickness (mg/g) and k_i is the intraparticle diffusion rate constant (mg·g^{-1}·min$^{-0.5}$) of adsorption step; C and k_i can be obtained from the plot of q_t versus $t^{1/2}$.

Text S6. Adsorption thermodynamics for Cd(II) on CSMCs.

Four isotherm equations including Langmuir model (eq 10),[8] Freundlich model (eq 11),[9] Redlich–Peterson model (eq 12)[6b] and Dubinin–Radushkevich (D-R) model (eq 13)[10] model are used to fit the adsorption experimental data.

$$q_e = \frac{q_m K_L C_e}{1 + K_L C_e} \quad (10)$$

$$q_e = K_F C_e^{1/n} \quad (11)$$

$$q_e = \frac{K_R C_e}{1 + \alpha C_e^{\beta}} \quad (12)$$

$$\ln q'_e = \ln q'_m - K_D \varepsilon^2 \quad (13)$$

Where q_e is the equilibrium adsorption capacity for Cd(II) (mg/g); K_L and q_m are Langmuir constant and the maximum adsorption capacity of the adsorbents (mg/g), respectively; K_F is Freundlich constant, and n is the heterogeneity factor related to the adsorption intensity of the adsorbent; K_R and α are the Redlich–Peterson constants, and β is the exponent ($0 < \beta < 1$); q'_e is the amount of metal ions sorbed per unit weight of adsorbent (mol/L); q'_m is the maximum amount of metal ions sorbed per unit weight of adsorbent (mol/L); K_D is the activity coefficient related to the mean free energy of adsorption (mol^2/J^2); and ε is the Polanyi potential ($\varepsilon = RT\ln(1 + 1/C_e)$, J/mol; C_e, g/L).

The mean free energy of adsorption (E; kJ/mol) is expressed by eq 14:[11]

$$E = \frac{1}{\sqrt{2K_D}} \quad (14)$$

The thermodynamic parameters (namely, the Gibbs free energy (ΔG^{θ}, kJ/mol), enthalpy (ΔH^{θ}, kJ/mol), and entropy (ΔS^{θ}, $kJ \cdot mol^{-1} \cdot K^{-1}$) are calculated using eqs 15–16:

$$\Delta G^{\theta} = -RT\ln K_0 = -RT\ln(\frac{mq_e}{C_e V}) \quad (15)$$

$$\Delta G^{\theta} = \Delta H^{\theta} - T\Delta S^{\theta} \quad (16)$$

where R is the universal gas constant ($8.314\ J \cdot mol^{-1} \cdot K^{-1}$) and T is the temperature (K); K_0 is the thermodynamic equilibrium constant in the adsorption process, which was determined using the method of Khan and Singh[12] by plotting $\ln q_e/C_e$ versus q_e and extrapolating to zero q_e. The values of ΔH^{θ} and ΔS^{θ} can be evaluated from the intercept and the slope, respectively, of the linear plots of ΔG^{θ} versus T.

Text S7. Theoretical calculation.

In this work, all the density functional theory (DFT) calculations were performed using the first-principles calculation implementation of CASTEP.[13] The exchange–correlation potential was described using the generalized gradient approximation (GGA)[14] and the Perdew–Burke–Ernzerhof (PBE) formula,[15] combined with the DFT-D correction. Spin-polarized calculations were employed. The Broyden–Fletcher–Goldfarb–Shanno (BFGS) method was used to search for the ground state of the supercells, and the convergence tolerance was set to an energy change below 10−5 eV per atom, force less than 0.02 eV $Å^{-1}$, stress less than 0.05 GPa and displacement change less than 0.001 Å. The cut off energy of the atomic wave functions was set to 450 eV. During the optimizations, the bottom layers in catalysts were fixed in all directions. The optimized crystal structures of RF-CSMSs (C surface) and RF-1.25BFA (iron clusters interface) were all cleaved as a slab model under periodic boundary condition to represent the surface structure. The iron clusters interface model was constructed by splicing Fe (110) and Fe_2O_3-H (001). We would like to thank Shiyanjia Lab (www.shiyanjia.com) for support during the DFT test.

The adsorption energy (ΔE_{ad}, eV) was calculated according to eq 17:

$$\Delta E_{ad} = \Delta E_{A-B} - \Delta E_A - \Delta E_B \quad \mathbf{(17)}$$

Where ΔE_{A-B}, ΔE_A and ΔE_B are the total energies of adsorption system, substrate system and adsorbed substance, respectively.

Supplementary table

Table S1. Textural properties of CSMCs.

Adsorbents	S_{BET}[a] ($m^2 \cdot g^{-1}$)	S_{micro}[b] ($m^2 \cdot g^{-1}$)	S_{meso}[b] ($m^2 \cdot g^{-1}$)	V_{total}[c] ($cm^3 \cdot g^{-1}$)	V_{micro}[b] ($cm^3 \cdot g^{-1}$)	V_{meso}[b] ($cm^3 \cdot g^{-1}$)	w_{BJH}[d] (nm)	w_{meso}[d] (nm)
RF CSMSs	670.72	629.44	41.28	0.26	0.24	0.02	1.57	3.34

RF-0.50BFA	281.33	74.47	206.86	0.28	0.03	0.25	4.05	5.14
RF-0.75BFA	225.03	49.34	175.69	0.28	0.02	0.26	4.82	5.87
RF-BFA	206.59	39.76	166.83	0.22	0.02	0.20	4.16	4.94
RF-1.25BFA	199.68	55.35	144.33	0.22	0.02	0.20	4.46	5.52
RF-1.25BFA-540	179.68	59.96	119.72	0.24	0.03	0.21	4.98	6.68
RF-1.50BFA	181.44	52.08	129.36	0.19	0.02	0.17	4.32	5.29

[a]S_{BET} is the total specific surface area obtained from multipoint BET analysis. [b]Microporous (S_{micro}), and mesoporous (S_{meso}) surface area; microporous (V_{micro}) and mesoporous (V_{meso}) volume are given from t-plot analysis. [c]Total (V_{total}) pore volume is single point pore volume calculated from the adsorption isotherm at P/P_0 = 0.95. [d]Mean pore sizes (w_{BJH}) and mean mesoporous sizes (w_{meso}) are given from BJH analysis.

Table S2. Kinetics fitting parameters for Cd(II) adsorption on CSMCs at pH 6.50 and 25 °C.

Adsorbents	Pseudo-first-order model			
	$q_{e,cal}$a	$q_{e,exp}$b	K_1 (min^{-1})	R^2
RF-CSMSs	267.76	285.20	1.60	0.9540
RF-0.50BFA	362.82	369.88	2.56	0.9924
RF-0.75BFA	369.90	379.60	2.44	0.9895
RF-BFA	376.89	383.24	2.70	0.9949
RF-1.25BFA	399.73	400.00	5.02	0.9999
RF-1.25BFA-540	395.88	400.00	2.89	0.9979
RF-1.50BFA	379.57	384.72	2.93	0.9979
**Adsorbents**	**Pseudo-second-order model**			
	$q_{e,cal}$a	$q_{e,exp}$b	K_2 (g·mg^{-1}·min^{-1})	R^2
RF-CSMSs	275.18	285.20	0.008	0.9706
RF-0.50BFA	366.25	369.88	0.025	0.9947
RF-0.75BFA	371.59	379.60	0.026	0.9930
RF-BFA	378.67	383.24	0.031	0.9968
RF-1.25BFA	400.12	400.00	0.210	0.9999

RF-1.25BFA-540	396.28	400.00	0.051	0.9986
RF-1.50BFA	380.20	384.72	0.052	0.9986

Adsorbents	Elovich model		
	A	B	R^2
RF-CSMSs	216.45	11.80	0.9963
RF-0.50BFA	336.41	6.06	0.9985
RF-0.75BFA	338.32	7.28	0.9987
RF-BFA	353.60	5.33	0.9991
RF-1.25BFA	397.91	0.40	0.9999
RF-1.25BFA-540	377.91	4.05	0.9994
RF-1.50BFA	362.33	3.91	0.9997

Adsorbents	Intraparticle diffusion model					
	$k_{i,1}$ (mg·g^{-1}·min$^{-0.5}$)	R^2	$k_{i,2}$ (mg·g^{-1}·min$^{-0.5}$)	R^2	$k_{i,3}$ (mg·g^{-1}·min$^{-0.5}$)	R^2
RF-CSMSs	214.24	1	6.50	0.8996	0.54	0.5797
RF-0.50BFA	334.80	1	6.21	0.9903	0.27	0.4860
RF-0.75BFA	337.64	1	6.02	0.9794	0.54	0.5010
RF-BFA	351.64	1	4.83	0.9822	0.29	0.4465
RF-1.25BFA	397.08	1	1.14	0.9994	0.01	0.5149
RF-1.25BFA-540	373.88	1	7.15	0.9998	0.32	0.5070
RF-1.50BFA	359.40	1	5.83	0.8851	0.49	0.6268

a The calculated adsorption capacity at equilibrium, namely q_e in eq 3 and eq 4.

b The measured adsorption capacity at equilibrium.

Table S3. Parameters of adsorption isotherm models for Cd(II) adsorption on RF-1.25BFA and RF-1.25BFA-540 at pH 6.50 and 25 °C.

Adsorbents	Langmuir model		
	q_m (mg/g)	K_L (L/mg)	R^2
RF-1.25BFA	1108.87 ± 22.64	0.32 ± 0.06	0.9468
RF-1.25BFA-540	1065.06 ± 23.46	0.28 ± 0.05	0.9401
Adsorbents	Freundlich model		

	$1/n$	K_F ($mg^{1-n}\cdot L^n\cdot g^{-1}$)	R^2
RF-1.25BFA	0.12 ± 0.01	599.65 ± 12.24	0.9130
RF-1.25BFA-540	0.13 ± 0.01	550.61 ± 11.55	0.9072

Adsorbents	Redlich–Peterson model			
	α	β	K_R	R^2
RF-1.25BFA	0.23 ± 0.11	1.03 ± 0.04	294.20 ± 79.78	0.9499
RF-1.25BFA-540	0.19 ± 0.09	1.03 ± 0.04	238.09 ± 67.67	0.9435

Adsorbents	Dubinin–Radushkevich model			
	q'_m (mol/L)	K_D (mol^2/J^2)	E (kJ/mol)	R^2
RF-1.25BFA	2.69×10^{-4}	2.40×10^{-9}	14.43	0.9832
RF-1.25BFA-540	2.56×10^{-4}	2.56×10^{-9}	13.98	0.9907

Table S4. The theoretical maximum adsorption capacities comparison between the RF-1.25BFA and various adsorbents for Cd(II) adsorption from the liquid phase.

Adsorbents	pH	T (°C)	Dosage (g/L)	Adsorption capacity (mg/g)	Refs.
RF-1.25BFA	6.50	25	0.025	1108.87	This work
**Biochar and biomass adsorbents**					
Papaya wood	5.00	25	5.0	1.89	[16]
Prickly pear cactus cladodes	5.80	25	2.0	30.42	[17]
PAMAM dendrimer/apple residue biosorbents	6.00	25	1.0	161.87	[18]
Algal biomass	4.00	20	0.75	111.10	[19]
Red mud modified bean-worm skin biochars	7.50	20	1.67	73.52	[20]
NaOH-modified lemon peel	5.00	25	5.0	81.61	[21]
Citric acid-modified grape pulp	4.80	25	10.0	114.88	[22]
Biochar supported sulfide nanoscale zero-valent iron	5.00	25	0.2	103.20	[23]
Pomelo peel biochar/MgFe-LDH	5.50	45	1.5	448.96	[24]
Rice hush ash	6.00	30	4.0	3.04	[25]

NaOH treated rice husk	6.80	28	10.0	20.24	[26]
Bagasse fly ash	6.00	30	10.0	6.19	[27]
Grape stalk wastes	5.20	20	6.67	24.39	[28]
Corncob	8.00	25	2.0	5.12	[29]
Areca waste	5.60	20	10.0	3.73	[30]
Banana peel biochar /Fe_3O_4/ZIF-67	6.00	25	1.0	50.78	[31]
Spirodela polyrhiza	6.00	20	1.0	36.00	[32]
MgO-modified sludge biochar	6.00	25	0.5	219.06	[33]
Fe–Mn oxide-modified biochar	5.00	22	2.0	119.04	[34]
Sulfur-functionalized rice husk	7.00	25	0.1	137.16	[35]
Calcium thioglycolate-modified straw biochar	6.00	25	0.8	65.44	[36]
Spinach waste-derived biochar	6.00	25	1.0	186.04	[37]
S. europaea biochar	6.00	25	0.67	108.54	[38]
Sarsagum sp.	5.00	25	1.0	79.40	[39]
Lactarius scrobiculatus	5.50	20	4.0	53.10	[40]
Phanerochaete chrysosporium	4.50	27	2.0	15.20	[41]
Bifurcaria bifurcata	4.50	25	2.5	74.00	[42]
Gracillaria sp.	5.50	22	1.0	33.72	[43]
**Polymer adsorbents**					
CS-LDH	6.00	25	2.5	140.80	[44]
PSt@p(NIPMAM-Aac)	6.00	25	0.11	476.20	[45]
Porous resin	7.00	25	20.0	3.506	[46]
0.3Ma-MgMnLDO-a	5.00	30	0.2	422.60	[47]
Fe_3O_4/PMA-g-PVA	7.00	25	2.0	94.75	[48]
NH_2-Zr-MOFs	6.00	30	0.2	177.35	[49]
Chitosan/PVA	6.00	50	10.0	142.90	[50]
Polyacrylamide-grafted iron(III) oxide	6.00	30	0.2	147.20	[51]
Guar gum-graft-poly(ethylacrylate)	9.00	30	2.5	714.28	[52]
Polypyrrole-grafted-chitin	6.00	50	2.0	6.49	[53]

Chitosan	5.00	20	0.5	105.26	[54]
Methylphosphonic Acid grafted polystyrene resin	5.00	25	6.0	37.90	[55]
TEMPO-oxidized cellulose	7.00	25	5.0	5.83	[56]
**Nano-composite materials adsorbents**					
N-doped hierarchically porous carbon	5.00	25	1.0	43.50	[57]
PVA/NaX nanofibers	5.00	45	0.5	838.70	[58]
Nano-composite materials	5.50	25	0.33	148.32	[59]
Sodium titanate nano fibrous material (TNF)	6.50	25	0.5	149.00	[60]
$Bi_2Co_3O_7$@g-C_3N_4 nanocomposite	5.00	25	0.4	216.24	[61]
MgO@TiO_2@g-C_3N_4	7.00	25	0.4	529.00	[62]
Y_2O_3@gC_3N_4 nanocomposite	5.00	25	0.4	45.66	[63]
Eggshell/starch/Fe_3O_4 magnetic nanocomposites	7.00	25	1.0	48.54	[64]
**Minerals adsorbents**					
Perlite	6.00	33	8.0	0.64	[65]
FeMg-LDH@bentonite	7.00	25	0.25	510.20	[66]
Expanded perlite	6.00	20	10.0	1.79	[67]
Fluorapatite	4.00	25	0.5	115.36	[68]
Granular red mud	6.50	50	100.0	10.79	[69]
Natural bentonite	5.00	25	1.0	61.35	[70]
Orthophosphate-modified kaolinite clay	6.00	28	50.0	15.60	[71]
Microporous titanosilicate ETS-10	5.00	25	5.0	40.92	[72]
Jordanian natural zeolite	6.40	25	5.0	4.20	[73]
Limonitic laterite	7.00	25	2.0	46.09	[74]
Biogenic hydroxyapatite	5.00	25	1.25	92.54	[75]
Low-grade phosphorus-containing oolitic hematite	6.10	50	20.0	55.37	[76]
**Metal oxides or metal adsorbents**					

Surfactant-modified alumina (SMA)	6.00	22	4.0	24.40	[77]
FeS	6.00	25	0.1	116.00	[78]
Flowerlike MgO nanostructures	7.00	25	0.67	1500	[79]
Nanoscale zero-valent iron (nZVI)	8.60	25	5.0	66.90	[80]
Mesoporous silica	4.50	25	2.0	3.62	[81]
MnO_2-coated Fe_3O_4 nanocomposites	6.30	25	1.0	53.20	[82]
EDTAD-treated FB	6.00	10	1.0	41.00	[83]
Fe_3O_4@APS@AA-co-CA	5.50	25	1.0	29.60	[84]
MAMNPs	6.00	25	1.5	91.55	[85]
α-Fe_2O_3 nanoparticles-coated volcanic rock	6.00	40	1.0	158.48	[86]
Nanoscale zero-valent iron	6.00	25	0.5	48.63	[87]
Fe_3O_4/HA	6.00	20	0.1	50.40	[88]
Ca-S ferromanganese binary oxide	6.00	25	0.2	107.53	[89]
Magnetite nanorods	5.50	25	1.0	88.39	[90]
Hydrous manganese dioxide (HMO)	4.00	25	0.5	140.40	[91]
**Gel adsorbents**					
Multi-carboxyl-functionalized silica gel	5.00	25	2.0	43.84	[92]
PS-GO gel	6.00	40	3.0	136.98	[93]
NTA-silica gel	5.00	25	1.0	53.14	[94]
Chitin/chitosan-yeast hybrid aerogel	6.00	35	1.25	127.50	[95]
EDTA-CS-PAM hydrogel	5.00	25	1.0	85.00	[96]
Chitosan/alginate-based hydrogel	5.00	23	1.0	216.50	[97]
βCD-CMC hydrogel	6.00	25	3.2	41.84	[98]
**Graphene and graphene oxide adsorbents**					
Magnetic GO/MgAl-LDH	6.00	25	3.5	45.05	[99]
NZVI/rGOs	5.00	60	0.1	513.92	[100]
3D sulfonated reduced graphene oxide	6.00	25	0.1	234.80	[101]

Graphene oxide nanosheets	6.00	30	0.1	106.30	[102]
Graphene oxide /CNTs Membranes	5.00	25	0.045	48.0	[103]
Graphene oxide aerogel	6.00	30	1.0	108.70	[104]
Chitosan/sulfydryl-functionalized graphene oxide	5.00	20	0.2	177.00	[105]
DTPA/MGO composites	3.00	20	0.4	286.50	[106]
GO/orange peel/chitosan nanocomposite	6.00	25	0.05	537.63	[107]
**Carbon nanotubes adsorbents**					
MWCNT	5.00	25	0.5	10.86	[108]
MnO_2/o-MWCNTs	5.00	25	0.5	41.60	[109]
EDA-functionalized MWCNTs	8.00	45	0.1	25.70	[110]
Aalumina-CNT nanoclusters	7.50	30	2.5	229.90	[111]
CNT sheets	7.00	25	2.0	92.59	[112]
Oxidized CNT	6.50	25	0.5	128.50	[113]

Table S5. Kinetics fitting parameters for Cd(II) adsorption on RF-1.25BFA and RF-1.25BFA-540 at different temperatures (T = 5 °C, 15 °C, 25 °C, 35 °C) and pH 6.50.

Adsorbents	T (°C)	Pseudo-first-order model			
		$q_{e,cal}$a	$q_{e,exp}$b	K_1 (min^{-1})	R^2
RF-1.25BFA	5	390.60	394.48	3.51	0.9986
	15	392.64	397.64	3.59	0.9983
	25	399.73	400.00	5.01	0.9999
	35	399.86	400.00	5.83	0.9999
RF-1.25BFA-540	5	387.83	392.32	3.23	0.9984
	15	390.92	396.28	3.08	0.9983
	25	395.88	400.00	2.89	0.9979
	35	396.61	400.00	3.04	0.9986
**Adsorbents**	**T (°C)**	**Pseudo-second-order model**			
		$q_{e,cal}$a	$q_{e,exp}$b	K_2 (g·mg^{-1}·min^{-1})	R^2

RF-1.25BFA	5	392.07	394.48	0.052	0.9990
	15	394.20	397.64	0.051	0.9986
	25	400.12	400.00	0.210	0.9999
	35	400.38	400.00	0.216	0.9999
RF-1.25BFA-540	5	388.95	392.32	0.050	0.9990
	15	391.78	396.28	0.050	0.9990
	25	396.28	400.00	0.051	0.9986
	35	397.31	400.00	0.051	0.9992

Adsorbents	T (°C)	Elovich model		
		A	B	R^2
RF-1.25BFA	5	378.82	2.72	0.9999
	15	380.62	2.81	0.9998
	25	397.91	0.40	0.9999
	35	399.02	0.18	0.9999
RF-1.25BFA-540	5	373.83	3.20	0.9999
	15	375.29	3.55	0.9998
	25	377.91	4.05	0.9994
	35	381.37	3.43	0.9996

a The calculated adsorption capacity at equilibrium, namely q_e in eq 3 and eq 4.

b The measured adsorption capacity at equilibrium.

Table S6. Parameters of adsorption thermodynamic for Cd(II) adsorption on RF-1.25BFA and RF-1.25BFA-540.

Adsorbents	T (K)	ΔG^θ (kJ/mol)	ΔH^θ (kJ/mol)	ΔS^θ (kJ·mol^{-1}·K^{-1})
RF-1.25BFA	278	−9.87	57.075	0.241
	288	−12.28		
	298	−28.52		
	308	−29.48		
RF-1.25BFA-540	278	−9.09	48.928	0.209
	288	−11.18		

	298	−28.52
	308	−29.48

Table S7. Kinetics fitting parameters for Cd(II) adsorption on RF-1.25BFA and RF-1.25BFA-540 at different pH values (pH = 3.30, 5.00, 6.50, 7.60, 8.50) and 25 °C.

Adsorbents	pH	Pseudo-first-order model			
		$q_{e,cal}$a	$q_{e,exp}$b	K_1 (min^{-1})	R^2
RF-1.25BFA	3.30	234.14	247.28	1.67	0.9474
	5.00	383.48	391.36	2.96	0.9950
	6.50	399.73	400.00	5.01	0.9999
	7.60	399.84	400.00	5.73	0.9999
	8.50	399.86	400.00	5.83	0.9999
RF-1.25BFA-540	3.30	198.12	214.96	1.09	0.8908
	5.00	380.68	389.08	2.89	0.9946
	6.50	395.88	400.00	2.89	0.9979
	7.60	396.67	400.00	3.04	0.9985
	8.50	397.14	400.00	3.06	0.9988
Adsorbents	pH	Pseudo-second-order model			
		$q_{e,cal}$a	$q_{e,exp}$b	K_2 ($g \cdot mg^{-1} \cdot min^{-1}$)	R^2
RF-1.25BFA	3.30	240.21	247.28	0.013	0.9663
	5.00	384.15	391.36	0.052	0.9962
	6.50	400.12	400.00	0.210	0.9999
	7.60	400.35	400.00	0.212	0.9999
	8.50	400.38	400.00	0.214	0.9999
RF-1.25BFA-540	3.30	205.97	214.96	0.006	0.9468
	5.00	381.38	389.08	0.047	0.9959
	6.50	396.28	400.00	0.051	0.9986
	7.60	397.32	400.00	0.052	0.9991
	8.50	397.71	400.00	0.055	0.9993
Adsorbents	pH	Elovich model			

		A	B	R^2
RF-1.25BFA	3.30	190.12	10.18	0.9921
	5.00	362.33	4.92	0.9995
	6.50	397.91	0.40	0.9999
	7.60	398.91	0.21	0.9999
	8.50	399.02	0.18	0.9999
RF-1.25BFA-540	3.30	137.48	13.86	0.9834
	5.00	358.49	5.16	0.9995
	6.50	377.91	4.05	0.9994
	7.60	381.43	3.43	0.9995
	8.50	382.84	3.20	0.9995

a The calculated adsorption capacity at equilibrium, namely q_e in eq 3 and eq 4.

b The measured adsorption capacity at equilibrium.

Table S8. The pH values change of the Cd(II) adsorption by RF-1.25BFA and RF-1.25BFA-540 under different pH conditions after equilibrium.

Initial pH	Equilibrium pH	
	RF-1.25BFA	RF-1.25BFA-540
3.30 ± 0.01	3.44 ± 0.12	3.52 ± 0.15
5.00 ± 0.01	5.48 ± 0.15	5.80 ± 0.14
6.50 ± 0.01	5.97 ± 0.11	6.26 ± 0.12
7.60 ± 0.01	6.30 ± 0.13	6.37 ± 0.10
8.50 ± 0.01	6.48 ± 0.13	6.56 ± 0.14

Table S9. Adsorption energy of Cd(II) by different optimized geometries in DFT calculations.

Optimized geometries	Adsorption energy (ΔE_{ad}, eV)
C–ad1	−1.714
C–ad2	−1.561
C–ad3	−1.607

Iron clusters–ad1	−6.404
Iron clusters–ad2	−4.867
Iron clusters–ad3	−4.539

Table S10. Comparison of the performances and economic analysis of different adsorbents.

Adsorbents	Heavy metal	Cycle number	Specific surface area (m^2/g)	Contact time (min)	Chemical cost of adsorption ($/mg)	Adsorption capacity (mg/g)	Adsorbent dosage (g/L)	Refs.
RF-1.25BFA	Cd(II)	15	199.68	10	0.0179	1108.87	0.025	This work
ZIF-8	Cu(II)	3	798.00	30	0.303	98.60	1.00	[114]
UiO-66s@NAP	Cu(II)	4	1126.00	60	0.353	164.13	0.10	[115]
UCN-GH	Cu(II)	3	24.25	120	4.180	127.10	2.00	[116]
C-nZVI-BC	Cr(VI)	6	883.10	240	0.0456	82.80	1.00	[117]
nHAP@biochar	Cu(II)	4	566.10	600	0.564	44.62	0.125	[118]
CPMB	Cu(II)	0	62.60	240	0.209	70.28	1.00	[119]
BAC@SiO_2-EDTA	Pb(II)	5	69.92	60	0.2068	123.45	0.80	[120]
Modified biochar	Cr(VI)	0	18.698	100	1.032	45.88	2.00	[121]
CMt	Cr(VI)	0	36.00	600	3.203	100.00	1.25	[122]
MBC	Cd(II)	3	459.00	2	1.445	11.30	0.40	[123]
Bacterial biochar	Cr(VI)	5	22.09	600	0.392	29.73	1.00	[124]

Supplementary figure

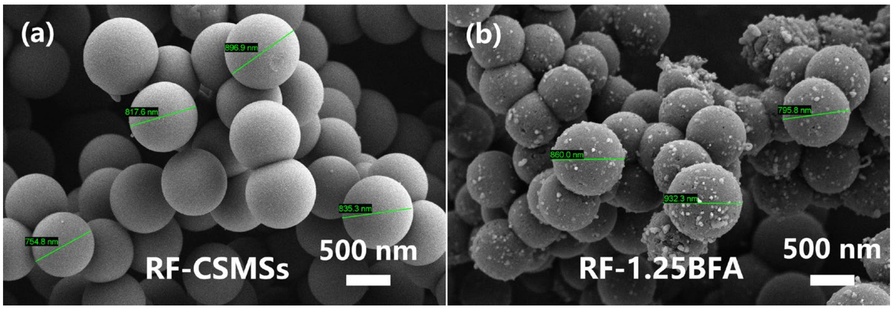

Figure S1. FE-SEM images with size scale of (a) RF-CSMSs and (b) RF-1.25BFA.

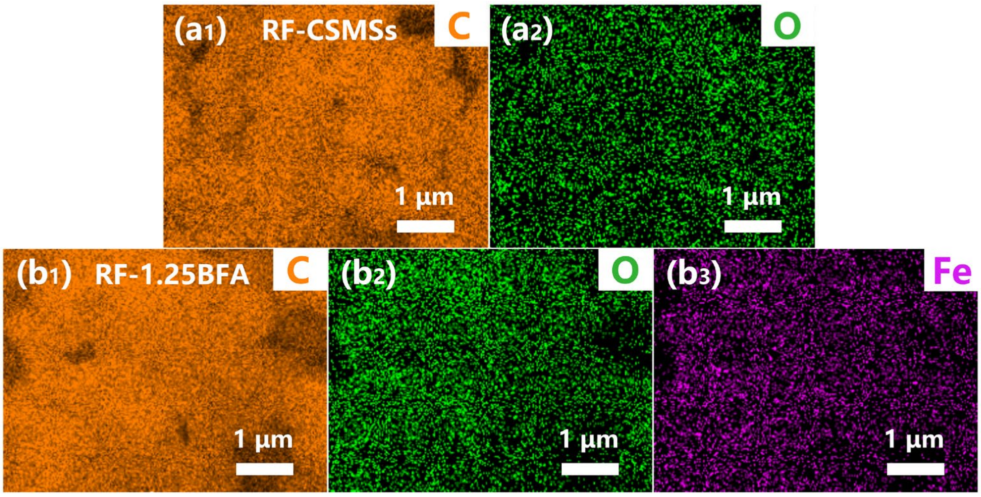

Figure S2. EDS elemental mappings of (a_1) C element, (a_2) O element of the RF-CSMSs; (b_1) C element, (b_2) O element and (b_3) Fe element of the RF-1.25BFA.

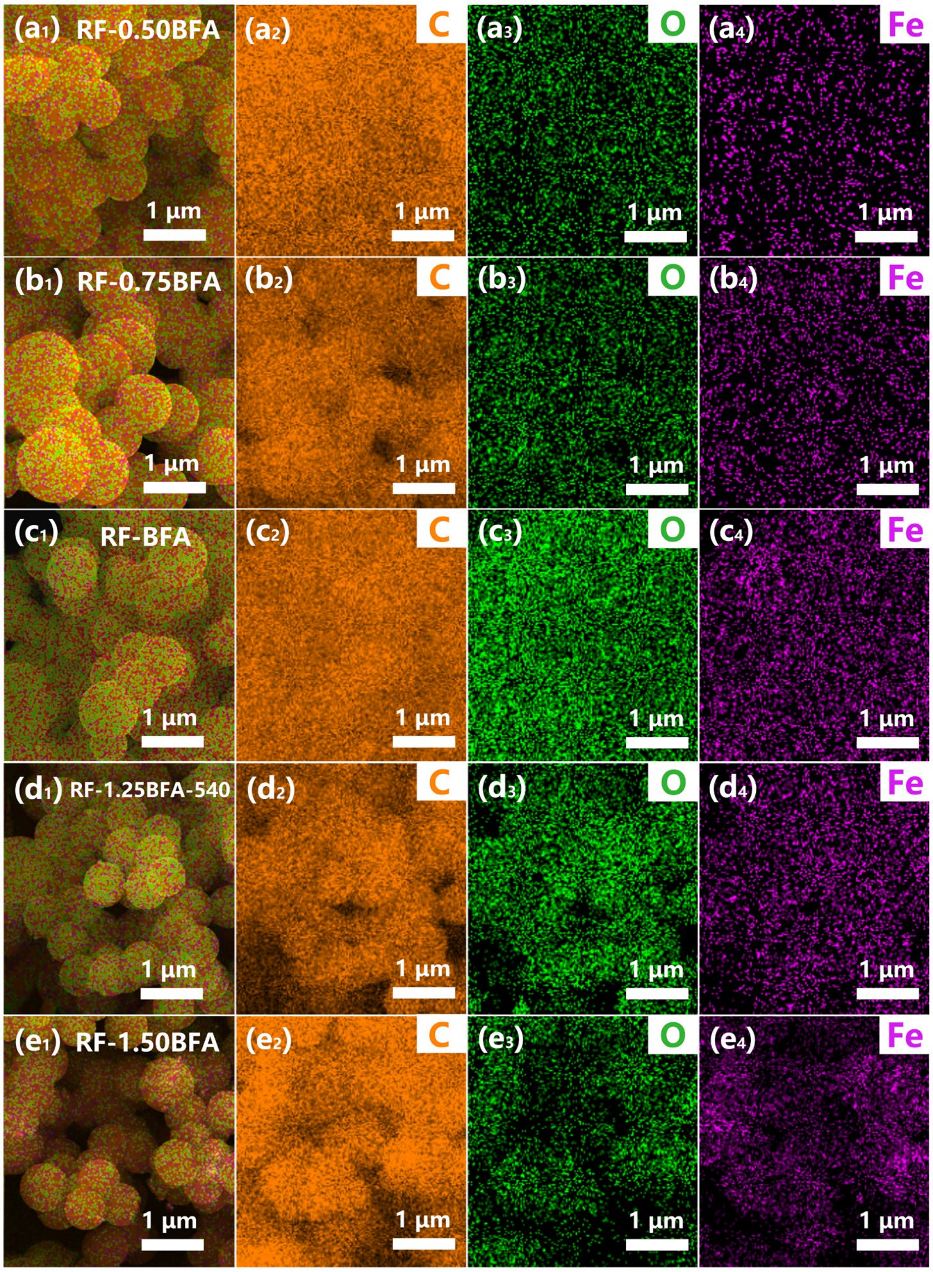

Figure S3. EDS elemental mappings of C, O and Fe elements of (a_1–a_4) RF-0.50BFA, (b_1–b_4) RF-0.75BFA, (c_1–c_4) RF-BFA, (d_1–d_4) RF-1.25BFA-540 and (e_1–e_4) RF-1.50BFA.

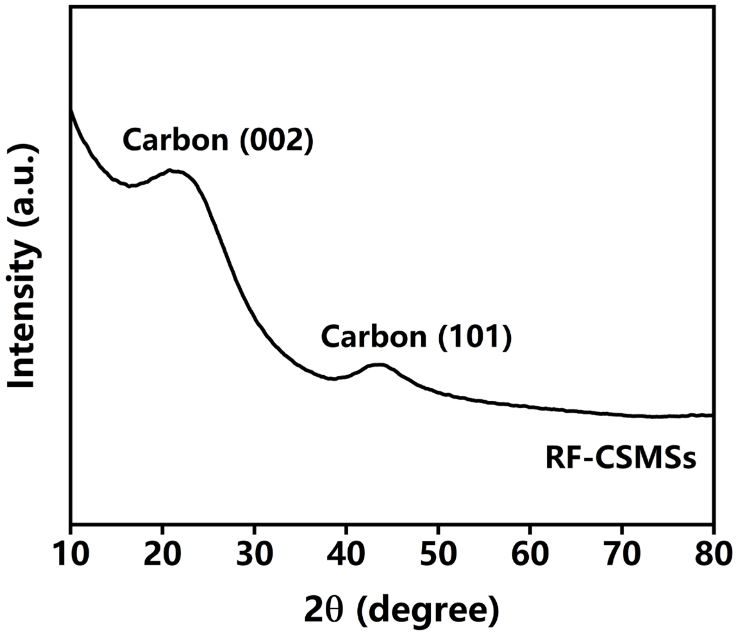

Figure S4. XRD pattern of RF-CSMSs.

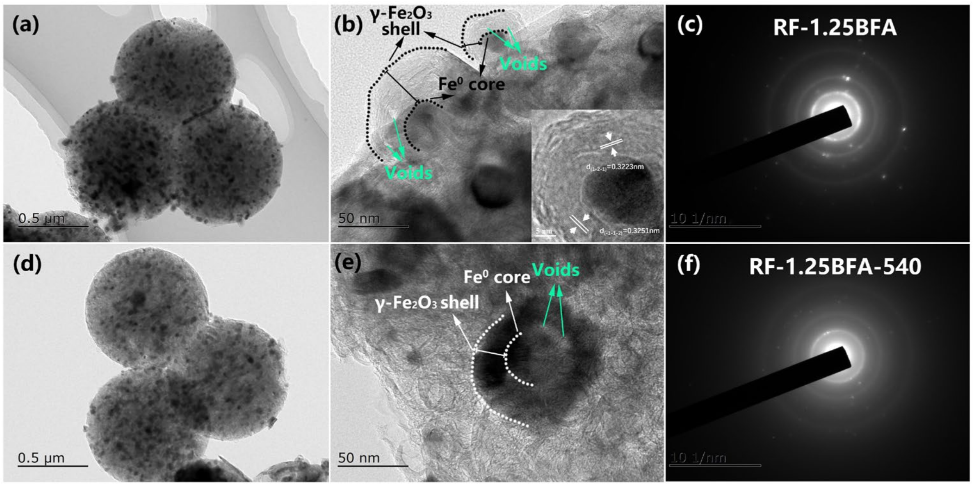

Figure S5. TEM images (a, b) RF-1.25BFA and (d, e) RF-1.25BFA-540. Selected-area electron diffraction (SAED) patterns (c) RF-1.25BFA and (f) RF-1.25BFA-540.

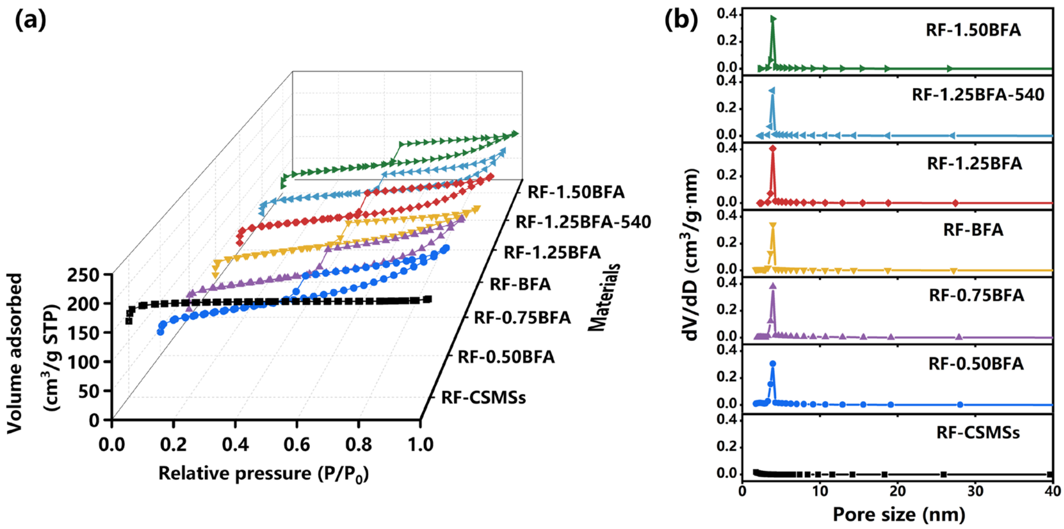

Figure S6. (a) N_2 absorption–desorption isotherms and (b) pore size distribution curves of the as-prepared CSMCs.

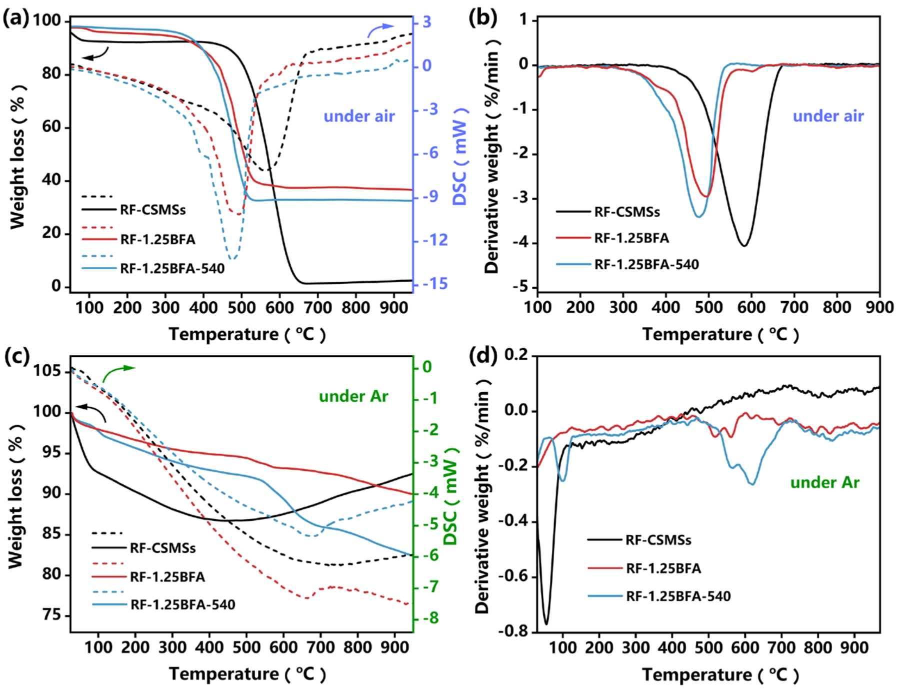

Figure S7. The thermogravimetric characterization of RF-CSMSs, RF-1.25BFA and RF-1.25BFA-540 under air (a, b) and argon (c, d).

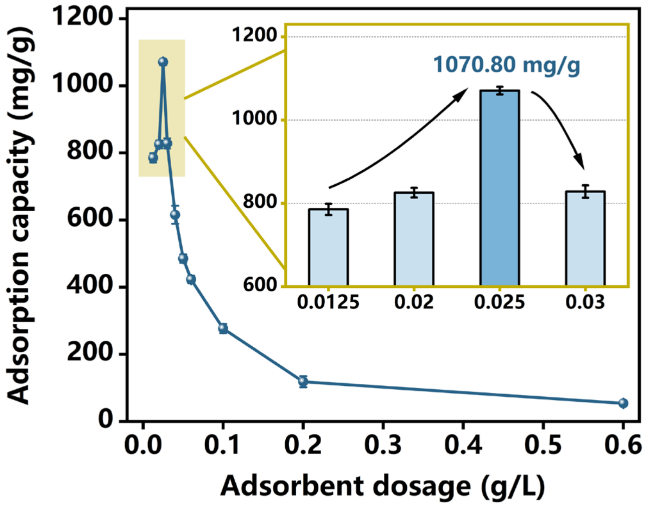

Figure S8. Effect of adsorbent dosage on the adsorption capacity of RF-1.25BFA. Conditions: [Initial Cd(II)] =100 mg/L, adsorbent dosage = 0.0125 g/L, 0.02 g/L, 0.025 g/L, 0.03 g/L, 0.04 g/L, 0.05 g/L, 0.06 g/L, 0.1 g/L, 0.2 g/L and 0.6 g/L, T = 25 °C, pH = 6.50 and equilibrium time = 720 min.

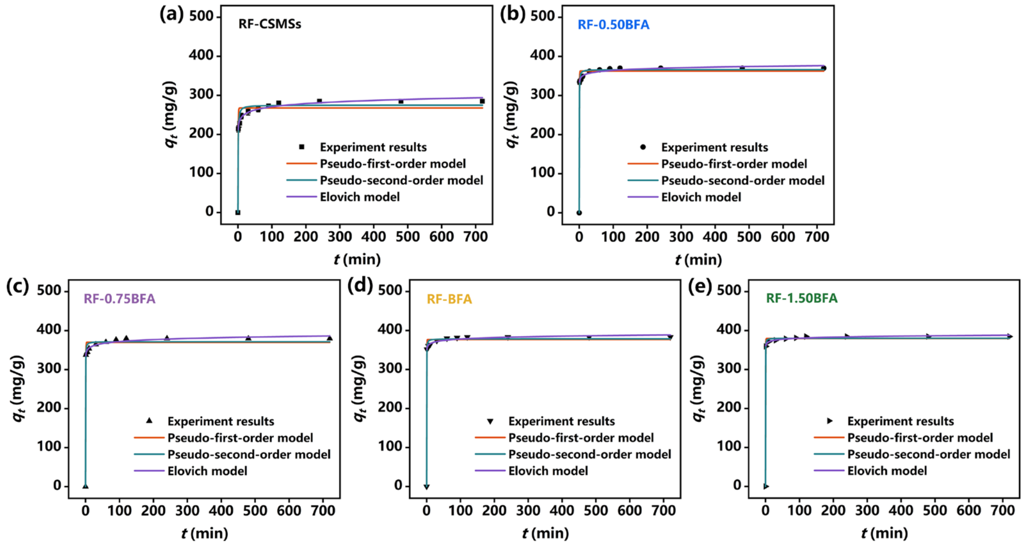

Figure S9. Adsorption kinetics for Cd(II) adsorption on (a) RF-CSMSs, (b) RF-0.50BFA, (c) RF-0.75BFA, (d) RF-BFA and (e) RF-1.50BFA: the pseudo-first-order, pseudo-second-order and Elovich equation model fitting curves. Conditions: [Initial Cd(II)] =10 mg/L, adsorbent dosage = 0.025 g/L, T = 25 °C, pH = 6.50 and equilibrium time = 720 min.

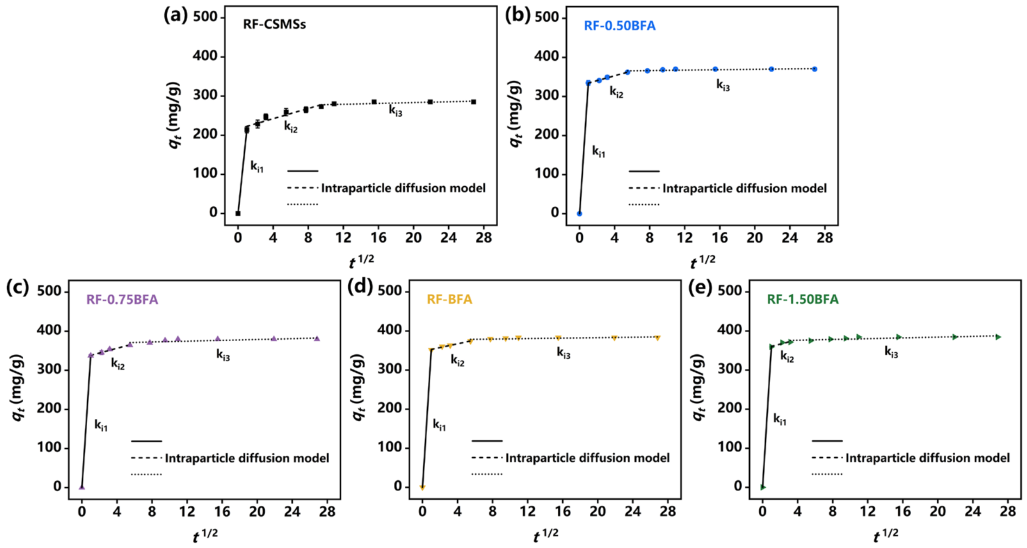

Figure S10. Intraparticle diffusion model fitting curve for Cd(II) adsorption on (a) RF-CSMSs, (b) RF-0.50BFA, (c) RF-0.75BFA, (d) RF-BFA and (e) RF-1.50BFA. Conditions: [Initial Cd(II)] =10 mg/L, adsorbent dosage = 0.025 g/L, T = 25 °C, pH = 6.50 and equilibrium time = 720 min.

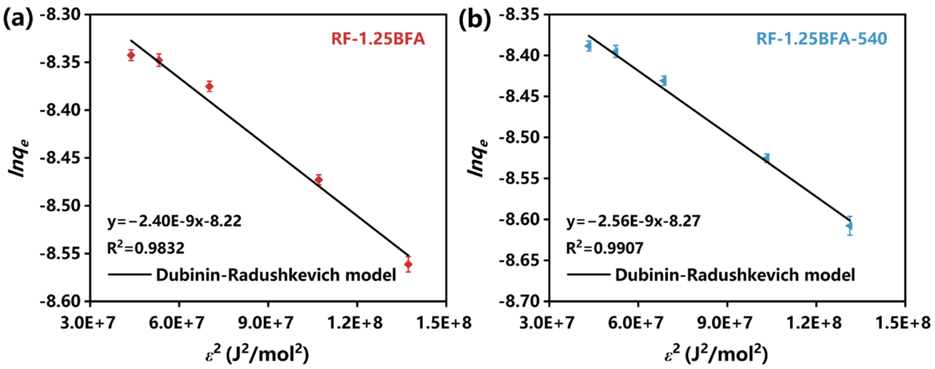

Figure S11. Dubinin–Radushkevich (D-R) isotherm plots obtained for Cd(II) adsorption on (a) RF-1.25BFA and (b) RF-1.25BFA-540. Conditions: [Initial Cd(II)] = 5–300 mg/L, adsorbent dosage = 0.025 g/L, T = 25 °C, pH = 6.50 and equilibrium time = 720 min.

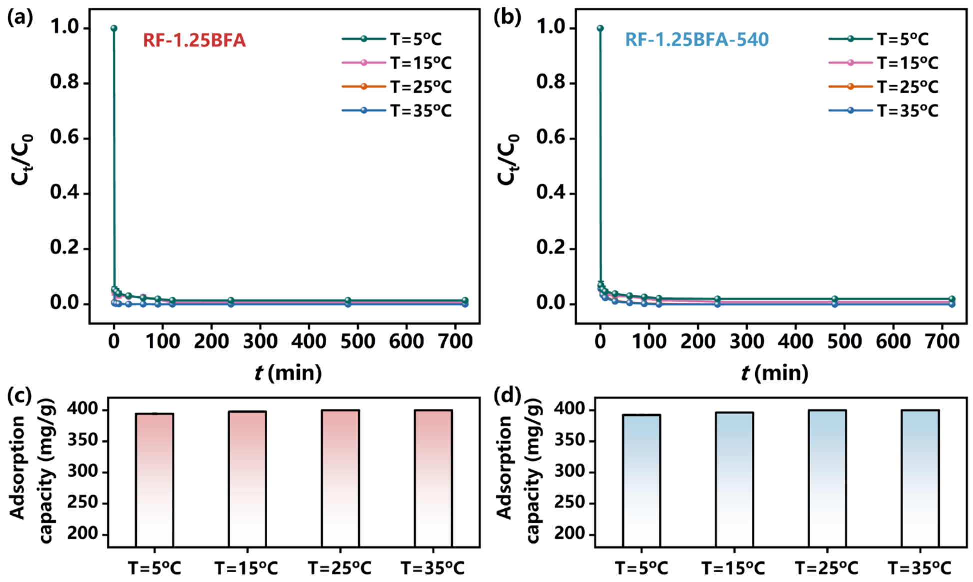

Figure S12. Effect of temperature towards adsorption performance of (a) RF-1.25BFA and (b) RF-1.25BFA-540 for Cd(II). Adsorption capacity of (c) RF-1.25BFA and (d) RF-1.25BFA-540 at different temperature. Conditions: [Initial Cd(II)] =10 mg/L, adsorbent dosage = 0.025 g/L, T = 5 °C, 15 °C, 25 °C, 35 °C, pH = 6.50 and equilibrium time = 720 min.

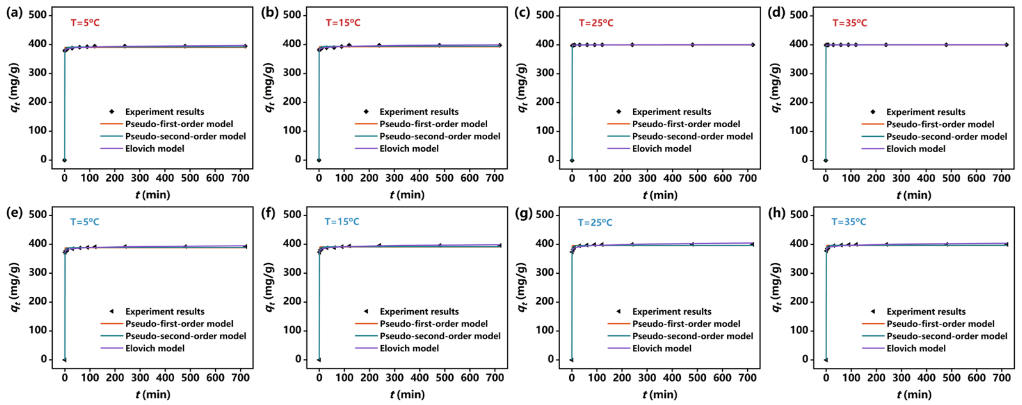

Figure S13. Effect of temperature towards adsorption kinetics for Cd(II) adsorption on (a–d) RF-1.25BFA and (e–h) RF-1.25BFA-540: the pseudo-first-order, pseudo-second-order and Elovich equation model fitting curves. Conditions: [Initial Cd(II)] =10 mg/L, adsorbent dosage = 0.025 g/L, T = 5 °C, 15 °C, 25 °C, 35 °C, pH = 6.50 and equilibrium time = 720 min.

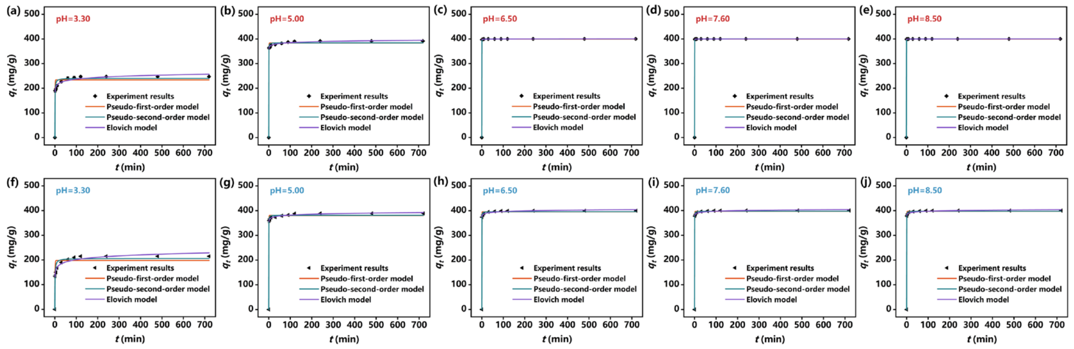

Figure S14. Effect of pH towards adsorption kinetics for Cd(II) adsorption on (a–e) RF-1.25BFA and (f–j) RF-1.25BFA-540: the pseudo-first-order, pseudo-second-order and Elovich equation model fitting curves. Conditions: [Initial Cd(II)] =10 mg/L, adsorbent dosage = 0.025 g/L, T = 25 °C, pH = 3.30, 5.00, 6.50, 7.60, 8.50 and equilibrium time = 720 min.

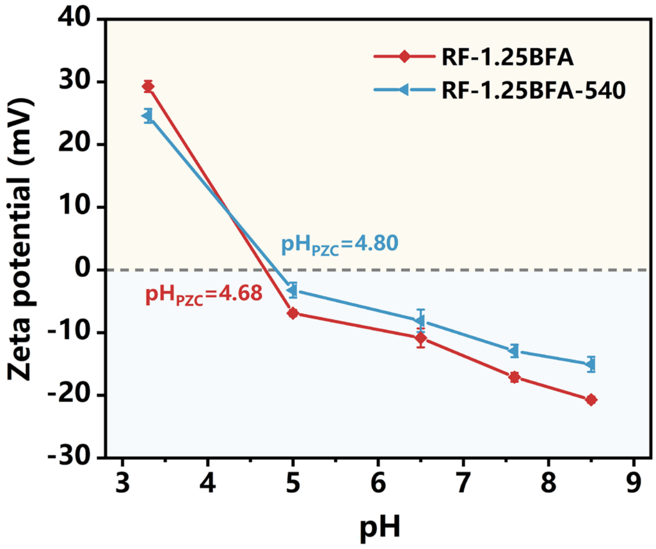

Figure S15. Zeta potential curves of RF-1.25BFA and RF-1.25BFA-540.

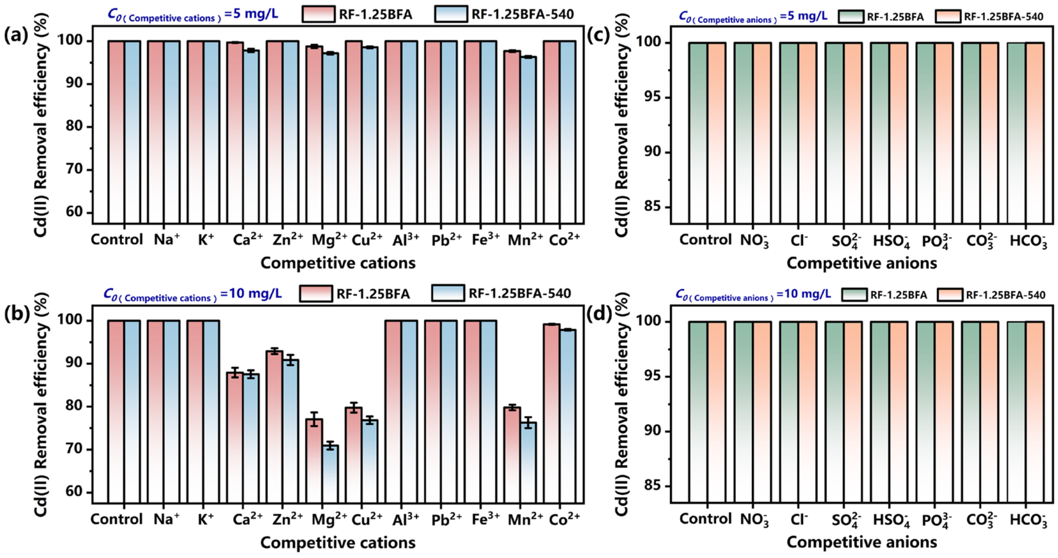

Figure S16. Effect of different concentrations (5 and 10 mg/L) of competitive (a, b) cations and (c, d) anions for Cd(II) adsorption on RF-1.25BFA and RF-1.25BFA-540. Conditions: [Initial Cd(II)] =10 mg/L, adsorbent dosage = 0.025 g/L, T = 25 °C, pH = 6.50 and equilibrium time = 720 min.

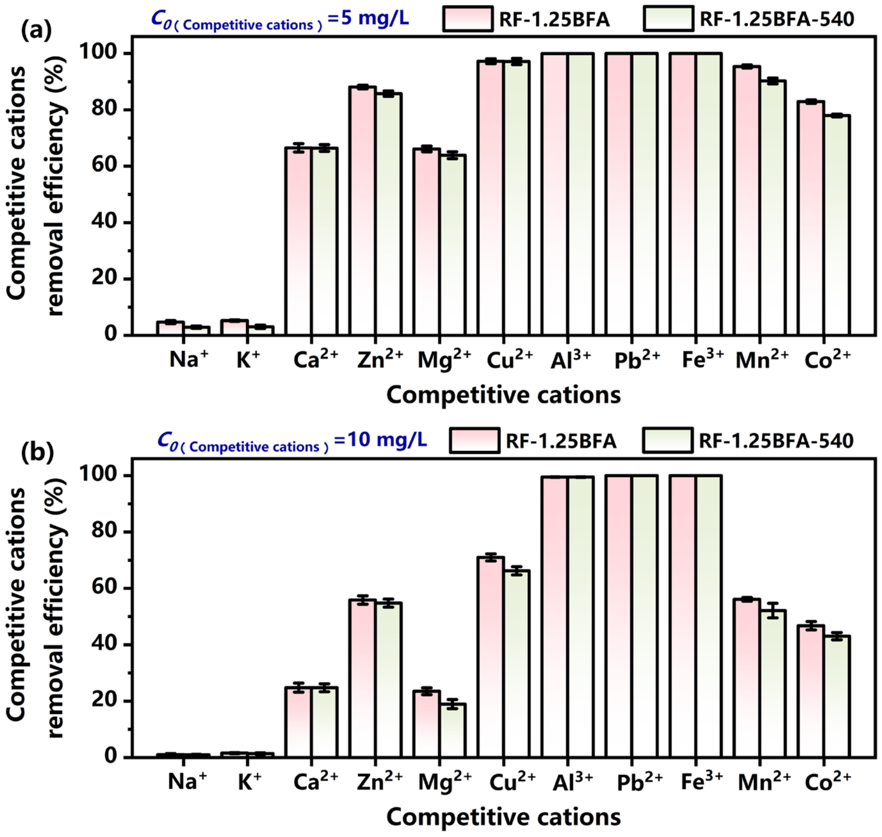

Figure S17. Removal efficiency of different concentrations (a) 5 mg/L and (b) 10 mg/L of competitive cations in competitive systems. Conditions: [Initial Cd(II)] =10 mg/L, adsorbent dosage = 0.025 g/L, T = 25 °C, pH = 6.50 and equilibrium time = 720 min.

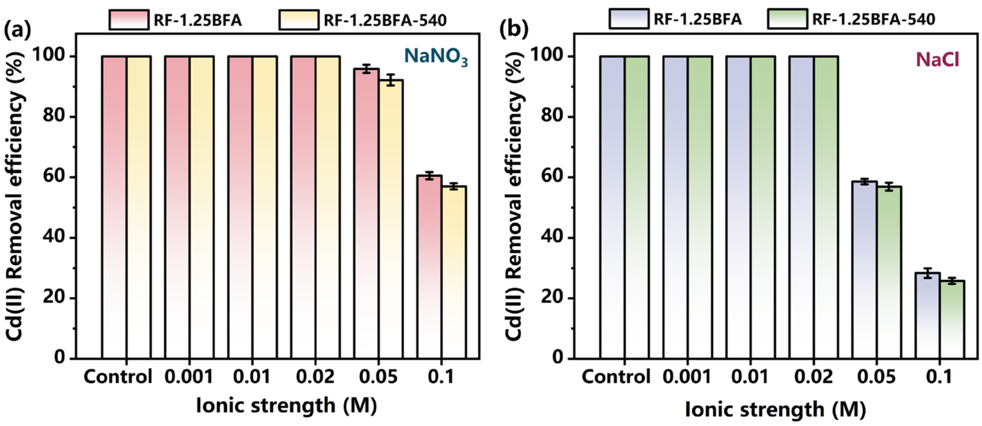

Figure S18. Effect of solution ionic strength and electrolyte type (a) NaCl and (b) $NaNO_3$ for Cd(II) adsorption on RF-1.25BFA and RF-1.25BFA-540. Conditions: [Initial Cd(II)] =10 mg/L, adsorbent dosage = 0.025 g/L, T = 25 °C, pH = 6.50 and equilibrium time = 720 min.

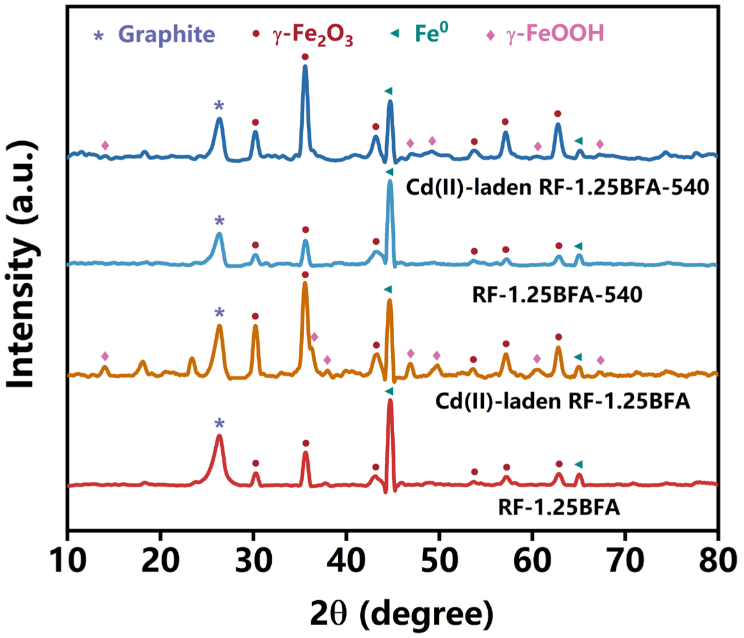

Figure S19. XRD patterns of RF-1.25BFA and RF-1.25BFA-540 after Cd(II) adsorption.

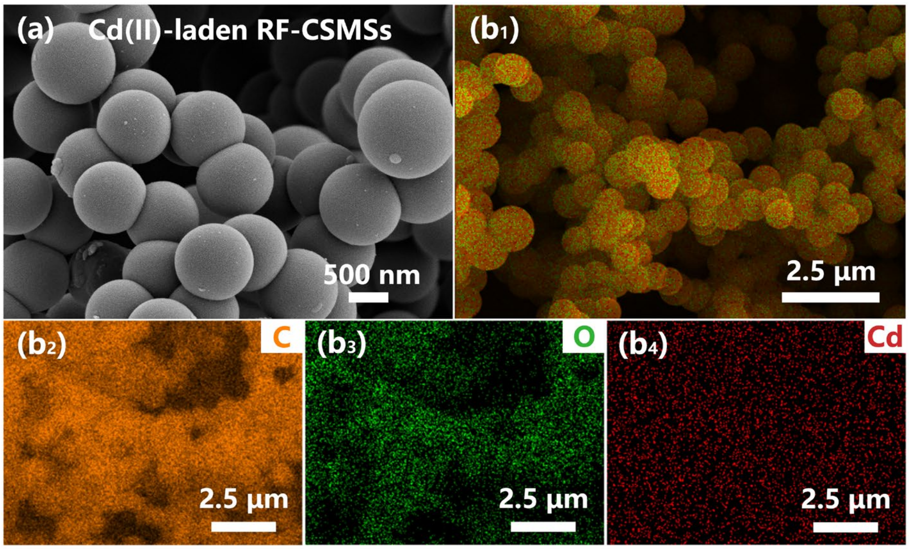

Figure S20. FE-SEM image and EDS elemental mappings of RF-CSMSs after Cd(II) adsorption.

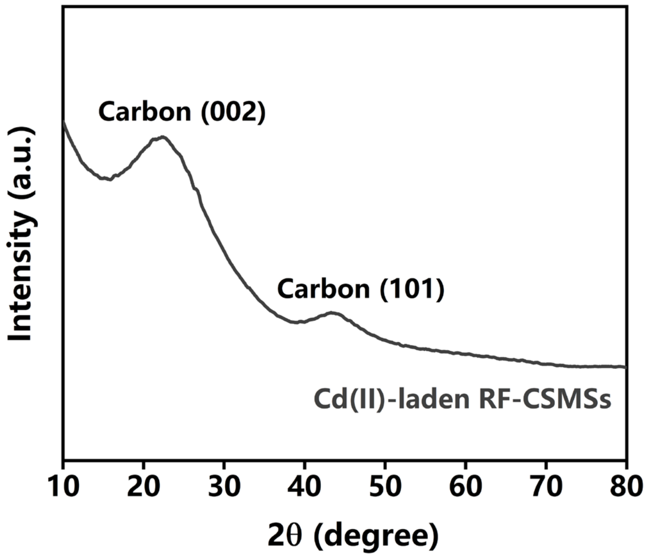

Figure S21. XRD pattern of RF-CSMSs after Cd(II) adsorption.

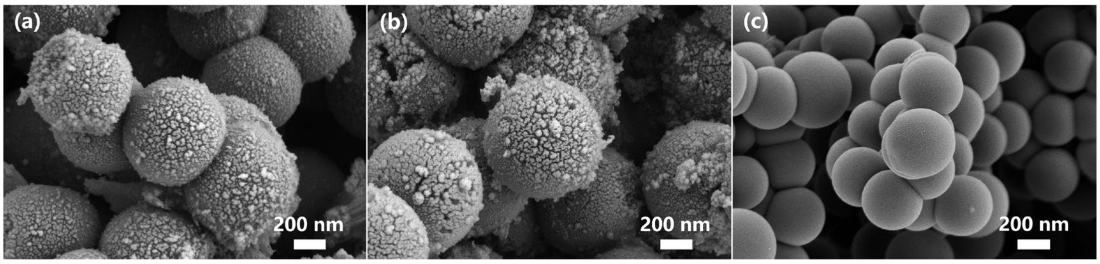

Figure S22. FE-SEM images of (a) RF-1.25BFA, (b) RF-1.25BFA-540 and (c) RF-CSMSs after the same adsorption process in the liquid phase without Cd(II).

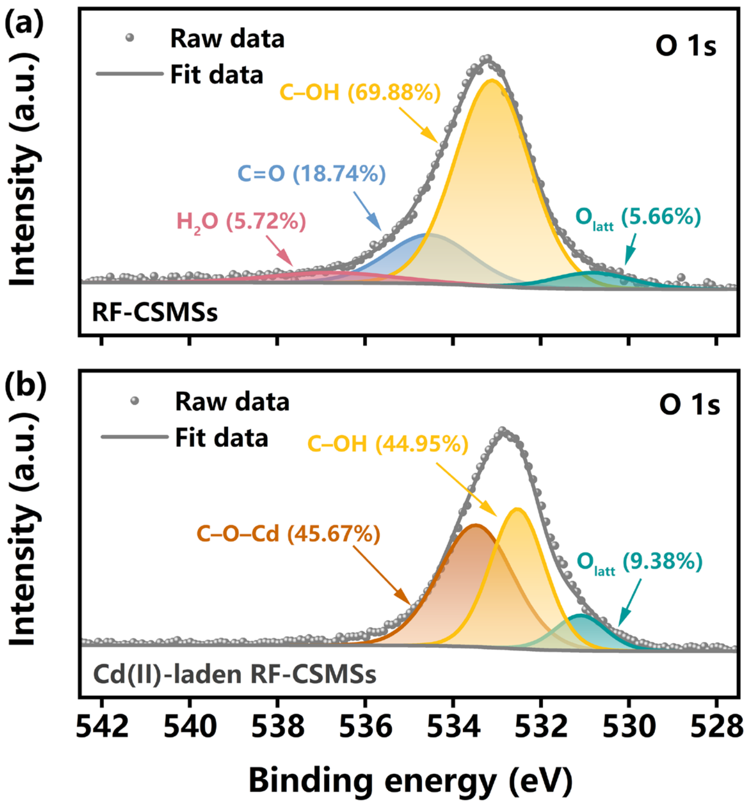

Figure S23. The high-resolution XPS spectra of O 1 s in (a) RF-CSMSs and (b) Cd(II)-laden RF-CSMSs.

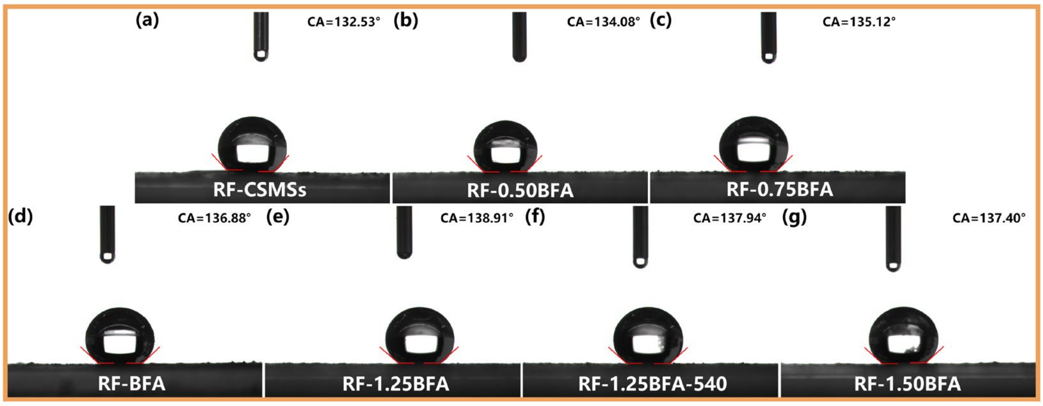

Figure S24. Wetting behavior of (a) RF-CSMSs, (b) RF-0.50BFA, (c) RF-0.75BFA, (d) RF-BFA, (e) RF-1.25BFA, (f) RF-1.25BFA-540 and (g) RF-1.50BFA toward water in air.

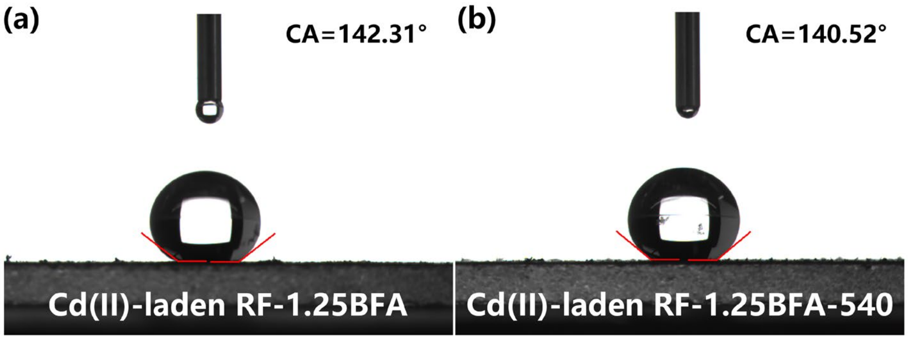

Figure S25. Wetting behavior of (a) RF-1.25BFA and (b) RF-1.25BFA-540 after Cd(II) adsorption toward water in air.

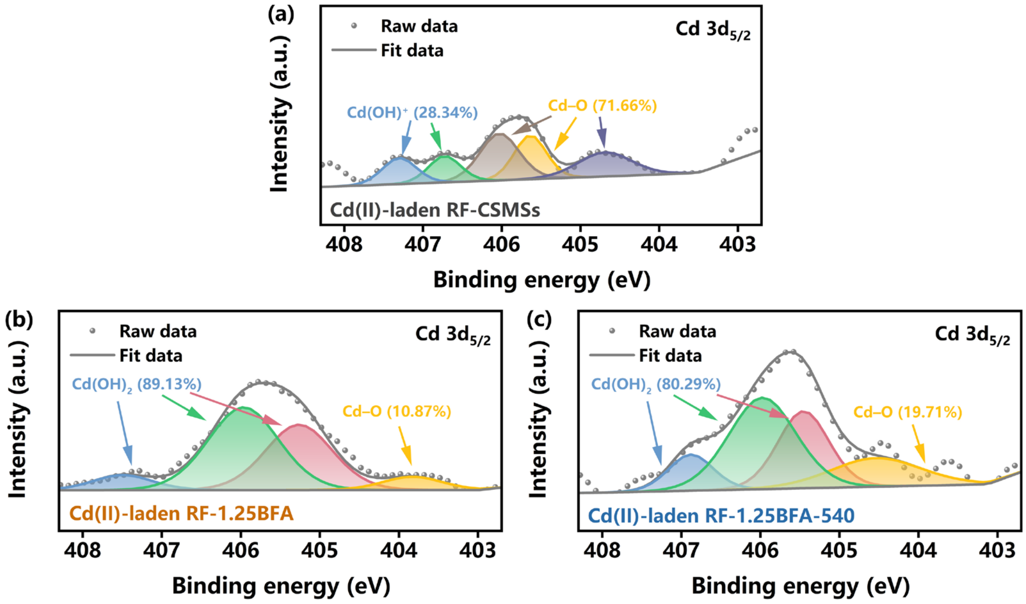

Figure S26. The high-resolution XPS spectra of Cd 3 $d_{5/2}$ in (a) Cd(II)-laden RF-CSMSs, (b) Cd(II)-laden RF-1.25BFA and (c) Cd(II)-laden RF-1.25BFA-540.

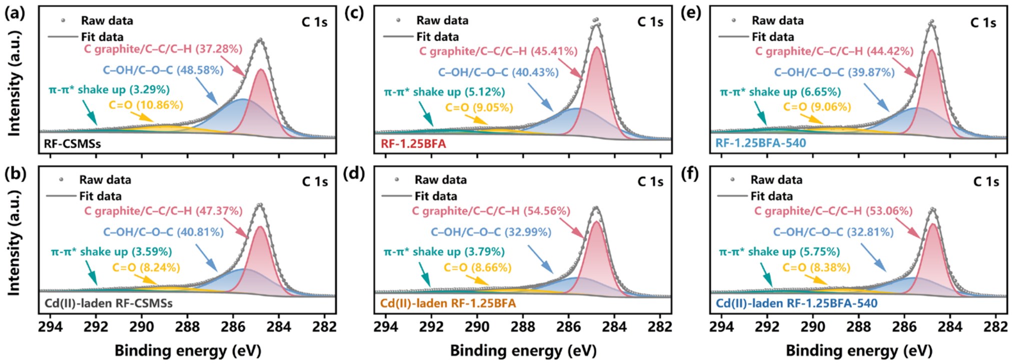

Figure S27. The high-resolution XPS spectra of C 1 s in (a) RF-CSMSs, (b) Cd(II)-laden RF-CSMSs, (c) RF-1.25BFA, (d) Cd(II)-laden RF-1.25BFA, (e) RF-1.25BFA-540 and (f) Cd(II)-laden RF-1.25BFA-540.

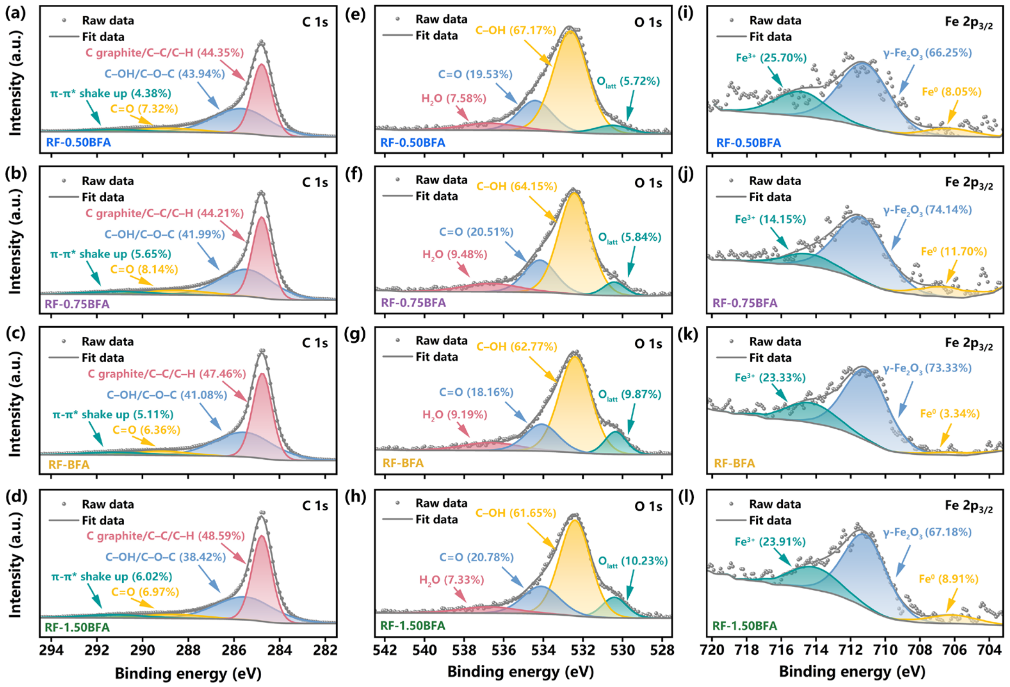

Figure S28. The high-resolution XPS spectra of (a–d) C 1 s, (e–h) O 1 s and (i–l) Fe 2 $p_{3/2}$ in RF-0.50BFA, RF-0.75BFA, RF-BFA and RF-1.50BFA.

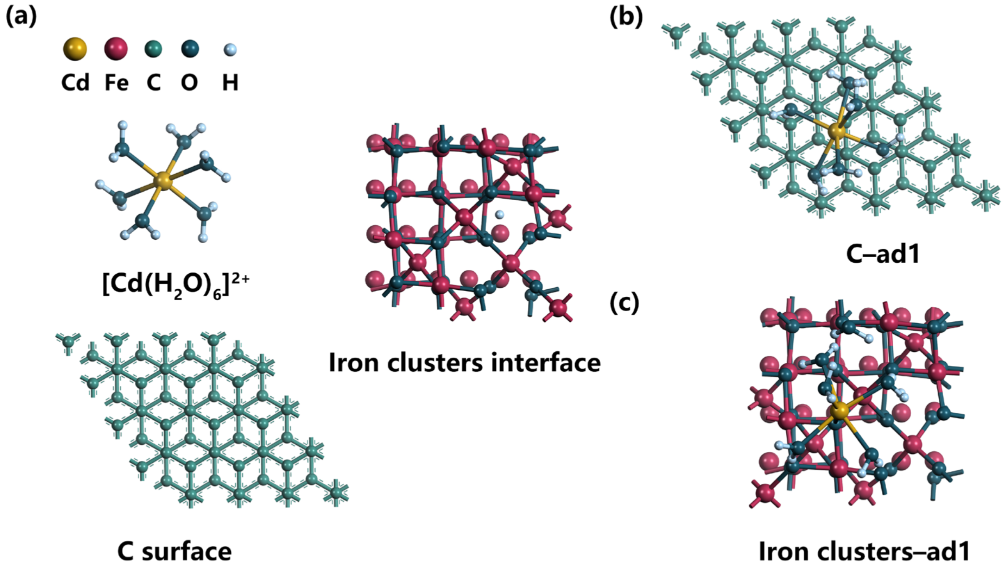

Figure S29. (a) The optimized geometries of RF-CSMSs (C surface) and RF-1.25BFA (iron clusters interface). The optimized geometries of $[Cd(H_2O)_6]^{2+}$ adsorption on (b)

C surface (C–ad1) and (c) iron clusters interface (iron clusters–ad1). All views are top views.

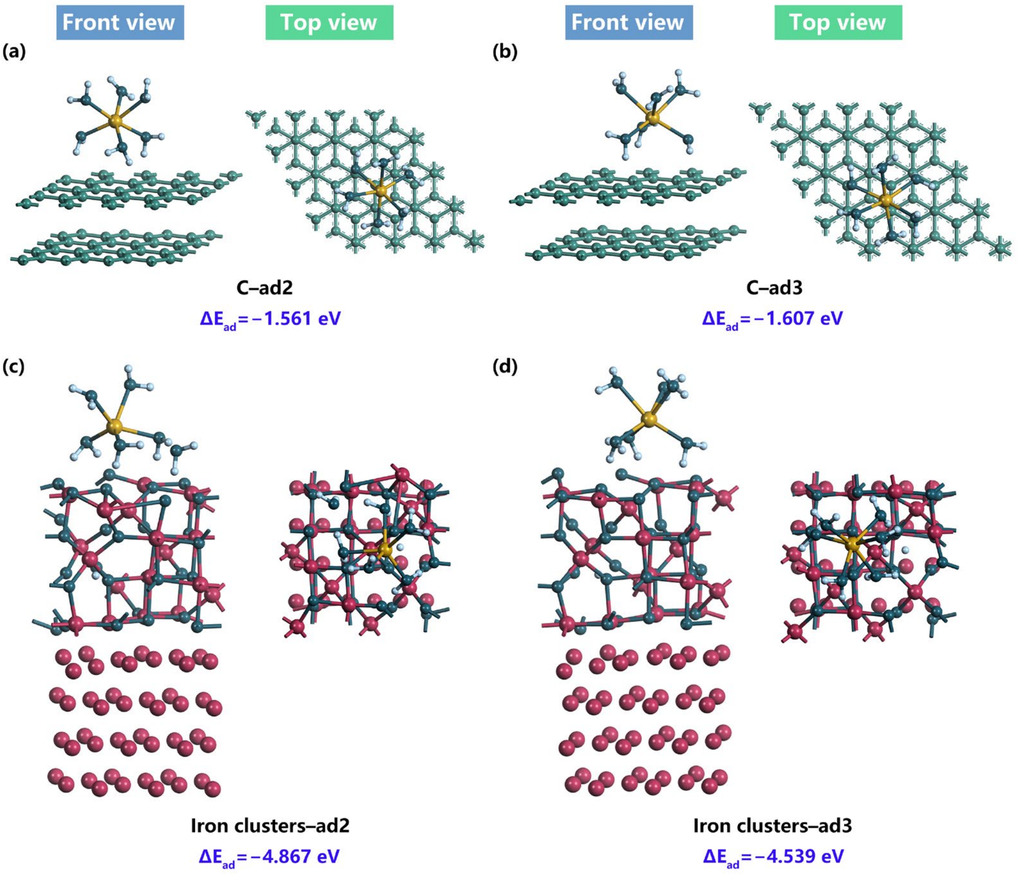

Figure S30. The optimized geometries of $[Cd(H_2O)_6]^{2+}$ adsorption on (a) C–ad2, (b) C–ad3, (c) iron clusters–ad2 and (d) iron clusters–ad3. The views include both front and top views.

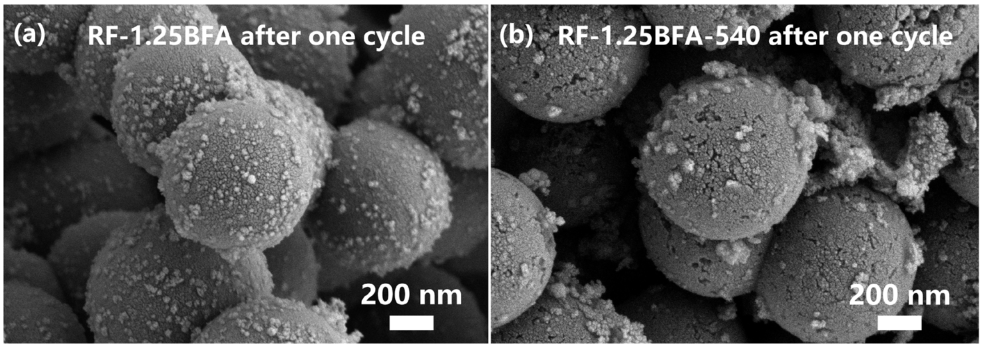

Figure S31. FE-SEM images of (a) RF-1.25BFA and (b) RF-1.25BFA-540 after one adsorption–desorption cycle for Cd(II).

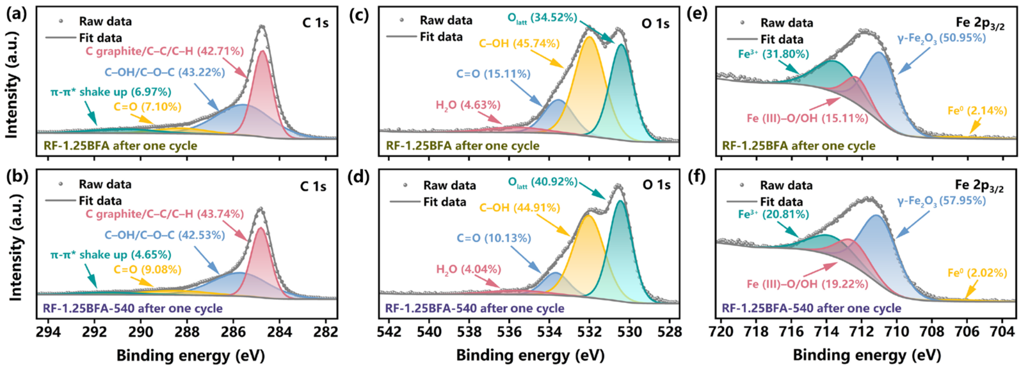

Figure S32. The high-resolution XPS spectra of (a–b) C 1 s, (c–d) O 1 s and (e–f) Fe 2 $p_{3/2}$ in RF-1.25BFA and RF-1.25BFA-540 after one adsorption–desorption cycle for Cd(II).

References

[1] a) J. Liu, S. Z. Qiao, H. Liu, J. Chen, A. Orpe, D. Zhao, G. Q. Lu, *Angew. Chem. Int. Ed.* **2011**, *50*, 5947; b) R. W. Pekala, *J. Mater. Sci.* **1989**, *24*, 3221.

[2] P. S. Kumar, L. Korving, M. C. M. van Loosdrecht, G.-J. Witkamp, *Water Res. X* **2019**, *4*, 100029.

[3] a) X. Wang, L. Meng, M. Hu, L. Gao, B. Lian, *Water Res.* **2024**, *250*, 121087; b) L. Xiong, C. Chen, Q. Chen, J. Ni, *J. Hazard. Mater.* **2011**, *189*, 741; c) E. Ugurlu, O. Aydin, M. Gencten, B. Birol, *Int. J. Environ. An. Ch.* **2024**, DOI: 10.1080/03067319.2024.23154801; d) Q. Xie, X. Ma, H. Ablat, X. Nurmamat, H. Jia, F. Wang, Z. Zhao, *Water, Air, & Soil Pollution* **2024**, *235*, 153.

[4] L. Fang, W. Yang, J. Hou, k. Zheng, A. Hussain, Y. Zhang, Z. Hou, X. Wang, *Biochar* **2023**, *5*, 69.

[5] M. Baikousi, A. B. Bourlinos, A. Douvalis, T. Bakas, D. F. Anagnostopoulos, J. Tucek, K. Safarova, R. Zboril, M. A. Karakassides, *Langmuir.* **2012**, *28*, 3918.

[6] a) C. W. Cheung, J. F. Porter, G. McKay, *Sep. Purif. Technol.* **2000**, *19*, 55; b) Y. Wu, L. Zhang, C. Gao, J. Ma, X. Ma, R. Han, *J. Chem. Eng. Data* **2009**, *54*, 3229.

[7] G. Zeng, Y. Liu, L. Tang, G. Yang, Y. Pang, Y. Zhang, Y. Zhou, Z. Li, M. Li, M. Lai, X. He, Y. He, *Chem. Eng. J.* **2015**, *259*, 153.

[8] A. Roy, J. Bhattacharya, *Chem. Eng. J.* **2012**, *211-212*, 493.

[9] a) A. Roy, J. Bhattacharya, *Sep. Purif. Technol.* **2013**, *115*, 172; b) W. Liu, J. Zhang, C. Zhang, L. Ren, *Chem. Eng. J.* **2012**, *189-190*, 295.

[10] J. Luo, X. Luo, J. Crittenden, J. Qu, Y. Bai, Y. Peng, J. Li, *Environ. Sci. Technol.* **2015**, *49*, 11115.

[11] A. Naseri, Z. Abed, M. Rajabi, B. Lal, A. Asghari, O. Baigenzhenov, S. Arghavani-Beydokhti, A. Hosseini-Bandegharaei, *Chemosphere* **2023**, *335*, 139168.

[12] A. A. Khan, R. P. Singh, *Colloids and Surfaces* **1987**, *24*, 33.

[13] V. Milman, B. Winkler, J. A. White, C. J. Pickard, M. C. Payne, E. V. Akhmatskaya, R. H. Nobes, *Int. J. Quantum. Chem.* **2000**, *77*, 895.

[14] J. P. Perdew, A. Ruzsinszky, G. I. Csonka, O. A. Vydrov, G. E. Scuseria, L. A. Constantin, X. Zhou, K. Burke, *Phys. Rev. Lett.* **2008**, *100*, 136406.

[15] J. P. Perdew, K. Burke, M. Ernzerhof, *Phys. Rev. Lett.* **1996**, *77*, 3865.

[16] A. Saeed, M. Akhter, M. Iqbal, *Sep. Purif. Technol.* **2005**, *45*, 25.

[17] N. Barka, M. Abdennouri, M. El Makhfouk, S. Qourzal, *J. Environ. Chem. Eng.* **2013**, *1*, 144.

[18] M. Cong, K. Wu, J. Wang, Z. Li, R. Mao, Y. Niu, H. Chen, *Langmuir.* **2024**, *40*, 2320.

[19] Z. Aksu, *Sep. Purif. Technol.* **2001**, *21*, 285.

[20] Y. Yan, F. Qi, L. Zhang, P. Zhang, Q. Li, *Sep. Purif. Technol.* **2022**, *297*, 121533.

[21] M. Villen-Guzman, M. M. Cerrillo-Gonzalez, J. M. Paz-Garcia, J. M. Rodriguez-Maroto, B. Arhoun, *Environ. Technol. Innovation* **2021**, *21*, 101380.

[22] E. Arslanoğlu, M. Ş. A. Eren, H. Arslanoğlu, H. Çiftçi, *Biomass Convers. Biorefin.* **2021**, *13*, 2349.

[23] X. Zheng, Q. Wu, C. Huang, P. Wang, H. Cheng, C. Sun, J. Zhu, H. Xu, K. Ouyang, J. Guo, Z. Liu, *Environ. Res.* **2023**, *231*, 116080.

[24] Y. Huang, C. Liu, L. Qin, M. Xie, Z. Xu, Y. Yu, *Molecules* **2023**, *28*, 4538.

[25] V. C. Srivastava, I. D. Mall, I. M. Mishra, *Chem. Eng. Process. Process Intensif.* **2009**, *48*, 370.

[26] U. Kumar, M. Bandyopadhyay, *Bioresour. Technol.* **2006**, *97*, 104.

[27] V. C. Srivastava, I. D. Mall, I. M. Mishra, *Chem. Eng. J.* **2006**, *117*, 79.

[28] C. Escudero, J. Poch, I. Villaescusa, *Chem. Eng. J.* **2013**, *217*, 129.

[29] R. Leyvaramos, L. Bernaljacome, I. Acostarodriguez, *Sep. Purif. Technol.* **2005**, *45*, 41.

[30] W. Zheng, X.-m. Li, F. Wang, Q. Yang, P. Deng, G.-m. Zeng, *J. Hazard. Mater.* **2008**, *157*, 490.

[31] R. Foroutan, S. J. Peighambardoust, R. Mohammadi, S. H. Peighambardoust, B. Ramavandi, *Environ. Res.* **2022**, *211*, 113020.

[32] M. D. Meitei, M. N. V. Prasad, *J. Environ. Chem. Eng.* **2013**, *1*, 200.

[33] Y. Wang, X. Meng, S. Wang, Y. Mo, W. Xu, Y. Liu, W. Shi, *Environ. Sci. Pollut. Res.* **2024**, *31*, 9237.

[34] W.-T. Tan, H. Zhou, S.-F. Tang, P. Zeng, J.-F. Gu, B.-H. Liao, *Environ. Pollut.* **2022**, *300*, 118899.

[35] J. Qu, X. Meng, X. Jiang, H. You, P. Wang, X. Ye, *J. Cleaner Prod.* **2018**, *183*, 880.

[36] S. Li, C. Luo, F. Yan, Y. Yang, B. Guo, L. Wang, S. Xu, F. Wu, P. Ji, *Environ. Pollut.* **2023**, *338*, 122638.

[37] Q. Chen, Y. Wang, G. He, M. Yilmaz, S. Yuan, *Colloids Surf., A* **2024**, *684*, 133174.

[38] S. Ge, S. Zhao, L. Wang, Z. Zhao, S. Wang, C. Tian, *Sci. Rep.* **2024**, *14*, 450.

[39] R. B. Nessim, A. R. Bassiouny, H. R. Zaki, M. N. Moawad, K. M. Kandeel, *Chem. Ecol.* **2011**, *27*, 579.

[40] R. A. Anayurt, A. Sari, M. Tuzen, *Chem. Eng. J.* **2009**, *151*, 255.

[41] Q. Li, S. Wu, G. Liu, X. Liao, X. Deng, D. Sun, Y. Hu, Y. Huang, *Sep. Purif. Technol.* **2004**, *34*, 135.

[42] P. Lodeiro, B. Cordero, J. Barriada, R. Herrero, M. Sastredevicente, *Bioresour. Technol.* **2005**, *96*, 1796.

[43] P. X. Sheng, Y.-P. Ting, J. P. Chen, L. Hong, *J. Colloid Interface Sci.* **2004**, *275*, 131.

[44] F. Lyu, H. Yu, T. Hou, L. Yan, X. Zhang, B. Du, *J. Colloid Interface Sci.* **2019**, *539*, 184.

[45] K. Naseem, R. Begum, W. Wu, M. Usman, A. Irfan, A. G. Al-Sehemi, Z. H. Farooqi, *J. Mol. Liq.* **2019**, *277*, 522.

[46] C. Li, H. Duan, X. Wang, X. Meng, D. Qin, *Chem. Eng. J.* **2015**, *262*, 250.

[47] M. Chen, J. Liu, Y. Bi, S. Rehman, Z. Dang, P. Wu, *J. Hazard. Mater.* **2020**, *388*, 122078.

[48] X. Liu, J. Guan, G. Lai, Q. Xu, X. Bai, Z. Wang, S. Cui, *J. Cleaner Prod.* **2020**, *253*, 119915.

[49] K. Wang, J. Gu, N. Yin, *Ind. Eng. Chem. Res.* **2017**, *56*, 1880.

[50] M. Kumar, B. P. Tripathi, V. K. Shahi, *J. Hazard. Mater.* **2009**, *172*, 1041.

[51] G. N. Manju, K. Anoop Krishnan, V. P. Vinod, T. S. Anirudhan, *J. Hazard. Mater.* **2002**, *91*, 221.

[52] V. Singh, A. K. Sharma, S. Maurya, *Ind. Eng. Chem. Res.* **2009**, *48*, 4688.

[53] R. Karthik, S. Meenakshi, *Int. J. Biol. Macromol.* **2015**, *78*, 157.

[54] D. Liu, Z. Li, Y. Zhu, Z. Li, R. Kumar, *Carbohyd. Polym.* **2014**, *111*, 469.

[55] N. Ferrah, O. Abderrahim, M. A. Didi, D. Villemin, *J. Chem.* **2013**, *2013*, 980825.

[56] H. Yu, L. Zheng, T. Zhang, J. Ren, W. Cheng, L. Zhang, P. Meng, *Environ. Res.* **2021**, *195*, 110848.

[57] X. Yuan, N. An, Z. Zhu, H. Sun, J. Zheng, M. Jia, C. Lu, W. Zhang, N. Liu, *Process. Saf. Environ.* **2018**, *119*, 320.

[58] L. R. Rad, A. Momeni, B. F. Ghazani, M. Irani, M. Mahmoudi, B. Noghreh, *Chem. Eng. J.* **2014**, *256*, 119.

[59] M. R. Awual, M. Khraisheh, N. H. Alharthi, M. Luqman, A. Islam, M. Rezaul Karim, M. M. Rahman, M. A. Khaleque, *Chem. Eng. J.* **2018**, *343*, 118.

[60] D. P. Sounthararajah, P. Loganathan, J. Kandasamy, S. Vigneswaran, *J. Hazard. Mater.* **2015**, *287*, 306.

[61] L. S. Alqarni, A. A. Alshahrani, H. Alhussain, N. Y. Elamin, M. Ismail, K. K. Taha, A. Modwi, *Diam. Relat. Mater.* **2024**, *142*, 110803.

[62] A. Modwi, M. Ismail, H. Idriss, M. A. B. Aissa, L. Khezami, M. Bououdina, H. Qian, *J. Nanomater.* **2022**, *2022*, 1458442.

[63] O. Aldaghri, A. Modwi, H. Idriss, M. K. M. Ali, K. H. Ibnaouf, *Diam. Relat. Mater.* **2022**, *129*, 109315.

[64] S. S. Hosseini, A. Hamadi, R. Foroutan, S. J. Peighambardoust, B. Ramavandi, *J. Water Process Eng.* **2022**, *48*, 102911.

[65] T. Mathialagan, T. Viraraghavan, *J. Hazard. Mater.* **2002**, *94*, 291.

[66] X. Guan, X. Yuan, Y. Zhao, J. Bai, Y. Li, Y. Cao, Y. Chen, T. Xiong, *J. Colloid Interface Sci.* **2022**, *612*, 572.

[67] M. Torab-Mostaedi, H. Ghassabzadeh, M. Ghannadi-Maragheh, S. J. Ahmadi, H. Taheri, *Braz. J. Chem. Eng.* **2010**, *27*, 299.

[68] H. Chen, R. Xiao, D. Huang, R. Deng, R. Li, Y. Chen, W. Zhou, *J. Mol. Liq.* **2024**, *400*, 124478.

[69] S.-h. Ju, S.-d. Lu, J.-h. Peng, L.-b. Zhang, C. Srinivasakannan, S.-h. Guo, W. Li, *T. Nonferr. Metal Soc.* **2012**, *22*, 3140.

[70] Z. Orolínovaá, A. Mockovčiaková, J. Škvarla, *Desalin. Water Treat.* **2012**, *24*, 284.

[71] K. O. Adebowale, I. E. Unuabonah, B. I. Olu-Owolabi, *J. Hazard. Mater.* **2006**, *134*, 130.

[72] L. Lv, G. Tsoi, X. S. Zhao, *Ind. Eng. Chem. Res.* **2004**, *43*, 7900.

[73] M. Allawzi, S. Al-Asheh, *Desalin. Water Treat.* **2010**, *22*, 349.

[74] F. He, B. Ma, C. Wang, Y. Chen, X. Hu, *Sep. Purif. Technol.* **2023**, *310*, 123234.

[75] X. Liu, H. Yin, H. Liu, Y. Cai, X. Qi, Z. Dang, *J. Hazard. Mater.* **2023**, *443*, 130167.

[76] X. Yuan, D. Zhou, W. Xia, J. An, X. Zhou, J. Yin, *Desalin. Water Treat.* **2020**, *192*, 271.

[77] T. M. T. Nguyen, T. P. T. Do, T. S. Hoang, N. V. Nguyen, H. D. Pham, T. D. Nguyen, T. N. M. Pham, T. S. Le, T. D. Pham, *Int. J. Polym. Sci.* **2018**, *2018*, 2830286.

[78] C. Wang, H. Yin, L. Bi, J. Su, M. Zhang, T. Lyu, M. Cooper, G. Pan, *J. Hazard. Mater.* **2020**, *384*, 121461.

[79] C.-Y. Cao, J. Qu, F. Wei, H. Liu, W.-G. Song, *ACS Appl. Mater. Interfaces* **2012**, *4*, 4283.

[80] Y. Zhang, Y. Li, C. Dai, X. Zhou, W. Zhang, *Chem. Eng. J.* **2014**, *244*, 218.

[81] Lalchhingpuii, D. Tiwari, Lalhmunsiama, S. M. Lee, *Chem. Eng. J.* **2017**, *328*, 434.

[82] E. J. Kim, C. S. Lee, Y. Y. Chang, Y. S. Chang, *ACS Appl. Mater. Interfaces* **2013**, *5*, 9628.

[83] M. Xu, Y. Zhang, Z. Zhang, Y. Shen, M. Zhao, G. Pan, *Chem. Eng. J.* **2011**, *168*, 737.

[84] F. Ge, M. M. Li, H. Ye, B. X. Zhao, *J. Hazard. Mater.* **2012**, *211-212*, 366.

[85] T. Madrakian, A. Afkhami, B. Zadpour, M. Ahmadi, *J. Ind. Eng. Chem.* **2015**, *21*, 1160.

[86] X. Zhu, T. Song, Z. Lv, G. Ji, *Process. Saf. Environ.* **2016**, *104*, 373.

[87] Z. Li, L. Wang, J. Meng, X. Liu, J. Xu, F. Wang, P. Brookes, *J. Hazard. Mater.* **2018**, *344*, 1.

[88] J.-f. Liu, Z.-s. Zhao, G.-b. Jiang, *Environ. Sci. Technol.* **2008**, *42*, 6949.

[89] H. Chen, F. Xu, Z. Chen, O. Jiang, W. Gustave, X. Tang, *J. Environ. Sci.* **2020**, *96*, 186.

[90] H. Karami, *Chem. Eng. J.* **2013**, *219*, 209.

[91] Q. Su, B. Pan, S. Wan, W. Zhang, L. Lv, *J Colloid Interface Sci* **2010**, *349*, 607.

[92] M. Li, M.-y. Li, C.-g. Feng, Q.-x. Zeng, *Appl. Surf. Sci.* **2014**, *314*, 1063.

[93] G. Zhou, C. Liu, Y. Tang, S. Luo, Z. Zeng, Y. Liu, R. Xu, L. Chu, *Chem. Eng. J.* **2015**, *280*, 275.

[94] Y. Li, J. He, K. Zhang, T. Liu, Y. Hu, X. Chen, C. Wang, X. Huang, L. Kong, J. Liu, *RSC Adv.* **2018**, *9*, 397.

[95] I. Shahzadi, Y. Wu, H. Lin, J. Huang, Z. Zhao, C. Chen, X. Shi, H. Deng, *J. Hazard. Mater.* **2023**, *453*, 131312.

[96] J. Ma, G. Zhou, L. Chu, Y. Liu, C. Liu, S. Luo, Y. Wei, *ACS Sustainable Chem. Eng.* **2016**, *5*, 843.

[97] M. F. Hamza, N. A. Hamad, D. M. Hamad, M. S. Khalafalla, A. A. H. Abdel-Rahman, I. F. Zeid, Y. Wei, M. M. Hessien, A. Fouda, W. M. Salem, *Materials* **2021**, *14*, 2189.

[98] D. Kundu, S. K. Mondal, T. Banerjee, *J. Chem. Eng. Data* **2019**, *64*, 2601.

[99] F. M. Koehler, M. Rossier, M. Waelle, E. K. Athanassiou, L. K. Limbach, R. N. Grass, D. Gunther, W. J. Stark, *Chem. Commun.* **2009**, *32*, 4862.

[100] J. Li, C. Chen, K. Zhu, X. Wang, *J. Taiwan Inst. Chem. Eng.* **2016**, *59*, 389.

[101] S. Wu, K. Zhang, X. Wang, Y. Jia, B. Sun, T. Luo, F. Meng, Z. Jin, D. Lin, W. Shen, L. Kong, J. Liu, *Chem. Eng. J.* **2015**, *262*, 1292.

[102] G. Zhao, J. Li, X. Ren, C. Chen, X. Wang, *Environ. Sci. Technol.* **2011**, *45*, 10454.

[103] M. Musielak, A. Gagor, B. Zawisza, E. Talik, R. Sitko, *ACS Appl. Mater. Interfaces* **2019**, *11*, 28582.

[104] L. M. Huong, D. B. Thinh, T. H. Tu, N. M. Dat, T. T. Hong, P. T. N. Cam, D. N. Trinh, H. M. Nam, M. T. Phong, N. H. Hieu, *Surf. Interfaces* **2021**, *26*, 101309.

[105] X. Li, H. Zhou, W. Wu, S. Wei, Y. Xu, Y. Kuang, *J. Colloid Interface Sci.* **2015**, *448*, 389.

[106] X. Li, S. Wang, Y. Liu, L. Jiang, B. Song, M. Li, G. Zeng, X. Tan, X. Cai, Y. Ding, *J. Chem. Eng. Data* **2016**, *62*, 407.

[107] C. D. Ghugare, N. V. Rathod, A. Rao, J. S. Jadhao, S. M. Chavan, A. V. Kubade, V. S. Kalyani, A. B. Patil, *Separ. Sci. Technol.* **2024**, *59*, 241.

[108] Y.-H. Li, J. Ding, Z. Luan, Z. Di, Y. Zhu, C. Xu, D. Wu, B. Wei, *Carbon* **2003**, *41*, 2787.

[109] C. Luo, R. Wei, D. Guo, S. Zhang, S. Yan, *Chem. Eng. J.* **2013**, *225*, 406.

[110] G. D. Vuković, A. D. Marinković, M. Čolić, M. Đ. Ristić, R. Aleksić, A. A. Perić-Grujić, P. S. Uskoković, *Chem. Eng. J.* **2010**, *157*, 238.

[111] N. Sankararamakrishnan, M. Jaiswal, N. Verma, *Chem. Eng. J.* **2014**, *235*, 1.

[112] M. A. Tofighy, T. Mohammadi, *J. Hazard. Mater.* **2011**, *185*, 140.

[113] Y.-H. Li, S. Wang, Z. Luan, J. Ding, C. Xu, D. Wu, *Carbon* **2003**, *41*, 1057.

[114] L. Zhou, N. Li, G. Owens, Z. Chen, *Chem. Eng. J.* **2019**, *362*, 628.

[115] S. Li, Y. Gan, S. J. Shah, R. Wang, W. Gong, R. Wei, H. Ji, Z. Zhao, Z. Zhao, *Chem. Eng. J.* **2021**, *426*, 131440.

[116] Y. Feng, G. Chen, Y. Zhang, D. Li, C. Ling, Q. Wang, G. Liu, *J. Hazard. Mater.* **2022**, *424*, 127362.

[117] X. L. Chen, F. Li, X. J. Xie, Z. Li, L. Chen, *Int. J. Environ. Res. Public Health* **2019**, *16*, 3046.

[118] Z. Li, M. Li, Z. Wang, X. Liu, *Chem. Eng. J.* **2020**, *381*, 122785.

[119] J. Deng, Y. Liu, S. Liu, G. Zeng, X. Tan, B. Huang, X. Tang, S. Wang, Q. Hua, Z. Yan, *J. Colloid Interface Sci.* **2017**, *506*, 355.

[120] D. Lv, Y. Liu, J. Zhou, K. Yang, Z. Lou, S. A. Baig, X. Xu, *Appl. Surf. Sci.* **2018**, *428*, 648.

[121] X. Zhang, L. Zhang, A. Li, *J. Environ. Manage.* **2018**, *206*, 989.

[122] J. Wei, C. Tu, G. Yuan, D. Bi, L. Xiao, B. K. G. Theng, H. Wang, Y. S. Ok, *J. Hazard. Mater.* **2019**, *368*, 541.

[123] A. G. Karunanayake, O. A. Todd, M. Crowley, L. Ricchetti, C. U. Pittman, R. Anderson, D. Mohan, T. Mlsna, *Chem. Eng. J.* **2018**, *331*, 480.

[124] S. Singh, A. G. Anil, T. S. S. K. Naik, B. U, S. Khasnabis, B. Nath, V. Kumar, S. Subramanian, J. Singh, P. C. Ramamurthy, *J. Water Process Eng.* **2022**, *47*, 102723.
